# Supplementary material for: Development of behavioral rules for upstream orientation of fish in confined space
Source: PLoS One. 2022 Feb 18;17(2):e0263964. doi: 10.1371/journal.pone.0263964 (PMC8856537; doi:10.1371/journal.pone.0263964)
Supplement: S4 Appendix — (PDF) [file pone.0263964.s004.pdf]

Supporting Information Appendix: Behavioral model description

David C. Gisen et al.

This behavior model description follows the ODD (Overview, Design concepts, Details) protocol for describing individual- and agent-based models (Grimm et al. 2006), as updated by Grimm et al. (2020).

Contents

1 Purpose and patterns **2**

2 Entities, state variables, and scales **2**

3 Process overview and scheduling **3**

4 Design concepts **3**
4.1 Basic principles . . . 3
4.2 Emergence . . . 4
4.3 Adaptation . . . 4
4.4 Prediction . . . 5
4.5 Sensing . . . 5
4.6 Interaction . . . 6
4.7 Stochasticity . . . 6
4.8 Observation . . . 6

5 Initialization **7**

6 Input data **7**

7 Submodels **7**
7.1 getSensoryPoints and interpolateEnvironment . . . 7
7.2 chooseBehavior . . . 8
7.3 Motivation . . . 9
7.4 Fatigue . . . 10
7.5 Migrating . . . 10
7.6 Holding . . . 12
7.7 Drifting . . . 12
7.8 Vertical swimming . . . 12
7.9 updateFishLocation . . . 13
7.10 resetPosition . . . 14

References **15**

Online resources **16**

1 Purpose and patterns

Our long-term purpose is to find possible stimulus-response relations in fish to enable predictions of fishway attraction and passage. The main objective of the present behavioral model is to evaluate the suitability of a baseline stimulus, the additional hydraulic stimuli velocity magnitude, acceleration, and TKE, and an additional wall distance stimulus, for explaining orientation and navigation of upstream moving brown trout in a flume. The behavior patterns used for development describe spatial behavior and overall success. In particular, they describe lateral and vertical distributions, area and occurrence of turns, and the rate of arriving at the upstream flume end. Timing and interaction were not used as patterns. The model's parameterization is directed to brown trout in a confined hydraulic flume. Still, its structural design allows to include both different species and natural river flow fields in future versions.

2 Entities, state variables, and scales

Individuals in the model represent brown trout as spatially explicit points in a 3D laboratory flume filled with flowing water. Their movement is kinematic, i.e. there are no forces acting between fish and flow field and fish have no mass. All fish have the same length. They can be distinguished by a number of *state variables* (Table 1) which are modified through distinct *behaviors*. The key state variables are *position* (in continuous space), *fatigue*, and *motivation* (to swim upstream). Motivation increases either "fast" or "slow", depending on the initially defined motivatability.

The laboratory flume overall domain size is (length × width × height) 16.78 m × 2.50 m × 1.35 m, including areas behind the screen and air which are not accessible to the fish. Water depth is $h = 0.60$ m. The domain is divided into 848 694 mostly cubic cells, a typical cell edge length being $\Delta(x/y/z) = 0.05$ m. Local refinements up to $\Delta(x/y/z) = 0.0125$ m at the slot and screen posts capture finer flow features. Each cell[1] holds information on the state variables listed in Table 2. They are taken from the CFD (computational fluid dynamics) model in steady state. The CFD model x axis points downstream in flume longitudinal direction, the y axis points in lateral direction, and the z axis points against gravity.

Table 1: State variables defining individual fish.

Symbol	Units	Description
ID	–	Unique identification number (constant)
$\vec{s}$	m	Position point in 3D space
M	–	Motivation to swim upstream, takes values in $[0, 1]$
F	–	Fatigue, takes values in $[0, 1]$
M^*	–	Motivatability, either "fast" or "slow" (constant)
U_s	m/s	Swim speed magnitude, relative to flow
γ_s	°	Horizontal swim angle, in CFD model x/y plane, takes values in $[0°, 360°)$
β_s	°	Vertical swim angle, in CFD model x/z plane, takes values in $[-90°, 90°]$
$\vec{s}_{avg}$	m	Acclimatized position in 3D space
$t_{sameSpot}$	s	Time cumulatively spent within radius $r_{sameSpot}$ around position $\vec{s}_{avg}$

Each discrete time step is worth $\Delta t = 0.5$ s of real time. The total model run time is $N_{\Delta t} = 7200$ time steps, which equals a total clock time of $t_{total} = 3600$ s, or 60 min. This equals the maximum laboratory trial duration. The time step width is in between studies with more complex hydraulics at $\Delta t = 0.01$ s (Smith et al. 2014) and a much wider headwater at $\Delta t = 2.0$ s (Goodwin et al. 2014a).

Time perception of the model fish depends on the time step width. Goodwin et al. (2014b) suggested the use of sub-time-steps to increase update frequency of internal states for faster adaptation in high-acceleration zones. Using sub-time-steps was not

[1] The software OpenFOAM stores most field information on cell faces, not centers, but enables free interpolation in space.

Table 2: State variables defining cells of the CFD model.

Variable	Units	Description
Cell label	–	Unique identification number, provides coordinate access
Patch type	–	"wall", "genericPatch", "patch"
α	–	Fraction of cell volume filled with water
$\vec{U}_m$	m/s	3D flow vector, averaged to steady state, also used as combination of magnitude U_m, horizontal angle γ_m $[0°, 360°]$, and vertical angle β_m $[-90°, 90°]$
$\|a\|$	m/s^2	Advective acceleration magnitude
k	m^2/s^2	Turbulence kinetic energy (also: TKE)

tested, but could be a way to reduce computational cost while increasing temporal accuracy.

3 Process overview and scheduling

In the beginning of every time step, all fish collect local environment information (e.g. flow velocity). Next, they choose one of three horizontal *behaviors*, which are migrating, holding, and drifting (details in section 7). An additional vertical behavior is executed independently of the horizontal behavior chosen. The behaviors and the flow field primarily act on the state variable position, and thus indirectly on the state variables motivation and fatigue.

The state variables are stored in arrays which preserve all time steps for post-process reconstruction. The general schedule of updating all state variables, which takes place every time step, is summarized following the program structure in pseudo-code as:

```
for all fish{
    getSensoryPoints;
    interpolateEnvironmentToSensoryPoints;
    computeSwimAngleAndVelocity(
        chooseBehavior;
        if (B==1) migrating;
        if (B==2) holding;
        if (B==3) drifting;
        verticalSwimming;
    );
    updateFishLocation(
        if (locationOutsideMesh)
        then resetPosition;
    );
}
```

Updating the state variables is performed consecutively per single fish during a given time step. Still, the process can be described as synchronous updating, as all input is read from the previous time step. The current time step holds the initial position and the new swim angle and swim speed influenced by the previous time step. The new position (after movement) is assigned to the following time step, making it the only variable in the model which is updated in advance. Individuals do not interact in the model, but even with interaction, individual order would not matter through this three-time-step approach (adopted from Goodwin et al. 2014b). Figure 1 visualizes an example model process.

4 Design concepts

4.1 Basic principles

The behavioral model exhibits three basic principles, which mainly steer movement in the three spatial dimensions.

The first basic principle is to summarize all fish characteristics possibly acting on longitudinal movement by the two opposing variables motivation (to swim upstream), M, and fatigue, F. They resemble the classic model components "need" and "cost" (Willis

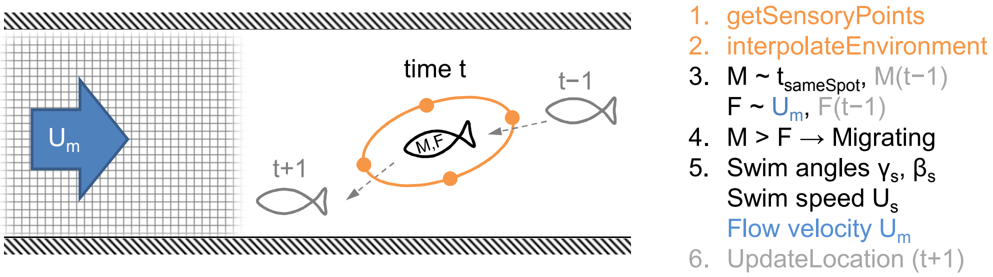

Figure 1: Schematic 2D example of the model process during a typical time step t in which *migrating* behavior is chosen.

2011). The classic risk component (e.g., predation) is not considered. We assume that motivation (for swimming upstream) increases if the fish makes no progress, according to the model purpose of finding behavioral rules for upstream migration. We model fatigue in relation to relative swim speed, but acknowledge that a more realistic approach (e.g., Castro-Santos 2005) could prove useful (section 7.4).

The second basic principle is to steer horizontal upstream orientation based on an external stimulus. We presume a baseline orientation against the flow vector as rheotaxis is fundamental for upstream movement (Elder and Coombs 2015). This behavior also ensures a somewhat continuous fish movement, as a steady flow does not exhibit sudden direction changes. A horizontal swim angle component is added depending on the active stimulus behavioral rule. We chose the hydraulic stimuli of decreasing velocity magnitude (Zielinski et al. 2018), increasing acceleration (Goodwin et al. 2014a), and constant TKE (Gao et al. 2016). Decreasing wall distance, which can be perceived visually (Liao 2007) or by hydraulic means, was the final stimulus tested.

Stimulus gradients, as used here, are considered a natural cue for orientation, as they can be sensed independently of an external reference frame. In the case of acceleration, the preferred direction of the gradient was not clear from the literature. We decided for an increasing gradient, as it resembled our observed lateral distribution pattern.

The third basic principle is vertical orientation by hydrostatic pressure. Following Goodwin et al. (2014a), we presume that trout adapt their swim bladder to maintain an acclimatized hydrostatic pressure, simplified as elevation coordinate.

4.2 Emergence

A main result, the lateral distribution of trout (pattern P1), emerges mainly from a combination of the horizontal stimulus, swim angles, and rheotaxis. Three stimuli (velocity, acceleration, wall distance) are based on following a gradient. They carry the risk of imposing (Grimm and Railsback 2005) large values of the near-wall zones of pattern P1. However, there are two mechanisms to counter this risk by moving fish to the middle zone, namely the random overlay angle and inclined drift.

Vertical behavior depends on acclimatized pressure/elevation and is not expected to vary strongly because of the shallow water (P2).

The location and frequency of turns (measured by P3 and P4) emerges from hydraulic conditions and internal state parameters, primarily motivation and fatigue.

Overall navigation, as measured by P5, emerges from orientation, the motivation/fatigue balance, and hydraulics along the paths through the flume.

4.3 Adaptation

Model fish adapt to changes in the environment (flow field) through fatigue, and adapt to “waiting time” (time without upstream progress) through motivation. The latter one is indirectly related to fitness-seeking, as upstream migration provides many opportunities to support the fish’s fitness. Real-world examples include both pushing and pulling factors, e.g. reproduction, feeding, and refuge seeking (Lucas and Baras 2001, p. 5). We model it as a pure pushing factor. We do not assume implicit resemblence of real migration driving behavior, as the flume environment seems to be too artifical for this purpose.

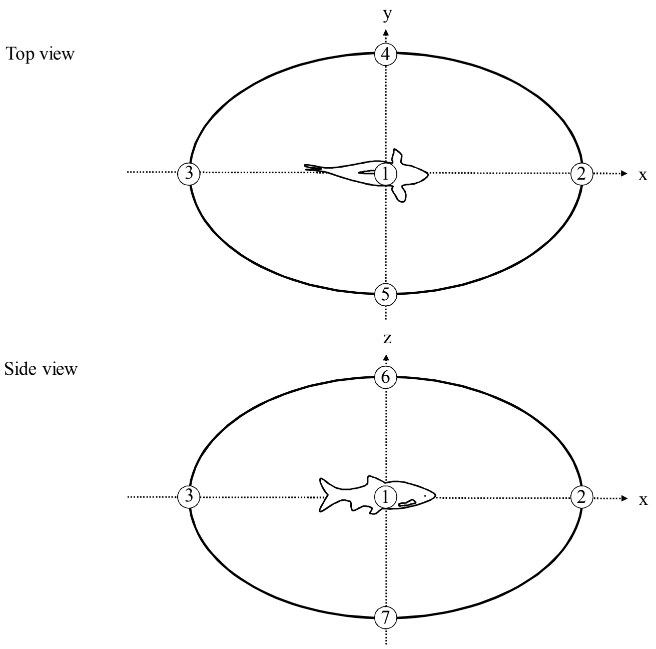

Figure 2: Fish sensory ovoid schematic showing the local coordinate system and all seven sensory points (SP). The exact shape of the ovoid is variable.

Transport of information through time is realized by using memory coefficients to model a moving average of a given parameter. Motivation and fatigue strongly depend on their previous values and thus on the flow field along the path the fish took. They drive the streamwise migration behavior. Motivation also depends on position memory. There is evidence that some fish species are able to memorize locations and use landmarks for orientation in controlled experiments (Odling-Smee and Braithwaite 2003). In the limited extend of a flume with clear water it is plausible that fish have a good sense of orientation and remember the locations they have been in. This is used in the model to increase motivation if the position keeps unchanged for too long, representing the presumed basic motivation for migration.

In contrast to approaches forcing the fish to constantly swim upstream until failure, the chosen approach allows model fish to drift, rest, and repeat an attempt (trial repetition). This is important here, because if a fishway is the only way upstream, a fish must not give up after the first failure to use it to be successful. Indeed, repeated up- and downstream movement was observed in pattern P3 of our real brown trout data set. However, fish do not learn from successful or unsuccessful paths in the model, which could be assumed for real fish.

The ODD design concepts "objectives" and "learning" were not used in this model.

4.4 Prediction

Prediction is modeled implicitly in the migrating behavior and its varying stimuli, which are thought to implicitly signal favorable flow conditions. For example, lower velocity magnitude is associated with lower swim energy cost (Wang and Chanson 2018).

4.5 Sensing

Information from the CFD model is interpolated from its discrete numerical mesh to points relative to the fish position. This concept is adapted from Goodwin et al. (2014b) and termed the *sensory ovoid.* It approximates real sensing, mediated by e.g. the lateral line, vestibular systems, vision, swim bladder, and tactile sense (Denton and Gray 1988; Liao 2007; Bleckmann and Zelick 2009; Kasumyan 2011).

The sensory ovoid surrounds the fish and is discretized by seven sensory points, SP(1–7) (six in the ovoid and one in the center, Figure 2).

The interface between the conceptual elements "environment" and "fish" is the fish's skin. As the fish is represented as a point in the model, its perception range would be under-estimated without the sensory ovoid. The true range is unknown, but likely to vary with a lot of factors.

Individuals in the model perceive the mean velocity as a three-component vector, and acceleration magnitude, TKE and their elevation (vertical coordinate) as scalars. We assume that velocity direction information is always available, either mediated by the lateral line system (Bleckmann and Zelick 2009) or by tactile reception (Kasumyan 2011). This would be erroneous in stillwater or highly turbulent flows without a clear main direction, but is assumed valid for our flume.

Model fish also sense the front and side distance to surrounding walls through the sensory ovoid. This can be interpreted as seeing, touching, or hydraulically sensing the boundary.

Regarding internal stimuli, model fish sense their time-averaged motivation and fatigue at any time step.

Model fish are not aware of their companions. Even if simulated together, they behave independently.

4.6 Interaction

The actual behavioral rules leading to fish shoaling and schooling are still poorly understood, even though different proposals have been made and main features of schools were successfully reproduced in the past decades (Lopez et al. 2012). Because no clear shoaling pattern was identified for the trout observed, and the underlying model framework as well as pre- and post-processing would require considerable adaptation, interaction was not modeled.

4.7 Stochasticity

Stochasticity is introduced at multiple points to recreate unpredictable behavior variation which is not represented mechanistically, because details of these mechanisms are either assumed to be irrelevant to our purpose, or are simply unknown (Grimm and Railsback 2005). This also encompasses behavioral responses to variations in the (real) transient flow field, which are not represented in our time-averaged (steady-state) model flow field.

The pseudo-random number generator implemented in the model uses a fixed initial seed number in [1,999] to allow reproduction of results. All three horizontal behaviors execute the same amount of calls to the random number generator. This is important for comparison of different model runs, where behavior calls could differ, as pseudo-random numbers depend on their previous values.

In *migrating* behavior, the horizontal swim angle is modified randomly to avoid path repetition if starting from the same position.

After being stuck for too long in the same place despite *migrating* behavior is active, the fish select new horizontal and vertical swim angles randomly to escape.

The general drifting direction is the flow direction. The amount of retardation by slowly swimming against the flow and the side and amount of sidewards drifting are determined randomly as their mechanisms are both unknown and not relevant for our purpose.

The ODD design concept "collectives" was not used in this model.

4.8 Observation

Primary model outputs are the position and swim vectors of individual fish. From these data, pattern metrics are computed during postprocessing using the same methods as for the laboratory patterns. They include the time fraction spent in lateral, longitudinal, and vertical flume zones, swim direction changes, and the portion of active fish crossing the most upstream control line.

5 Initialization

The flow field values are read from a completed CFD model simulation. Results are steady, i.e. there is no change in the flow field over time. Turbulence is approximated through turbulence kinetic energy (TKE) output of the CFD model.

Individual positions for $n_{fish} = 100$ fish are initially set at $x = 11.01$ m in the start area in a lateral distance of $y = 0.15$ m to either wall and close to the bottom according to observations ($z = 0.07$ m). The fish count is an arbitrary compromise between accuracy of the average results and computational cost. The initial side distribution is a model parameter (number of fish on the left flume side, facing downstream, divided by the total number of fish - L/n), to allow testing of potential influence on the results.

The initial horizontal swim angle is set to $\gamma_s = 180°$ against the CFD mesh x direction, i.e. fish face upstream. Initial vertical swim angle is $\beta_s = 0°$. The sensory ovoid point positions are not computed before the first time step. Fish ID values are read and remain constant during the simulation. The state variable value for motivation is M_{ini} (Table 3). All remaining fish state variables (Table 1) are initialized to zero.

Fish are assigned one of two categories termed "fast" and "slow". The category determines the motivation parameters, $k_{M,f}$ and $k_{M,s}$ (section 7). In this way we summarize all individual traits which could influence migratory tendencies, such as being either bold or shy (Chapman et al. 2011) or being physically strong or weak. The share and side distribution of fast and slow fish is also steered by model parameters for the left and right flume side, sL/L and sR/R.

6 Input data

The model does not use data from external sources to represent time-varying processes.

7 Submodels

All processes in the behavioral model can be understood as distinct submodels representing certain traits or behaviors of the individuals. The submodels are mathematically described in this section; all input parameters and initially chosen values for trout are listed in Table 3.

7.1 getSensoryPoints and interpolateEnvironment

The seven sensory points of the sensory ovoid are defined relative to the fish's center position. The outer points are placed along the three principal axis of the fish in each direction (Figure 2). A local coordinate system is defined by swimming direction and opposite gravity direction, with the origin being in the fish's center, $\vec{s}$. The distances from the center are O_X, O_Y, O_Z, where the indices denote local axes (Table 3). The maximum wall detection range is r_{wall}.

If the computed sensory point happens to be outside the computational domain, it is moved iteratively by thirds of the distance towards the fish center, which is controlled to be always inside the domain, until it is inside the domain. If the computed sensory point lies within the air phase, it is moved down in quarters of the distance towards the fish center until is is inside the water phase.

Hydraulic variables of the CFD model (Table 2) are interpolated to all sensory points at the beginning of every time step. Wall distances are just determined for the front and side sensory points.

Rationale: The values chosen for the ovoid axes lengths reflect the longish form of trout, but are a bit wider and higher than measured to produce a larger sensing range. Different estimations are found in the literature: Ovoid size has been modeled as randomly fluctuating in Gao et al. (2016) and additionally dependent on flow acceleration in Goodwin et al. (2014a), which both was found not necessary here. For comparison: Goodwin et al. (2006) used an estimate of $O_{X,Y,Z} = 1.25 - 1.875$ m (14–21 BL) and Kerr et al. (2016) used a rectangle of $O_X = 2.0$ BL $\times$ $O_Y = 1.0$ BL (BL $= 0.11$–0.29 m).

Wall detection range is estimated based on the assumption that it is mediated visually. We estimate it to $r_{wall} = 4$ m in all directions for our clear and light flume water.

Table 3: Behavioral model parameters and their values in the initial parameter set. Values which were measured or estimated from literature are referenced, the others were guessed and/or determined by trial-and-error. All parameters varied during parameter set generation are numbered in the first column. Also, their estimated sensitivity range is given. Detailed descriptions can be found in the ODD sections referenced in the last column. Horizontal sections are (1) fixed for all model runs, (2) varied general parameters, (3) varied individual parameters that were used for single stimulus versions, and (4) varied initialization parameters.

No.	Parameter name	Symbol	Value	Units	Range	Section
	Body length of our trout[1]	BL	0.27	m		7.4
	Ground speed[2]	U_g	2.5	$\frac{\text{BL}}{\text{s}}$		7.5
	Sensory ovoid size, longitudinal	O_X	0.50	BL		7.1
	Sensory ovoid size, lateral	O_Y	0.25	BL		7.1
	Sensory ovoid size, vertical	O_Z	0.33	BL		7.1
	Radius limit from initial position	$r_{sameSpot}$	3.0	–		7.3
	Drift deviation angle	γ_{drift}	90	°		7.7
1	Motivation initial value	M_{ini}	0.2	-	0–0.5	5
2	Motivation memory coefficient	m_M	0.95	-	0.92–0.97	7.3
3	Motivation denominator (“fast fish”)	$k_{M,f}$	20	s	2–32	7.3
4	Motivation denominator (“slow fish”)	$k_{M,s}$	130	s	90–140	7.3
5	Spot memory coefficient	m_s	0.94	-	0.88–0.98	7.3
6	Fatigue (decreasing) memory coefficient	$m_{F,d}$	0.99	-	0.95–1.00	7.4
7	Fatigue (increasing) memory coefficient	$m_{F,i}$	0.30	-	0.24–0.34	7.4
8	Fatigue denominator[3]	k_F	25	$\frac{\text{BL}}{\text{s}}$	19–29	7.4
9	Holding behavior extent	Δ_{hold}	0.05	-	0.02–0.07	7.2
10	Drifting behavior straight drift probability	$k_{drift,s}$	0.95	-	0.75–1.00	7.7
11	Vertical behavior elevation threshold	k_p	0.14	m/s	0.05–0.20	7.8
12	Vertical behavior correction angle	β_p	14	°/s	5–20	7.8
13	Stuck time threshold	t_{stuck}	50	s	30–80	7.5
14	Migrating behavior max. random angle	$\gamma_{migr,r,max}$	40	°/s	10–60	7.5
15	Migrating behavior wall detection range[4]	r_{wall}	4.0	m	2.8–5.8	7.5
16	Migrating behavior wall distance angle	$\gamma_{migr,wall}$	20	°/s	4–44	7.5
16	Migrating behavior velocity angle	$\gamma_{migr,U}$	40	°/s	10–60	7.5
16	Migrating behavior $\|a\|$/TKE angle	$\gamma_{migr,a/k}$	40	°/s	10–60	7.5
17	Migrating $\|a\|$/velocity threshold factor	$k_{a/U}$	1.01	-	1.00–1.05	7.5
18	Initialization “left fish”/“total fish”	L/n	33	%	13–63	5
19	Initialization “slow fish left”/“left fish”	sL/L	38	%	18–68	5
20	Initialization “slow fish right”/“right fish”	sR/R	44	%	24–74	5

For comparison, www.troutandsalmon.com (2020) estimates 10 ft (3 m) as maximum freshwater visibility. For transfer to real fishways, this assumption would need examination of natural light and actual water conditions. Note that other senses like tactile or the lateral line could mediate equivalent information under differing conditions.

7.2 chooseBehavior

The submodel *chooseBehavior* selects one of three horizontal movement behaviors depending on the relation between the state variables motivation M and fatigue F. The submodel sets the behavior variable B to either

$$B = \begin{cases} 1 \quad \text{if} & M_{avg} > F_{avg} + \Delta_{hold} \quad \text{(Migrating)} \\ 2 \quad \text{if} \quad F_{avg} - \Delta_{hold} \leq & M_{avg} \leq F_{avg} + \Delta_{hold} \quad \text{(Holding)} \\ 3 \quad \text{if} \quad F_{avg} - \Delta_{hold} > & M_{avg} \quad \text{(Drifting)} \end{cases} \tag{1}$$

using the holding behavior extent parameter, Δ_{hold} (Table 3). Computation of the time-averaged values M_{avg} and F_{avg} is described in separate submodels.

[1] $n = 66$, mean±SD $= 0.27 \pm 0.04$ m

[2] Castro-Santos et al. (2013): Most individuals swam “well below” the optimal value of $U_g = 5.65$ BL/s

[3] Castro-Santos et al. (2013)

[4] www.troutandsalmon.com (2020) estimates 10 ft (3 m) as typical max. visibility in freshwater

Rationale: The *chooseBehavior* submodel balances driving and retaining factors on a conceptionally high level. It leaves theory development and application to the (arbitrarily complex) submodels for motivation and fatigue, which compute internal states of model fish depending on ambient influence and individual traits. The submodels are described in the following.

7.3 Motivation

Motivation increases or decreases depending on whether the fish holds or moves. First, the fish acclimatizes to its current position, $\vec{s}$, over time. A standard memory function (exponential moving average) is used to link preceding positions to the current position with decaying impact. The acclimatized position, $\vec{s}_{avg}$, is computed as

$$\vec{s}^{\,n}_{avg} = (1 - m_s)\vec{s}^{\,n} + m_s\vec{s}^{\,n-1}_{avg} \tag{2}$$

where m_s $[-]$ is the constant spot memory coefficient (Table 3) and a n superscript denotes the current time step.

While the current fish position is within a relative radius around its acclimatized position, $r_{sameSpot}$ $[-]$, the cumulated time at a spot, $t_{sameSpot}$ [s], is increased by the time step, Δt. If the current position is outside the radius, $t_{sameSpot}$ is reset:

$$t^n_{sameSpot} = t^{n-1}_{sameSpot} + \Delta t \qquad \text{if} \quad \sqrt{(\vec{s} - \vec{s}_{avg})^2} < r_{sameSpot} \cdot \text{BL} \tag{3}$$

$$t^n_{sameSpot} = 0 \qquad \text{if} \quad \sqrt{(\vec{s} - \vec{s}_{avg})^2} \geq r_{sameSpot} \cdot \text{BL} \tag{4}$$

$t_{sameSpot}$ is also reset to $t_{sameSpot} = 0$ after triggering the anti-stuck reaction (see submodel *migrating*). It is used to calculate instantaneous motivation as:

$$M = \frac{1}{k_M} t_{sameSpot} \ [-] \tag{5}$$

$$k_M = \begin{cases} k_{M,f} & \text{if} \quad M^* = \text{“}fast\text{”} \\ k_{M,s} & \text{if} \quad M^* = \text{“}slow\text{”} \end{cases} \tag{6}$$

using motivation denominator k_M [s] and motivatability, M^*. k_M represents the maximum time needed at a given spot to reach maximum instant motivation. M takes values in $[0, 1]$, which is ensured by a ceil limit. The minimum value can be interpreted as “no need to move to new upstream locations”, e.g. just after the fish has entered new territory. The maximum value can be interpreted as the “most urgent need to move to a new upstream location”, e.g. after spatial stagnation for a while.

Finally, motivation is used in the model in time-averaged form, M_{avg}. It is transported through time using another memory function:

$$M^n_{avg} = (1 - m_M)M^n + m_M M^{n-1}_{avg} \tag{7}$$

where m_M $[-]$ is the motivation memory coefficient.

Rationale: Motivation measures the strength of the fish's short-term impetus to migrate upstream and generally fuels upstream movement in the model. The long-term impetus for migration results from general fitness advantages of changing location, such as reaching spawning habitats, greater food availability, and finding new mating partners. However, the laboratory test setup does not allow for conclusions on the long-term impetus. Instead, we use the short-term impetus, which should have the same general tendency, but could be weaker under laboratory conditions, where observation time is limited.

By integrating position memory, motivation is linked to spatial progress. It can be imagined as impatience, which results from low progress despite the (assumed) basic desire for upstream migration. The two categories of fast- and slow-to-motivate fish are a simplification of individual differences in motivation rate. This assumption simplifies development and interpretation of the results.

The position timer for $t_{sameSpot}$ presumes existing orientation over ground, e.g. by vision or previous tactile contact to a boundary. Wall detection range can be limited, e.g. for real-world applications with reduced vision, by changing r_{wall}.

7.4 Fatigue

Fatigue is calculated in dependency of the fish's swim speed relative to the flow, U_s [BL/s], from the previous time step. From the instantaneous value, an average is computed, which is used for the behavioral decision:

$$F^n = \frac{1}{k_F} U_s^{n-1} \quad [-] \tag{8}$$

$$F^n_{avg} = (1 - m_F) F^n + m_F F^{n-1}_{avg} \tag{9}$$

$$m_F = \begin{cases} m_{F,d} & \text{if} \quad F^n \leq F^{n-1}_{avg} \\ m_{F,i} & \text{if} \quad F^n > F^{n-1}_{avg} \end{cases} \tag{10}$$

where n superscript is the current time step and m_F is the fatigue memory coefficient. F is rendered dimensionless by the fatigue denominator k_F [BL/s], which marks the velocity at which full fatigue occurs. U_s units are converted as follows, where BL is body length and $\mathrm{BL}_{trout} = 0.27$ m.

$$U_s \left[\frac{\mathrm{BL}}{\mathrm{s}}\right] = U_s \left[\frac{\mathrm{m}}{\mathrm{s}}\right] \, / \, \mathrm{BL}_{trout} \left[\frac{\mathrm{m}}{\mathrm{BL}}\right]$$

F takes values in $[0, 1]$, which is ensured by a ceil limit. The minimum and maximum fatigue values can be described in words as "hold this speed for unlimited time, feeling perfectly well" and "full exhaustion, try to avoid this state", respectively. Exhaustion was not observed for brown trout in the flume study as the water velocity was moderate for them ($U_m \approx 0.67$ m/s ≈ 2.5 BL/s).

Rationale: Fatigue is an inverse measure of the capability to perform work (e.g., swimming in prolonged or burst mode) and generally slows or even stops upstream migration in the model. Fatigue covers traits such as metabolic cost, body fat reserves, oxygen concentration in the white muscles, individual strength, and injuries. In principal, it could be related to flow velocity via speed-fatigue diagrams (Castro-Santos 2005; Castro-Santos et al. 2013) or model equations (Ebel 2014). Here, a simpler approach is chosen which accounts for temporal aspects indirectly, by memory influence. As suggested by Liao (2007), location preference in combination with information about the flow (e.g. U_m) can serve as a first approximation for metabolic cost in the absence of direct information about energy consumption, e.g. tail-beat frequency or oxygen consumption. The fatigue denominator, k_F, defining maximum swim speed for brown trout, is chosen following Castro-Santos et al. (2013).

From the motivation and fatigue average values, one of the three horizontal behavioral rules/submodels for migrating, holding, and drifting is chosen. An additional submodel steers vertical behavior. They are defined as follows.

7.5 Migrating

All four behavioral submodels produce a swim speed magnitude, U_s, and absolute horizontal and vertical swim angles, γ_s and β_s, which are transformed into the (volitional) swim vector, $\vec{U}_s$, afterwards. Model parameters and their initial values are also listed in Table 3.

Migrating behavior is defined in the model as swimming against the flow vector, faster than flow velocity magnitude. Swim speed magnitude and angles are fixed relative to the flow at

$$U_s = U_m + U_g \cdot \mathrm{BL} \quad [\mathrm{m/s}] \tag{11}$$

$$\gamma_s = \gamma_m - 180° \tag{12}$$

$$\beta_s = -\beta_m \tag{13}$$

where U_m $[\frac{\mathrm{m}}{\mathrm{s}}]$ is mean flow velocity magnitude, U_g $[\frac{\mathrm{BL}}{\mathrm{s}}]$ is fixed fish groundspeed magnitude, BL is fish body length, γ_m is mean flow horizontal angle, and β_m is mean flow vertical angle (see Table 2). The horizontal angle, γ_s, is adjusted against the flow direction (positive rheotaxis). It is converted from semicircle to full circle dimensions and bounded in $[0°, 360°)$, see Table 1.

Next, γ_s is modified depending on the active stimulus:

- Baseline: No further adjustment, continue with wall avoidance and random angle components below.
- Velocity: γ_s is adjusted by $\gamma_{migr,U} \cdot \Delta t$ to the side with smaller velocity magnitude, U_m. If velocity change towards the front is smaller than change towards both sides by the factor $k_{a/U}$, no adjustment is executed.
- Acceleration: γ_s is adjusted by $\gamma_{migr,a/k} \cdot \Delta t$ to the side with greater acceleration magnitude, $|a|$. If acceleration change towards the front is smaller than change towards both sides by the factor $k_{a/U}$, no adjustment is executed.
- TKE: γ_s is adjusted by $\gamma_{migr,a/k} \cdot \Delta t$ to the side with the smaller absolute k difference. If the front difference is smaller than both side differences, no adjustment is executed.
- Wall distance: γ_s is adjusted by $\gamma_{migr,wall} \cdot \Delta t$ to the side with the smaller wall distance within wall detection range, r_{wall}. In the case of equal distances (at machine epsilon precision), no adjustment is performed.

Side values are gauged at the sensory ovoid points SP4 and SP5, front values are gauged at SP2, and differences are computed towards SP1 (fish center). Wall distance is measured in extension of the side sensory points.

Next, wall avoidance behavior checks if the fish center is very close to a wall. If at least one of either SP4 or SP5 is originally placed outside the domain (wall distance $< O_Y = 0.0675$ m before ovoid correction), γ_s is reset adverse to the flow vector, which usually follows the boundary contour, except at the (permeable) screen.

Finally, random influence is added. γ_s is added a random value between $\pm\gamma_{migr,r,max}\Delta t$:

$$r \sim U[0,1] \tag{14}$$

$$\gamma_s = \gamma_s + r(\pm\gamma_{migr,r,max})\Delta t \tag{15}$$

where r is a pseudo-random number drawn from an uniform distribution, U. If SP4 or SP5 is outside the domain, only the opposite side is enabled for random influence by halving the maximum angle. If both sensory points are outside the domain, random influence is turned off.

After being stuck in a place for more than t_{stuck}, the fish selects its swim angles randomly. As vertical blockage is more rare, the vertical swim angle is adjusted only in half of the cases:

$$r \sim U[0,1] \tag{16}$$

$$\gamma_s = r \cdot 359.9° \quad \text{if} \quad (t_{sameSpot} > t_{stuck}) \tag{17}$$

$$\beta_s = 0° \quad \text{if} \quad (t_{sameSpot} > t_{stuck}) \wedge (r > 0.5) \tag{18}$$

The swim speed magnitude, U_s, remains unchanged.

Rationale: In the case of moving against the flow, this behavior elicits constant movement over ground, which is an estimate for optimal energy use (Castro-Santos 2005). Castro-Santos et al. (2013) found a theoretical optimum groundspeed value of $U_g = 5.65$ BL/s for brown trout in prolonged swimming mode. The U_g value estimated here (Table 3) is less than half this value, as our trout mostly swam in sustained mode and Castro-Santos et al. observed a behavioral component leading to slower-than-optimal swimming in most individuals. For comparison, swim speed was selected to be fixed at 2 BL/s (Smith et al. 2014) and 1 m/s (Gao et al. 2016) in other IBMs.

Making the horizontal swim angle relative to the time step, Δt, was found necessary to reduce time step sensitivity.

The wall avoidance component of the *migrating* behavior reduces the number of unrealistic wall collisions. It has the advantage to work also in confined space. Other approaches used in unconfined space, e.g. changing the swim direction to be orthogonal to the wall (Arenas et al. 2015) or resetting fish if they would leave the domain (Goodwin et al. 2014b), were considered too artificial for our confined flume.

Instead of using a correlated random walk, which is a common choice for forward movement (e.g. Goodwin et al. 2014b), we decided to separate stimulus and random components for orientation. This enables switching them on and off individually to

evaluate their respective influence. Generally, the random component reduces unrealistic path repetition if starting from the same position and covers all behavior aspects which are not modeled explicitly.

The anti-stuck rule prevents fish from getting stuck despite intending to move. It is an artificial workaround to prevent visually unrealistic behavior. For example, getting stuck downstream of the screen could occur when a fish intends to move in flow direction, i.e. through the screen, but is blocked physically.

7.6 Holding

Holding behavior is defined as staying in position by setting the swim speed and angles adverse to the mean flow vector's magnitude and angles:

$$U_s = -U_m \tag{19}$$
$$\gamma_s = \gamma_m - 180° \tag{20}$$
$$\beta_s = -\beta_m \tag{21}$$

γ_s is converted from semicircle to full circle dimensions and bounded in $[0°, 360°)$.

Rationale: Holding behavior can be observed close to obstacles and in tranquilized zones, possibly to recreate from fatigue without losing the accomplished position, as in drifting. It serves as transition behavior between migrating and drifting.

7.7 Drifting

Drifting behavior is defined as mostly passive movement with flow speed. Our model also enables retarded drifting (with little effort) by setting the swim speed, U_s, to a random share of the flow velocity, U_m. The horizontal swim angle γ_s is initially set against the flow direction:

$$r \sim U[0,1] \tag{22}$$
$$U_s = r \cdot U_m \tag{23}$$
$$\gamma'_s = \gamma_m - 180° \tag{24}$$
$$\beta_s = 0° \tag{25}$$

where r is a pseudo-random number drawn from an uniform distribution, U. The swim angle either remains aligned with the flow or is adjusted to either side:

$$r_1 \sim U[0,1] \tag{26}$$
$$r_2 \sim U[0,1] \tag{27}$$
$$\gamma_s = \gamma'_s \quad \text{if} \quad (r_1 \leq k_{drift,s}) \tag{28}$$
$$\gamma_s = \gamma'_s + \gamma_{drift} \quad \text{if} \quad (r_1 > k_{drift,s}) \wedge (r_2 > 0.5) \tag{29}$$
$$\gamma_s = \gamma'_s - \gamma_{drift} \quad \text{if} \quad (r_1 > k_{drift,s}) \wedge (r_2 \leq 0.5) \tag{30}$$

where γ_{drift} is the drift deviation angle and $k_{drift,s}$ is the probability for a straight drift with the flow. γ_s is converted from semicircle to full circle dimensions and bounded in $[0°, 360°)$.

The final movement vector over ground results from the combination of flow velocity, swim speed, and swim angle (Figure 3).

Rationale: This behavioral submodel allows to relocate downstream without or with little energy cost. Retardation of drifting could occur if the fish is willingly using flow to relocate, opposed to drifting from exhaustion. The steering component is also reflected by the ability of choosing a drift angle by this rule. The drift deviation angle is constant to reduce complexity.

7.8 Vertical swimming

In addition to vertical swim angles produced by the preceding submodels, a vertical behavior submodel is used to account for pressure acclimatization. It is similar to the one used in Goodwin et al. (2014a). Hydrostatic pressure p is approximated by the z component of the fish's center position, i.e. its elevation. The z axis points against gravity,

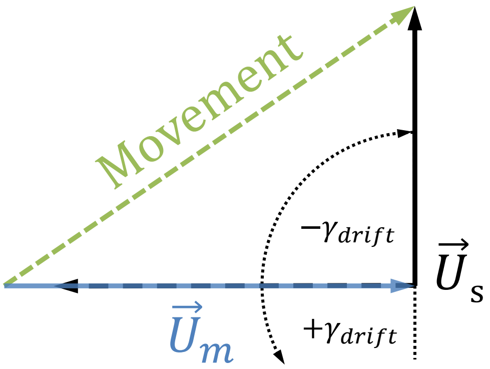

Figure 3: Top view of the movement during drifting behavior. The ground movement vector is the sum of the flow velocity vector, $\vec{U}_m$, and the (volitional) swim vector, $\vec{U}_s$. $\vec{U}_s$ for drifting is effectively computed from the (random) swim speed magnitude for drift, U_s, and the (optional) drift deviation angle, γ_{drift} (which is either 0° or ±90°).

hence elevation is inversely proportional to hydrostatic pressure in the water column. The inverse gradients of pressure and elevation can be ignored, as we solely compare elevations. The acclimatized elevation is transported through time by a memory model:

$$p^n = z \tag{31}$$

$$p^n_{avg} = (1 - m_p)p^n + m_p p^{n-1}_{avg} \tag{32}$$

where m_p is the elevation memory coefficient, and p_{avg} is the acclimatized elevation. The vertical swim angle is adjusted to the opposite direction if the current elevation differs from the acclimatized elevation for more than a certain threshold:

$$\beta_s = -\beta_p \Delta t \quad \text{if} \quad p^n > p^{n-1}_{avg} + k_p \Delta t \tag{33}$$

$$\beta_s = +\beta_p \Delta t \quad \text{if} \quad p^n < p^{n-1}_{avg} - k_p \Delta t \tag{34}$$

Sometimes, local flow disturbances (e.g., strong vertical flow) can lead to a fish's vertical swim angle being negative despite it being close to the flume bottom. To prevent this unrealistic behavior, a minimum vertical swim angle of $\beta_{min} = 5°$ is enforced if the bottom sensory point (SP7) is outside the model domain before sensory ovoid correction.

Rationale: This submodel ensures that sudden changes in pressure, which can be sensed by fish e.g. through their swim bladder, are mitigated immediately. It is kept simple as vertical movement plays a minor role for the observed behavior and model purpose.

7.9 updateFishLocation

Using the result of either migrating, holding, or drifting, and vertical swimming behaviors, the (volitional) swim vector, $\vec{U}_s$, is finally computed from the swim speed magnitude and angles:

$$U_{s,XY} = U_s \cos(\beta_s) \tag{35}$$

$$\left.\begin{aligned} u_s &= \pm U_{s,XY} \cos(\gamma_s) \\ v_s &= \pm U_{s,XY} \sin(\gamma_s) \end{aligned}\right\} \text{signs depend on Cartesian quadrant} \tag{36}$$

$$w_s = U_s \sin(\beta_s) \tag{37}$$

$$\vec{U}_s = \begin{pmatrix} u_s \\ v_s \\ w_s \end{pmatrix} \tag{38}$$

The submodel *updateFishLocation* computes a new three-dimensional position from the (volitional) swim vector, $\vec{U}_s$, the flow vector at the fish center, $\vec{U}_m$, and the time step, Δt, in advance:

$$\vec{s}^{\,n+1} = \vec{s}^{\,n} + (\vec{U}_s + \vec{U}_m) \cdot \Delta t \tag{39}$$

Then, it checks whether the new position would be inside the model domain. If the fish has left through an exit (boundary type "patch" in the model), a counter is increased and the fish is removed. If it has left unphysically through a fixed boundary (type "wall") or crossed the slot wall, the fish is reset to its previous position (*resetPosition* submodel description below).

If the new fish position lies inside the model, but in the air phase, it is forced down in 10 cm-increments until it is in the water phase again. Subsequently, another out-of-bounds check is performed.

Rationale: Fish leaving the domain through screens or walls or crossing the slot wall is physically impossible and needs to be precluded. In the best case, model fish would not choose such paths, but that would require more detailed modeling of boundary sensing, movement predictions, and possibly smaller time steps, which all would contribute little to our purposes. In the present model, the fish is limited to the information at its discrete sensory ovoid points. This information is used to steer in a wall parallel direction (see above, "migrating"), but that is not sufficient to prevent collisions if the fish moves outside its previous sensory ovoid. Hence, numerical safety checks are necessary.

7.10 resetPosition

If one of the checks of *updateFishLocation* fails, the *resetPosition* submodel is called. It resets the fish to its current position:

$$\vec{s}^{\,n+1} = \vec{s}^{\,n} \tag{40}$$

Rationale: The approach chosen has two advantages: It is stable, because the previous position is always valid, and it is computationally cheap, because there is no new position which has to be checked for validity. Its disadvantage, the risk of producing identical results and getting stuck in an infinite loop due to identical environment input, is small, as there is stochasticity included in the movement model. Being reset can be interpreted as an extra holding time step due to geometrical constraints.

References

Arenas, A., M. Politano, L. Weber, and M. Timko (2015). "Analysis of movements and behavior of smolts swimming in hydropower reservoirs". *Ecological Modelling* 312, pp. 292–307. ISSN: 03043800. DOI: 10.1016/j.ecolmodel.2015.05.015 (cit. on p. 11).

Bleckmann, H. and R. Zelick (2009). "Lateral line system of fish". eng. *Integrative zoology* 4 (1), pp. 13–25. DOI: 10.1111/j.1749-4877.2008.00131.x. eprint: 21392273 (cit. on pp. 5, 6).

Castro-Santos, T. (2005). "Optimal swim speeds for traversing velocity barriers: an analysis of volitional high-speed swimming behavior of migratory fishes". *Journal of Experimental Biology* 208 (3), pp. 421–432. ISSN: 00220949. DOI: 10.1242/jeb.01380 (cit. on pp. 4, 10, 11).

Castro-Santos, T., F. J. Sanz-Ronda, J. Ruiz-Legazpi, and B. Jonsson (2013). "Breaking the speed limit — comparative sprinting performance of brook trout (Salvelinus fontinalis) and brown trout (Salmo trutta)". *Canadian Journal of Fisheries and Aquatic Sciences* 70 (2), pp. 280–293. DOI: 10.1139/cjfas-2012-0186 (cit. on pp. 8, 10, 11).

Chapman, B. B., K. Hulthén, D. R. Blomqvist, L.-A. Hansson, J.-Å. Nilsson, J. Brodersen, P. Anders Nilsson, C. Skov, and C. Brönmark (2011). "To boldly go. Individual differences in boldness influence migratory tendency". eng. *Ecology letters* 14 (9), pp. 871–876. DOI: 10.1111/j.1461-0248.2011.01648.x. eprint: 21718420 (cit. on p. 7).

Denton, E. J. and J. A. B. Gray (1988). "Mechanical Factors in the Excitation of the Lateral Lines of Fishes". In: *Sensory biology of aquatic animals.* International Conference on the Sensory Biology of Aquatic Animals (Sarasota, FLA). Ed. by J. Atema, R. R. Fay, A. N. Popper, and W. N. Tavolga. New York, NY: Springer, pp. 595–617. ISBN: 978-1-4612-8317-1. URL: https://link.springer.com/chapter/10.1007/978-1-4612-3714-3_23#citeas (cit. on p. 5).

Ebel, G. (2014). "Modellierung der Schwimmfähigkeit europäischer Fischarten — Zielgrößen für die hydraulische Bemessung von Fischschutzsystemen". German. *Wasserwirtschaft* 104 (7-8), pp. 40–47. ISSN: 0043-0978. DOI: 10.1365/s35147-014-1095-1 (cit. on p. 10).

Gao, Z., H. I. Andersson, H. Dai, F. Jiang, and L. Zhao (2016). "A new Eulerian–Lagrangian agent method to model fish paths in a vertical slot fishway". *Ecological Engineering* 88, pp. 217–225. DOI: 10.1016/j.ecoleng.2015.12.038 (cit. on pp. 4, 7, 11).

Goodwin, R. A., J. M. Nestler, J. J. Anderson, L. J. Weber, and D. P. Loucks (2006). "Forecasting 3-D fish movement behavior using a Eulerian–Lagrangian–agent method (ELAM)". *Ecological Modelling* 192 (1-2), pp. 197–223. ISSN: 03043800. DOI: 10.1016/j.ecolmodel.2005.08.004 (cit. on p. 7).

Goodwin, R. A., M. Politano, J. W. Garvin, J. M. Nestler, D. Hay, J. J. Anderson, L. J. Weber, E. Dimperio, D. L. Smith, and M. Timko (2014a). "Fish navigation of large dams emerges from their modulation of flow field experience". *Proceedings of the National Academy of Sciences* 111 (14), pp. 5277–5282. ISSN: 0027-8424. DOI: 10.1073/pnas.1311874111 (cit. on pp. 2, 4, 7, 12).

— (2014b). "Fish navigation of large dams emerges from their modulation of flow field experience. Supporting Information Appendix (SI Appendix)". 111 (14). URL: http://www.pnas.org/content/suppl/2014/03/19/1311874111.DCSupplemental (cit. on pp. 2, 3, 5, 11).

Grimm, V., U. Berger, F. Bastiansen, S. Eliassen, V. Ginot, J. Giske, J. Goss-Custard, T. Grand, S. K. Heinz, G. Huse, A. Huth, J. U. Jepsen, C. Jørgensen, W. M. Mooij, B. Müller, G. Pe'er, C. Piou, S. F. Railsback, A. M. Robbins, M. M. Robbins, E. Rossmanith, N. Rüger, E. Strand, S. Souissi, R. A. Stillman, R. Vabø, U. Visser, and D. L. DeAngelis (2006). "A standard protocol for describing individual-based and agent-based models". *Ecological Modelling* 198 (1-2), pp. 115–126. ISSN: 03043800. DOI: 10.1016/j.ecolmodel.2006.04.023 (cit. on p. 1).

Grimm, V. and S. F. Railsback (2005). *Individual-based modeling and ecology.* Princeton series in theoretical and computational biology. Princeton: Princeton University Press. 428 pp. ISBN: 1400850622 (cit. on pp. 4, 6).

Grimm, V., S. F. Railsback, C. E. Vincenot, U. Berger, C. Gallagher, D. L. DeAngelis, B. Edmonds, J. Ge, J. Giske, J. Groeneveld, A. S. Johnston, A. Milles, J. Nabe-Nielsen, J. G. Polhill, V. Radchuk, M.-S. Rohwäder, R. A. Stillman, J. C. Thiele, and D. Ayllón (2020). "The ODD Protocol for Describing Agent-Based and Other Simulation Models. A Second Update to Improve Clarity, Replication, and Structural Realism". *Journal of Artificial Societies and Social Simulation* 23 (2). ISSN: 1460-7425. DOI: 10.18564/jasss.4259 (cit. on p. 1).

Kasumyan, A. O. (2011). "Tactile reception and behavior of fish". *Journal of Ichthyology* 51 (11), pp. 1035–1103. ISSN: 0032-9452. DOI: 10.1134/S003294521111004X (cit. on pp. 5, 6).

Kerr, J. R., C. Manes, and P. S. Kemp (2016). "Assessing hydrodynamic space use of brown trout, Salmo trutta, in a complex flow environment: a return to first principles". *Journal*

of Experimental Biology 219 (Pt 21), pp. 3480–3491. ISSN: 00220949. DOI: 10.1242/jeb.134775. eprint: 27591311 (cit. on p. 7).

Liao, J. C. (2007). "A review of fish swimming mechanics and behaviour in altered flows". *Philosophical Transactions of the Royal Society B: Biological Sciences* 362 (1487), pp. 1973–1993. ISSN: 0962-8436. DOI: 10.1098/rstb.2007.2082 (cit. on pp. 4, 5, 10).

Lopez, U., J. Gautrais, I. D. Couzin, and G. Theraulaz (2012). "From behavioural analyses to models of collective motion in fish schools". *Interface focus* 2 (6), pp. 693–707. ISSN: 2042-8898. DOI: 10.1098/rsfs.2012.0033. eprint: 24312723 (cit. on p. 6).

Lucas, M. C. and E. Baras (2001). *Migration of freshwater fishes.* 1st ed. Oxford: Blackwell Science. 420 pp. ISBN: 0-632-05754-8 (cit. on p. 4).

Odling-Smee, L. and V. A. Braithwaite (2003). "The role of learning in fish orientation". *Fish and Fisheries* 4 (3), pp. 235–246 (cit. on p. 5).

Smith, D. L., R. A. Goodwin, Y. Lai, and J. M. Nestler (2014). "Fish path selection and fatigue in complex, continuous velocity fields". In: *10th International Symposium on Ecohydraulics.* 10th International Symposium on Ecohydraulics (Trondheim). Ed. by A. Harby and H. Sundt (cit. on pp. 2, 11).

Wang, H. and H. Chanson (2018). "Modelling upstream fish passage in standard box culverts. Interplay between turbulence, fish kinematics, and energetics". *River Research and Applications* 128 (5), p. 245. ISSN: 1535-1459. DOI: 10.1002/rra.3245 (cit. on p. 5).

Willis, J. (2011). "Modelling swimming aquatic animals in hydrodynamic models". *Ecological Modelling* 222 (23-24), pp. 3869–3887. ISSN: 03043800 (cit. on p. 3).

Zielinski, D. P., V. Voller, and P. W. Sorensen (2018). "A physiologically inspired agent-based approach to model upstream passage of invasive fish at a lock-and-dam". *Ecological Modelling* 382, pp. 18–32. ISSN: 03043800. DOI: 10.1016/j.ecolmodel.2018.05.004 (cit. on p. 4).

Online resources

www.troutandsalmon.com (2020). *What and how trout see underwater.* URL: https://www.troutandsalmon.com/tips-venues-gear/fishing-tips/what-and-how-trout-see-underwater/ (visited on Nov. 16, 2020) (cit. on p. 8).
