# Supplementary material for: Development of behavioral rules for upstream orientation of fish in confined space
Source: PLoS One. 2022 Feb 18;17(2):e0263964. doi: 10.1371/journal.pone.0263964 (PMC8856537; doi:10.1371/journal.pone.0263964)
Supplement: Attachment — Submitted filename: PONE-D-21-08612_reviewer_revAndy.pdf [file pone.0263964.s007.pdf]

PLOS ONE

Development of behavioral rules for upstream orientation of fish in confined space employing wall distance and hydraulic stimuli

--Manuscript Draft--

**Manuscript Number:**	PONE-D-21-08612
**Article Type:**	Research Article
**Full Title:**	Development of behavioral rules for upstream orientation of fish in confined space employing wall distance and hydraulic stimuli
**Short Title:**	Development of behavioral rules for upstream orientation of fish in confined space
**Corresponding Author:**	David C. Gisen<br>Bundesanstalt fur Wasserbau<br>Karlsruhe, GERMANY
**Keywords:**	fish behavior; fishway; individual-based model; computational fluid dynamics; migration
**Abstract:**	Improving the effectiveness of fishways requires a better understanding of fish behavior near hydraulic structures, especially of orientation. One of the most promising approaches to this problem is the use of model behavioral rules.<br>We developed a three-dimensional individual-based model based on observed brown trout (Salmo trutta fario) movement in a laboratory flume. We tested it against two flume setups with different discharges. We used the model to examine which of five behavioral rule versions would best explain upstream trout orientation. Versions differed in the stimulus-response mechanism for swim angle selection. The baseline stimulus was positive rheotaxis. It was supplemented by attraction towards either lower velocity magnitude, constant turbulence kinetic energy, increased flow acceleration, or smaller wall distance.<br>As expected, modeling rheotaxis was central to matching the laboratory observations. In combination with a random component for swim angle selection, the baseline model version was already able to explain large parts of the observed behavior. Mixed results for velocity magnitude, turbulence kinetic energy, and flow acceleration supported the hypothesis that live trout did not orient primarily by means of flow features characterized by these variables, probably because the flow field did not challenge them physically.<br>Significantly improved results suggested that wall distance was the dominant orientation stimulus in our conditions. The absolute root mean square error (RMSE) was small for the best parameter set (RMSE = 9 for setup 1, RMSE = 6 for setup 2). We show that wall distance was most likely perceived visually. For dark or turbid water, also acoustic or tactile reception of walls would be conceivable. We conclude that wall distance estimation should be considered more frequently as an orientation stimulus in confined space. This is true wherever conditions enable wall distance perception, either in individual-based models, laboratory flumes, or in the field.
**Order of Authors:**	David C. Gisen
	Cornelia Schütz
	Roman B. Weichert
**Additional Information:**	
**Question**	**Response**
**Financial Disclosure**<br><br>Enter a financial disclosure statement that describes the sources of funding for the work included in this submission. Review the submission guidelines for detailed requirements. View published research	This work was funded by the German Federal Ministry of Transport and Digital Infrastructure (BMVI, https://www.bmvi.de). The funders had no role in study design, data collection and analysis, decision to publish, or preparation of the manuscript.

Powered by Editorial Manager® and ProduXion Manager® from Aries Systems Corporation

articles from *PLOS ONE* for specific examples.<br><br>This statement is required for submission and **will appear in the published article** if the submission is accepted. Please make sure it is accurate.<br><br>**Unfunded studies**<br>Enter: *The author(s) received no specific funding for this work.*<br><br>**Funded studies**<br>Enter a statement with the following details:<br>• Initials of the authors who received each award<br>• Grant numbers awarded to each author<br>• The full name of each funder<br>• URL of each funder website<br>• Did the sponsors or funders play any role in the study design, data collection and analysis, decision to publish, or preparation of the manuscript?<br>• **NO** - Include this sentence at the end of your statement: *The funders had no role in study design, data collection and analysis, decision to publish, or preparation of the manuscript.*<br>• **YES** - Specify the role(s) played.<br><br>* typeset	
**Competing Interests**<br><br>Use the instructions below to enter a competing interest statement for this submission. On behalf of all authors, disclose any competing interests that could be perceived to bias this work—acknowledging all financial support and any other relevant financial or non-financial competing interests.<br><br>This statement is required for submission and **will appear in the published article** if the submission is accepted. Please make sure it is accurate and that any funding sources listed in your Funding Information later in the submission form are also declared in your Financial Disclosure statement.	The authors have declared that no competing interests exist.

View published research articles from *PLOS ONE* for specific examples.<br><br>**NO authors have competing interests**<br><br>Enter: *The authors have declared that no competing interests exist.*<br><br>**Authors with competing interests**<br><br>Enter competing interest details beginning with this statement:<br><br>*I have read the journal's policy and the authors of this manuscript have the following competing interests: [insert competing interests here]*<br><br>* typeset	
**Ethics Statement**<br><br>Enter an ethics statement for this submission. This statement is required if the study involved:<br><br>• Human participants<br>• Human specimens or tissue<br>• Vertebrate animals or cephalopods<br>• Vertebrate embryos or tissues<br>• Field research<br><br>Write "N/A" if the submission does not require an ethics statement.<br><br>General guidance is provided below. Consult the submission guidelines for detailed instructions. **Make sure that all information entered here is included in the Methods section of the manuscript.**	For model development, we used trout observation data obtained by, but not used within, a study currently under review (Schütz et al. 2021).<br><br>In this study, all care and procedures involving handling and holding fish were conducted as stated and permitted by the district government Karlsruhe (license AZ 35-9185.82/A-6/16).

Powered by Editorial Manager® and ProduXion Manager® from Aries Systems Corporation

**Format for specific study types**<br><br>**Human Subject Research (involving human participants and/or tissue)**<br>• Give the name of the institutional review board or ethics committee that approved the study<br>• Include the approval number and/or a statement indicating approval of this research<br>• Indicate the form of consent obtained (written/oral) or the reason that consent was not obtained (e.g. the data were analyzed anonymously)<br><br>**Animal Research (involving vertebrate animals, embryos or tissues)**<br>• Provide the name of the Institutional Animal Care and Use Committee (IACUC) or other relevant ethics board that reviewed the study protocol, and indicate whether they approved this research or granted a formal waiver of ethical approval<br>• Include an approval number if one was obtained<br>• If the study involved *non-human primates*, add *additional details* about animal welfare and steps taken to ameliorate suffering<br>• If anesthesia, euthanasia, or any kind of animal sacrifice is part of the study, include briefly which substances and/or methods were applied<br><br>**Field Research**<br><br>Include the following details if this study involves the collection of plant, animal, or other materials from a natural setting:<br>• Field permit number<br>• Name of the institution or relevant body that granted permission	
**Data Availability**<br><br>Authors are required to make all data underlying the findings described fully available, without restriction, and from the time of publication. PLOS allows rare exceptions to address legal and ethical concerns. See the PLOS Data Policy and FAQ for detailed information.	Yes - all data are fully available without restriction

A Data Availability Statement describing where the data can be found is required at submission. Your answers to this question constitute the Data Availability Statement and **will be published in the article**, if accepted.<br><br>**Important:** Stating 'data available on request from the author' is not sufficient. If your data are only available upon request, select 'No' for the first question and explain your exceptional situation in the text box.<br><br>Do the authors confirm that all data underlying the findings described in their manuscript are fully available without restriction?	
**Describe where the data may be found in full sentences. If you are copying our sample text, replace any instances of XXX with the appropriate details.**<br><br>• If the data are **held or will be held in a public repository**, include URLs, accession numbers or DOIs. If this information will only be available after acceptance, indicate this by ticking the box below. For example: *All XXX files are available from the XXX database (accession number(s) XXX, XXX.).*<br>• If the data are all contained **within the manuscript and/or Supporting Information files**, enter the following: *All relevant data are within the manuscript and its Supporting Information files.*<br>• If neither of these applies but you are able to provide **details of access elsewhere**, with or without limitations, please do so. For example:<br><br>*Data cannot be shared publicly because of [XXX]. Data are available from the XXX Institutional Data Access / Ethics Committee (contact via XXX) for researchers who meet the criteria for access to confidential data.*<br><br>*The data underlying the results presented in the study are available from (include the name of the third party*	All relevant data are within the manuscript and its Supporting Information files. All model source code files are available from Github, https://github.com/baw-de/ELAM-flume

and contact information or URL).<br>• This text is appropriate if the data are owned by a third party and authors do not have permission to share the data.<br><br>* typeset	
Additional data availability information:	

Manuscript
Click here to access/download;Manuscript;2021-03-16_ELAM-paper-final.docx

Development of behavioral rules for upstream orientation of fish in confined space employing wall distance and hydraulic stimuli

David C. Gisen[1*], Cornelia Schütz[2], Roman B. Weichert[1]

[1] Waterways and environment unit, Bundesanstalt für Wasserbau, Karlsruhe, Germany

[2] Animal ecology unit, Bundesanstalt für Gewässerkunde, Koblenz, Germany

* Corresponding author
E-mail: david.gisen@baw.de (D.C.G.)

Abstract

Improving the effectiveness of fishways requires a better understanding of fish behavior near hydraulic structures, especially of orientation. One of the most promising approaches to this problem is the use of model behavioral rules.

We developed a three-dimensional individual-based model based on observed brown trout (*Salmo trutta fario*) movement in a laboratory flume. We tested it against two flume setups with different discharges. We used the model to examine which of five behavioral rule versions would best explain upstream trout orientation. Versions differed in the stimulus-response mechanism for swim angle selection. The baseline stimulus was positive rheotaxis. It was supplemented by attraction towards either lower velocity magnitude, constant turbulence kinetic energy, increased flow acceleration, or smaller wall distance.

As expected, modeling rheotaxis was central to matching the laboratory observations. In combination with a random component for swim angle selection, the baseline model version was already able to explain large parts of the observed behavior. Mixed results for velocity magnitude, turbulence kinetic energy, and flow acceleration supported the hypothesis that live trout did not orient primarily by means of flow features characterized by these variables, probably because the flow field did not challenge them physically.

Significantly improved results suggested that wall distance was the dominant orientation stimulus in our conditions. The absolute root mean square error (RMSE) was small for the best parameter set (RMSE = 9 for setup 1, RMSE = 6 for setup 2). We show that wall distance was most likely perceived visually. For dark or turbid water, also acoustic or tactile reception of walls would be conceivable. We conclude that wall distance estimation should be considered more frequently as an orientation stimulus in confined space. This is true wherever conditions enable wall distance perception, either in individual-based models, laboratory flumes, or in the field.

1 Introduction

Worldwide, fishways play a key role in efforts to restore upstream-directed fish migration at dams. Widely varying effectiveness of existing projects (Bunt et al. 2012; Bunt et al. 2016) illustrates how challenging their design is, especially for species other than Pacific salmon (Kemp 2016). Improving fishway effectiveness for all species requires a better understanding of fish behavior near hydraulic structures, especially of orientation and navigation (Willis 2011; Kemp 2016; Silva et al. 2018). Orientation can be defined as directional response to local conditions and navigation as movement towards a goal outside the local sensory environment (Willis 2011). One of the most promising approaches to better understand fish behavior is the use of behavioral rules (Silva et al. 2018). Behavioral rules are the logical/algebraical formulation of behavioral hypotheses. They are implemented and tested in individual-based models (IBMs, Grimm and Railsback 2005). Development of behavioral hypotheses for orientation and testing derived rules requires data on movement and stimuli.

Proper selection of stimuli that predict space use is the subject of recent debate (Kerr et al. 2016). The most common hydraulic stimuli are velocity direction, velocity magnitude, and one of the various turbulence variables, as described in the following studies on stimuli response. Their wide differences in features like confinement (tailrace to fishway pool), method (laboratory observation or IBM), and migration direction (upstream or downstream) does only affect the particular response hypothesis, but not stimuli selection in general.

Velocity is defined by direction and magnitude. Velocity direction is a fundamental stimulus in most studies, as rheotaxis is crucial for guiding both upstream and downstream navigation (Arnold 1974; Elder and Coombs 2015). Velocity magnitude is also commonly considered, most prominently for ascent (e.g., Padgett et al. 2020). Since ascent requires energy, fish have developed strategies to reduce their effort (Kerr et al. 2016). One strategy is to avoid zones of higher velocity for moving and holding (low-velocity seeking), as energy demand is proportional to the cube of relative fish velocity (Wang and Chanson 2018). Corresponding behavior was observed in laboratory flumes for longnose dace (Dockery et al. 2017) and brown trout (Kerr et al. 2016). It was modeled in a horizontally unconfined IBM for carp and sturgeon (Zielinski et al. 2018).

Turbulence as a stimulus is usually characterized by means of turbulence kinetic energy (TKE) and/or Reynolds shear stress (τ_{xy}), which represent different components of the specific Reynolds stress tensor. In laboratory studies, adult Iberian barbels avoided areas of high τ_{xy} and were disoriented by eddies of size similar to their body length (BL = 0.15–0.35 m) (Silva et al. 2012). Analysis of the turbulence levels (TKE and τ_{xy}) at chosen locations showed a clear preference for maintaining adapted turbulence levels in dace (Goettel et al. 2015). Brown trout preferred areas of low drag, which was a better explanation than TKE, turbulence intensity, and τ_{xy} in one study (Kerr et al. 2016). In an IBM, two trout paths were best reproduced by avoiding areas of low and high TKE (Gao et al. 2016). In a follow-up model, TKE, velocity magnitude, and strain rate were combined, with the former two having larger influence than the latter on the tracks of three silver carp (Tan et al. 2018).

Advective acceleration was applied several times as an IBM stimulus for guiding juvenile salmon descent (Arenas et al. 2015). It was validated by observations from seven large dams using an Eulerian-Lagrangian-agent method (ELAM, Goodwin et al. 2014). For acceleration use in ascent, a first unvalidated model of steelhead behavior exists (Smith et al. 2014).

Non-hydraulic stimuli for fish orientation, such as visual, acoustic, olfactory, thermal, and magnetic stimuli, are rarely investigated in flows that are comparable to a fishway flow. This is particularly notable for vision, as it is considered important for orientation (Montgomery et al. 2000; Liao 2007). Vision was controlled only in few studies on space use of fish moving against the flow (Liao 2006; Kerr et al. 2016). Studies with similar objectives using vision as explanatory variable were limited to general phototaxis, i.e. attraction or repulsion (Elder and Coombs 2015; Jones et al. 2017). IBMs offer new opportunities to investigate vision in greater detail. For example, it is known that visually determined angle and distance can be used by goldfish to estimate object sizes (Douglas and Hawryshyn 1990).

To find possible behavioral rules for upstream fish orientation and navigation in confined space, e.g. in fishways, we developed a new IBM based on brown trout movement in a laboratory flume. Being ELAM-type, the IBM consists of a computational fluid dynamics (CFD) model, a behavioral model, and the associated software framework. In the work described in this paper, we (1) defined movement patterns from the brown trout data, (2) developed and tested a CFD model to generate stimuli data, (3) developed and tested a behavioral model to reproduce the trout movement patterns, and (4) examined which of five external stimuli (baseline rheotaxis, velocity magnitude, TKE, flow acceleration, and wall distance) would best explain upstream trout orientation and navigation.

2 Methods

2.1 Flume setups

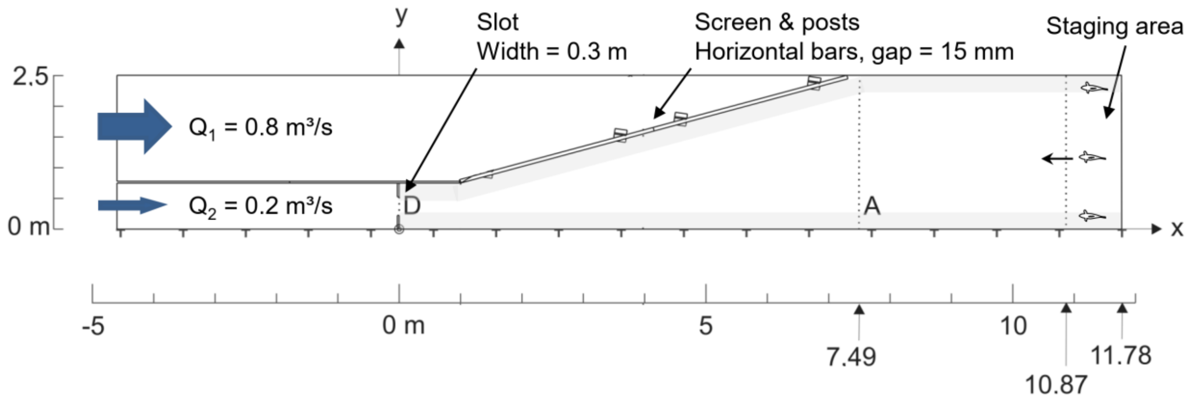

Figure 1: Plan view of flume setup 1. Total length and width = 16.78 m x 2.50 m, water depth = 0.60 m. Flow direction was from left to right. The slot was removed in setup 2 to alter the flow field. Transparent gray areas mark left and right zones (in flow direction) for pattern P1. *A* and *D* mark control lines to filter inactive fish and to define pattern P5.

For developing and testing the behavioral model, we used unpublished brown trout movement data from a previous study (wild *Salmo trutta fario,* n = 66, mean body length ± standard deviation (SD) =

0.27±0.04 m) (Schütz et al. 2021). The study was conducted in a wide indoor flume (Figure 1). The external walls and bottom consisted of smooth acryl glass and metal and the internal walls consisted of coated wood. Water temperature was 16.2-18.2 °C. Water was clear and evenly illuminated. All care and procedures involving handling and holding fish were conducted as stated and permitted by the district government Karlsruhe (license AZ 35-9185.82/A-6/16).

Fish were observed in two setups. The first setup included a jet created by a slot; the second setup had no slot and consequently, no jet. Total discharge was Q = 1.00 m^3/s in both setups. The flume was wide enough to enable distinct lateral movement, but narrow enough to influence behavior through the walls and screen.

Fish were released unmarked in batches of three at the downstream end (staging area in Figure 1). Fish were not re-used to avoid learning effects. Fish positions between x = 0.00 m and x = 9.74 m and visits to the staging area were tracked manually and independently by two biologists. The estimated spatial accuracy was in the order of sub-meters in longitudinal direction and few decimeters in the vertical direction. In the lateral direction, position was recorded as being either close (few decimeters) or far from a wall/the screen. Moreover, it was recorded if fish swam alone or in groups of two or three. Records were attributed to the current test minute. Data were verified qualitatively using video records. Fish which did not cross line A (Figure 1) within 30 min after removing the staging mesh screen were excluded as inactive. A trial was finished either after all three fish passed line D or 60 min after the first crossing of line A. Data points following a passage of line D were also excluded. After filtering, we obtained n = 25 tracks for setup 1 and n = 24 tracks for setup 2.

2.2 Movement patterns

We processed the trout track data using *patterns* in the sense of pattern-oriented modeling (Grimm and Railsback 2012) using MATLAB R2018b. We defined five patterns, P1-P5, which capture the most striking spatial behaviors in the whole domain:

P1, lateral distribution: The flume was divided into a left, middle, and right zone (looking downstream, Figure 1). The dividing lines were located at a lateral distance of Δy = 0.25 m (10 % flume width) to one of either side walls or the screen. Time spent by single fish (i.e., center of mass) was summed for each zone. To account for the differences in total track duration (see Rodríguez et al. 2015), resulting time sums per fish and zone, $t_{zone,i}$, were divided by the particular fish's track duration, $t_{s,i}$, to get relative track fractions, s_{zone}. Finally, s_{zone} was averaged by the fish count, n, to get:

$$\bar{s}_{zone} = \frac{1}{n}\sum_{i=1}^{n}\frac{t_{zone,i}}{t_{s,i}} * 100 \;\; [\%] \qquad \text{(Eq. 1)}$$

where i was a fish iterator. The resulting three lateral zone averages constituted pattern P1. As it constitutes the most direct representation of orientation effects, matching it was a major goal of model development.

P2, vertical distribution: Vertically, the flume was divided into a surface, middle, and bottom zone. The dividing lines were located at a vertical distance of Δz = 0.15 m (25 % water depth) to either the water

surface or flume bottom, respectively. Tracks were averaged like for P1 to obtain three vertical zone average values. Due to the shallow water, we assumed P2 to be of less significance for orientation.

P3, turn area and *P4, few turns*: Turns were defined as changes in the longitudinal movement direction, x, leading to a displacement $\Delta x \geq 2$ BL before the next direction change. About half of the fish in both data sets performed 4 or less turns. To avoid bias, the data set was divided into turning (> 4 turns, P3, n_{turns}) and few-/no-turning fish (≤ 4 turns, P4, $n_{few\text{-}turns}$). For computing P3, the flume was divided longitudinally into three zones of equal length, in which the relative turn shares were computed and averaged by n_{turns} analog to Eq. 1. P4 was defined as $n_{few\text{-}turns}/n$.

P5, arrival rate: The count of fish navigating the total length of the experimental flume area and crossing line D, divided by the count of fish crossing line A, formed P5: $n_D/n \cdot 100$ [%].

For use in discussion, we also calculated the lateral distribution of fish at the start position and the proportion of track time fish spent without other fish nearby (i.e., not shoaling).

2.3 Computational fluid dynamics model

A computational fluid dynamics (CFD) model was employed to calculate the velocity, TKE, and acceleration fields of both setups. We applied the free-surface solver *interFoam* of OpenFOAM 2.3.1 (The OpenFOAM Foundation 2014) to solve the 3D incompressible URANS (unsteady Reynolds-averaged Navier-Stokes) equations.

A hexahedron-dominant unstructured mesh was generated using *snappyHexMesh*. Base resolution was uniform $\Delta_{xyz} = 5$ cm, with local refinement to $\Delta_{xyz} = 1.25$ cm around the slot and screen posts (Figure 1). Horizontal screen bars were omitted, as their influence on the velocity and turbulence field was negligible with respect to behavioral data accuracy (Gisen 2018). Total cell count for setup 1 was 848,694.

Inlet flow rates and outlet fixed water level were set to the laboratory values. For turbulence closure we chose the k-ω-SST model. At the inlet, TKE ($\triangleq k$) was set to $k = 0.001$ m^2/s^2 and the specific rate of TKE dissipation was set to $\omega = 1$ Hz. No-slip conditions (velocity vector $U = 0$) and a small sand-equivalent roughness coefficient ($k_S = 1 \cdot 10^{-5}$ m) were set at the walls and bottom.

The fields of velocity U and water/air distribution α_{water} were initialized with a largely converged solution and run for 80 s of simulated time. U was averaged over the final 20 s to obtain the steady flow velocity vector field $\vec{U}_m$. $\vec{U}_m$ can be expressed either as three-component vector (u,v,w) or as magnitude U_m along with its horizontal and vertical angles γ_m and β_m.

TKE is defined as half the sum of the velocity components' variances:

$$TKE = \frac{1}{2}\overline{U_i'U_i'} = \frac{1}{2}\left(\overline{u'^2} + \overline{v'^2} + \overline{w'^2}\right) \quad \left[\frac{m^2}{s^2} = \frac{J}{kg}\right] \qquad \text{(Eq. 2)}$$

using Reynolds decomposition, $U_i = \overline{U_i} + U_i'$.

Advective acceleration a is caused by changes in the flume cross-section and by friction. It is computed from U_m, i.e. temporal acceleration is absent. In 3D Cartesian coordinates, advective acceleration is defined as

$$a = \begin{pmatrix} a_x \\ a_y \\ a_z \end{pmatrix} = (U^T \, grad \, U)^T = \left(\begin{pmatrix} u & v & w \end{pmatrix} \begin{pmatrix} \frac{\partial u}{\partial x} & \frac{\partial v}{\partial x} & \frac{\partial w}{\partial x} \\ \frac{\partial u}{\partial y} & \frac{\partial v}{\partial y} & \frac{\partial w}{\partial y} \\ \frac{\partial u}{\partial z} & \frac{\partial v}{\partial z} & \frac{\partial w}{\partial z} \end{pmatrix} \right)^T \quad \left[\frac{m}{s^2}\right] \qquad \text{(Eq. 3)}$$

where the superscript ^{T} denotes transposition from column to row vector. Its magnitude $|a|$, U_m, and TKE were used as hydraulic stimuli variables in the behavioral model.

2.4 Behavioral model description

A complete, detailed model description, following the ODD (overview, design concepts, details) protocol (Grimm et al. 2006; Grimm et al. 2020) can be found in appendix S4. Here, we provide a summary.

The overall purpose of our model was to find possible stimulus-response relations (behavioral rules) for fish to enable predictions of fishway attraction and passage efficiency in the long-term. Specifically, we addressed the following question: How well do different stimuli explain orientation and navigation of upstream moving brown trout? Orientation was modeled directly as stimulus-guided swim angle selection, and navigation was expected to emerge indirectly as a result of the whole behavioral model.

The entities of the model were fish and mesh cells. Fish were represented as spatially explicit points in 3D space. Their key state variables were position, motivation, and fatigue. Fish movement was modeled kinematically, i.e. there was no influence of fish on the flow field. Key state variables of the mesh cells were the cell label number and the hydraulic variables $|a|$, U_m, TKE, and α_{water}. Spatially, the behavior model covered the fish-accessible areas of the CFD model (i.e. without the air phase and without areas upstream of the screen). The spatial resolution was that of the CFD model, i.e. 5 cm or smaller. The temporal extent was 60 min of clock time, equal to the maximum laboratory trial duration, divided into constant time steps of $\Delta t = 0.5$ s.

Each model run used $n_{model} = 100$ fish of BL = 0.27 m (as measured), each being assigned to either a "fast" or "slow" category. In this way we tried to model individual traits which could influence migratory tendencies, such as being bold or shy (Chapman et al. 2011) or being physically strong or weak. Model fish were positioned only in the left and right zones of the staging area (Figure 1) as per observations. Their initial distribution was steered by a parameter.

The following process was repeated every time step for every fish to determine a new position: First, a sensory ovoid consisting of seven points around the fish center was determined (Goodwin et al. 2014). Hydraulic variables were interpolated to these points. They were chosen approximately at the skin/water interface, where e.g. pressure fluctuations are mediated by means of the lateral line system (Bleckmann and Zelick 2009). Wall distances were computed towards the front and sides. Next, motivation (to swim

upstream) was increased proportionally to time as long as the fish was not moving. Fatigue was increased proportionally to local flow velocity. From the balance of time-averaged motivation and fatigue, one of three horizontal behavioral rules was selected (Figure 2):

- migrating (moving upstream),
- holding (maintaining position),
- drifting (moving downstream while allowing retardation and/or inclination towards the flow).

This approach allowed model fish to drift, rest, and repeat attempts at critical passages (e.g. slots), a behavior which we consider crucial for modeling fish migration through fishways. Its success will be measured foremost by patterns P3 and P4.

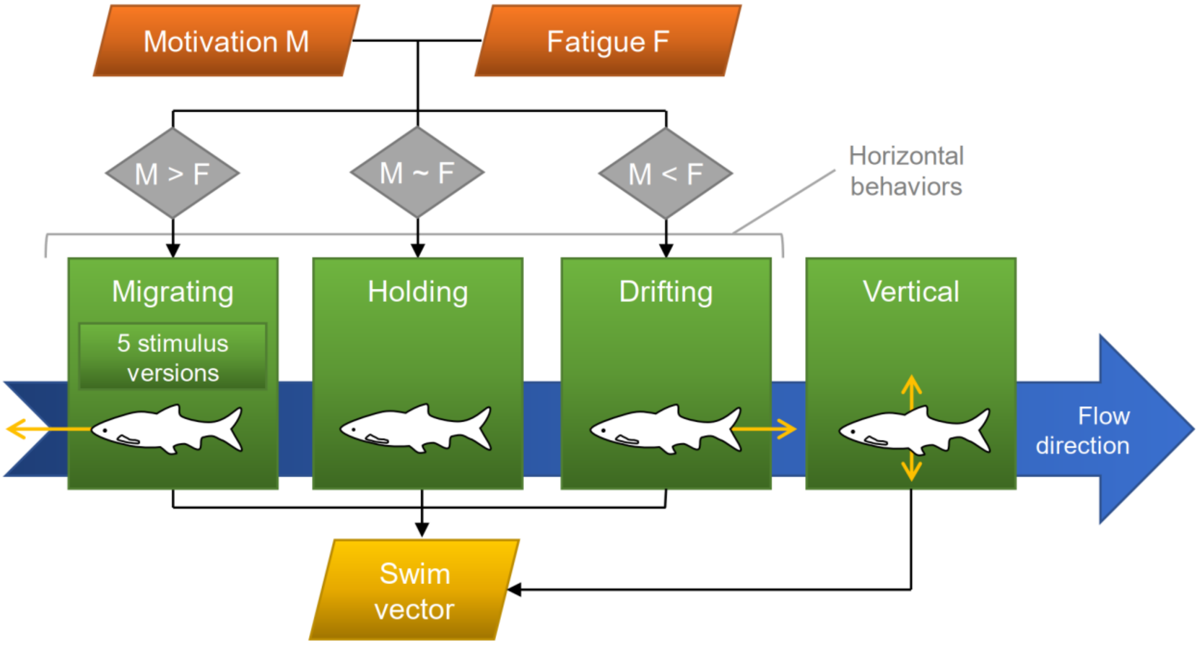

Figure 2: The process of computing the swim vector: Based on the balance between motivation and fatigue, a horizontal behavioral rule is selected. *Migrating* is the key behavioral rule for which five versions are contrasted. *Drifting* behavior includes swimming against, but slower than the flow. In combination with the vertical behavioral rule, the final swim vector is determined.

Migrating was the key behavioral rule. In its baseline version it only depended on the flow direction. It sets the horizontal and vertical swim angles against the flow direction, as we judged rheotaxis to be a fundamental behavior in fish that is always active (e.g. in Mexican tetra, Elder and Coombs 2015). To also capture smaller, unpredictable behavior variations, a limited random angle was added to or subtracted from the horizontal swim angle. Migrating was expected to have the most direct effect on pattern P1.

Our guiding question ("how well do stimuli explain upstream orientation") was addressed by adding four options to the baseline migrating behavioral rule. Each was based on a different stimulus for orientation. That is, the horizontal swim angle could be adjusted in either one of five different stimulus versions:

- Baseline: Against flow direction & towards random side by random magnitude as described above;
- Velocity: Baseline & towards side of lower velocity magnitude (low-velocity seeking);
- TKE: Baseline & towards front or side with the smallest TKE difference towards the fish center;
- Advective acceleration: Baseline & towards side of higher $|a|$ magnitude;
- Wall distance (as a general proxy for non-hydraulic stimuli): Baseline & towards side of smaller wall distance.

An independent vertical behavioral rule was always active. It limited vertical movement using an elevation difference (Goodwin et al. 2014) and was primarily gauged by pattern P2.

The output of the process was a 3D swim vector for each fish and time step. The new position was determined from this swim vector, the flow vector at the fish's position, and the time step width. Overall navigational success was measured by P5.

2.4.1 Code and speed

The code of the behavior model and its software framework can be found in appendix S5. The software framework was based on the Fortran 90 code for downstream migrating smolts used in Goodwin et al. (2014). It accounted for e.g. variable storage, sensory point creation, vector transformation from/to Cartesian coordinates, and pseudo-random number creation. We modified it to work with unstructured, polyhedral meshes in OpenFOAM 4.1 using C++. Computation time varied depending on how fast fish exited the domain. A typical runtime for a single model run with n_{model} = 100 fish was about 85 s, using 8.1 GB RAM for up to 7200 time steps on one core of an Intel E5-2660-v3 ("Haswell") CPU.

2.5 Model function test

Besides behavioral rules, parameters (i.e., variables which do not change during a model run, but can change between model runs) are the second main component of a behavioral model. Our methods for testing their influence are described in this section before returning to our actual purpose of testing different stimuli in section 2.6.

2.5.1 Evaluation metric RMSE

To rate the quality of each model run, we computed pattern values P1-P5 in analogy to the laboratory patterns from n_{model} = 100 fish. As evaluation metric, we computed a weighted root-mean-square error (RMSE) between all model and laboratory pattern mean values (Table 1 below) for each model run as

$$RMSE_{model-run} = \sqrt{a_1 \Delta P_1 + a_2 \Delta P_2 + a_3 \Delta P_3 + a_4 \Delta P_4 + a_5 \Delta P_5} \quad [-] \qquad \text{(Eq. 4)}$$

Average of pattern P1-P3: $\Delta P_i = \frac{1}{3} \sum_j \left(P_{i,j,model} - P_{i,j,lab}\right)^2$

Pattern P4-P5: $\Delta P_i = \left(P_{i,model} - P_{i,lab}\right)^2$

where a was a pattern weight factor chosen per pattern importance (a_1 = 0.3, a_2 = 0.1, $a_{3\text{-}5}$ = 0.2), i = 1..5 was a pattern iterator, and j = 1..3 was a pattern value iterator. As P1-P3 consisted of three pattern values each, these were averaged. A value of RMSE = 0 implies perfect agreement between model and laboratory results. We chose RMSE as it is both simple and penalizes large differences, which makes it useful for filtering outliers and achieving an overall good agreement.

The different weights of patterns P1 and P2 reflected their estimated importance for modeling orientation (section 2.2). We also tested equal weighting per pattern average value and equal weighting per pattern value, but obtained negligible effects on the stimulus version ranking. We focused on comparing the mean instead of SD values to reduce the complexity of model development, accepting that SD agreement cannot be evaluated.

2.5.2 Random seed sensitivity

The behavioral rules used pseudo-random numbers which were generated from a random seed number fixed for each model run. For identical seeds, results were identical. To minimize and to quantify the influence of the random seed choice, we tested each parameter set with N_r = 10 different seeds. Then, we averaged the resulting $\text{RMSE}_{\text{model-run}}$ values to obtain one RMSE value per parameter set. As seeds, we obtained ten true random numbers from www.random.org in the range from 1-999: 656, 36, 849, 934, 679, 758, 743, 392, 655, 171.

2.5.3 Parameter set generation

In total, the model had 7 fixed and D = 20 variable parameters (Table 2 in results and Table 3 in appendix S4, p. 8). The latter could be split into D_1 = 15 parameters for general movement, D_2 = 3 parameters for initialization, and D_3 = 1-2 parameters depending on the chosen stimulus version.

For sensitivity testing and stimulus version testing, we needed to generate a number of parameter sets with systematic variation in their parameter values. To ensure comparability and to save computational effort, we first included all parameters of the five stimulus versions and subsequently filtered the resulting parameter sets as required (Figure 3).

Values of an initial default parameter set were established by trial-and-error. For variation, we chose another 5 values in equal distances around the default value to test a total of p = 6 values for each parameter. The default values were either at the 3rd or 4th place of the range (appendix S4).

The full parameter space would comprise p^D parameter sets. We sampled it using the revised Morris method (Campolongo et al. 2007, r = 1000 random trajectories, T = 50 optimum trajectories) implemented in Python 3.7.4 with SALib 1.3.8. We obtained $n_{sets,base} = (D+1) \cdot T$ = 1050 systematically varying base parameter sets consisting of D parameter values each.

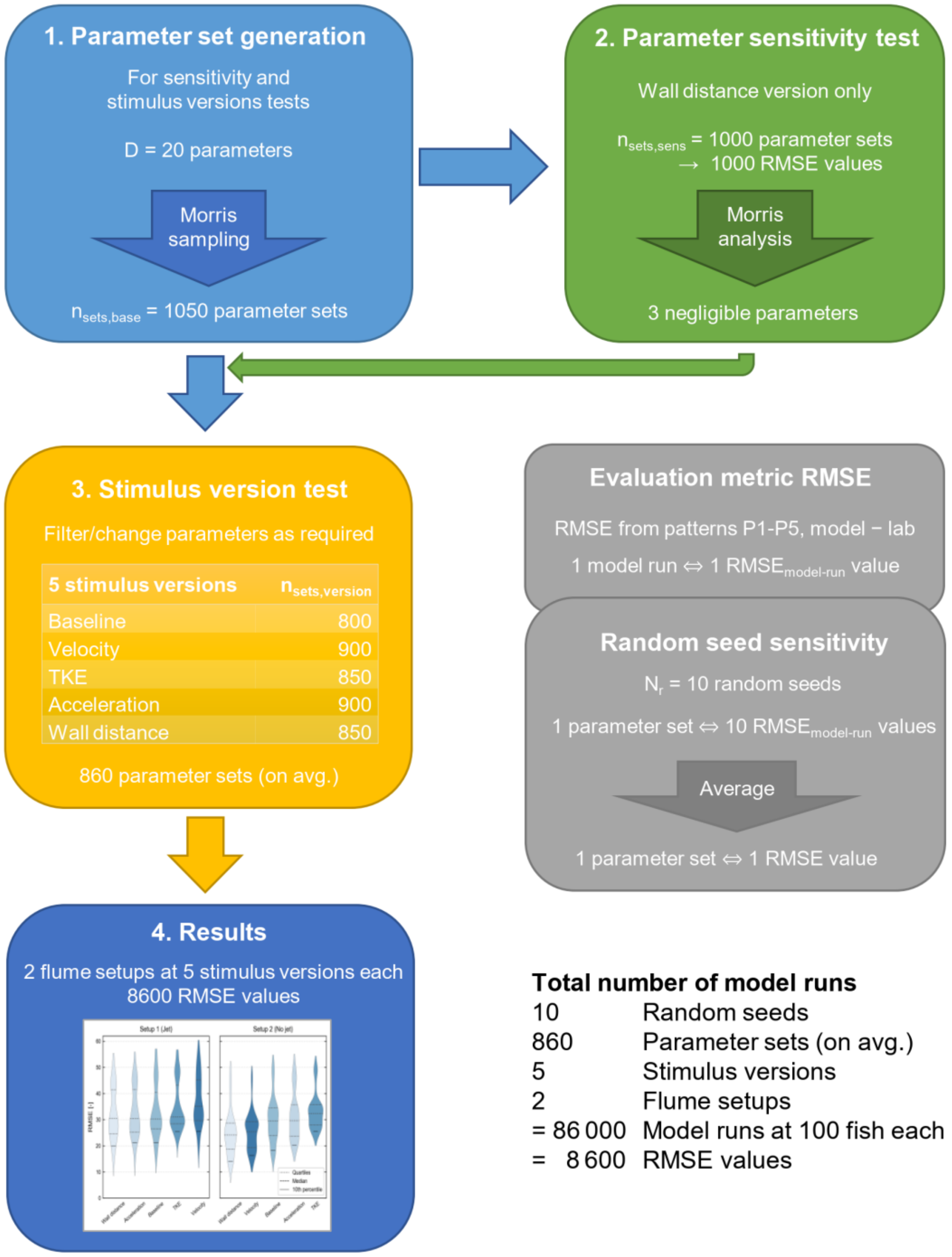

Figure 3: The process of testing our behavioral model starts with the generation of varying parameter sets, which are used for testing parameter sensitivity and contrasting stimulus versions in the following. Each box corresponds to one text section.

2.5.4 Parameter sensitivity test

To identify parameters of strong and negligible influence, we analyzed parameter sensitivity. As stimulus version for this investigation, we arbitrarily chose the wall distance in both flume setups. Thus, the acceleration/TKE stimulus threshold parameter was not required here. This reduced the number of parameter sets by T variations to $n_{sets,sens}$ = 1000 parameter sets. As described above, for each parameter set we ran the model with N_r random seeds and averaged the resulting $RMSE_{model\text{-}run}$ values.

Results were analyzed by means of revised Morris screening (Campolongo et al. 2007; Thiele et al. 2014). This method uses the effect of single parameter variations on the averaged RMSE to determine an

influence measure, $\mu*$, and an interaction measure, σ_M, for each parameter. We used $\mu*$ for ranking parameters by influence. For further interpretation, the values of the measures needed to be classified as "high" or "low". This is usually achieved graphically (e.g., Campolongo et al. 2007). We took a quantitative approach through normalizing $\mu*$ and σ_M by their respective maximum values and, then, defining limits in dependency of gaps in our results. We neglected three parameters with "low" influence (see results) in the subsequent stimulus version tests.

2.6 Stimulus version test

2.6.1 Parameter influence

We focused on testing external stimuli, as they can be readily predicted. This is a necessary requirement for our long-term goal of improving fishway performance. To find the stimulus which best explained upstream orientation, we tested all five versions of the migrating behavioral rule in both setups. All other behavioral rules of the model (including behavior selection, holding, drifting, and vertical behavior) were kept constant (for a protocol, see Grimm and Railsback 2012). The latter were tested implicitly by means of the overall result, to which they contributed.

To eliminate the risk of favoring a stimulus by a certain calibration/parameter set ("overfitting"), we applied a large number of identical parameter sets to each of the stimulus versions. They were generated from the base parameter sets by filtering the negligible parameters identified in sensitivity analysis. Slight differences resulted from three stimulus-specific parameters:

- the wall detection range did only apply to the wall distance stimulus;
- the threshold factor did only apply to the velocity and TKE stimuli;
- the default angle for the wall distance stimulus was half the default angle of the velocity, TKE, and acceleration stimuli.

This procedure yielded an average of $n_{sets,avg}$ = 860 parameter sets per stimulus version (range 800–900, Figure 3). For each parameter set, we ran the model with N_r random seeds and averaged the resulting $RMSE_{model\text{-}run}$ values to obtain one RMSE value. From the combination of parameter sets ($n_{sets,avg}$ = 860), flume setups (n_{setups} = 2), and stimulus versions ($n_{stimuli}$ = 5), we received a total of 8600 RMSE values (Figure 3).

2.6.2 Ranking

Since sampling of the parameter sets is a random process and not optimized towards better performance results, it includes many unfavorable parameter sets that are not suitable to assess stimuli performance. Thus, a selection of the best model runs/RMSE values was necessary to find out if the different stimulus versions performed equally good or differed from each other. Based on the frequency distribution, we sought a threshold where a clear rejuvenation with regard to better values was visible, while still enough RMSE values for a statistical valid comparison remained. We opted for the 10th percentile of RMSE values, which was then used to rank the stimulus versions.

We treated both setups separately, as setup 1 was more demanding on the model due to the more heterogeneous flow field and the slot geometry.

To evaluate if differences between the stimulus versions were statistically significant, we computed the *p*-values (Kruskal-Wallis test with post-hoc Nemenyi test) between the RMSE results (cut-off above 10th percentile) of the stimulus versions separately for both setups using SciPy 1.0.1 with scikit-posthocs 0.6.6.

Finally, to facilitate vivid understanding and discussion, we classified the versions qualitatively. The best version per setup was classified as “good”, which was backed by comparing the RMSE of its best parameter set to reference values. Versions differing significantly ($p < \alpha = 0.01$) from the best version were classified as “moderate” or “poor” under consideration of the 10th percentile. Versions not differing significantly were classified as “good”.

3 Results

3.1 Movement patterns

Analysis of the movement patterns provided several insights into observed trout behavior (Table 1). Pattern P1 indicates that brown trout spent the major part of trial time close to the lateral walls and screen, i.e. in a rather small share of the flume width. P2 shows that fish swam close to the bottom almost exclusively. P3-P5 are less distinct. Large SD values indicate wide individual differences in observed behavior for P1 and P3.

Brown trout start positions were distributed to the P1 left/middle/right zones 68 % - 4 % - 28 % (setup 1) and 46 % - 13 % - 42 % (setup 2). They spent an average of about 60 % of track duration without other fish nearby.

Table 1: Mean and standard deviation (SD) of brown trout patterns in the two flume setups.

**Pattern**	**Flume section**		**Setup 1 (jet)**			**Setup 2 (no jet)**		
	Name (facing downstream)	Extent (m)	*n*	mean (%)	SD (%)	*n*	mean (%)	SD (%)
P1 Lateral distribution	Left	0.25	25	33	±37	24	38	±39
	Middle	2.00	25	10	±12	24	6	±10
	Right	0.25	25	57	±38	24	56	±40
P2 Vertical distribution	Surface	0.15	25	1	±2	24	4	±17
	Middle	0.30	25	1	±2	24	0	±1
	Bottom	0.15	25	99	±3	24	96	±17
P3 Turn area	Upstream	3.25	9	24	±19	12	27	±17
	Middle	3.25	9	29	±17	12	40	±16
	Downstream	3.25	9	47	±16	12	33	±26
P4 Few turns	-	-	15	60	-	12	50	-
P5 n_D/n	-	-	25	84	-	24	79	-

Values rounded to the closest integer. n_D and n, number of fish at lines D and A.

3.2 Computational fluid dynamics model

CFD model results were in good agreement with acoustic Doppler velocimeter measurements from the laboratory (Gisen 2018). Advection dominated the flow field; roughness influence was limited to a few centimeters in both setups (Figure 4). Absence of the jet in setup 2 considerably reduced mean velocity U_m, acceleration magnitude, and TKE on the right-hand flume side (facing downstream). The flow field of both setups was asymmetric in lateral direction. The bulk flow velocity was U_{bulk} = Q/(width*water depth) ≈ 0.67 m/s. In large parts of the jet region of setup 1, flow velocity was about U_m ≈ 0.8 m/s.

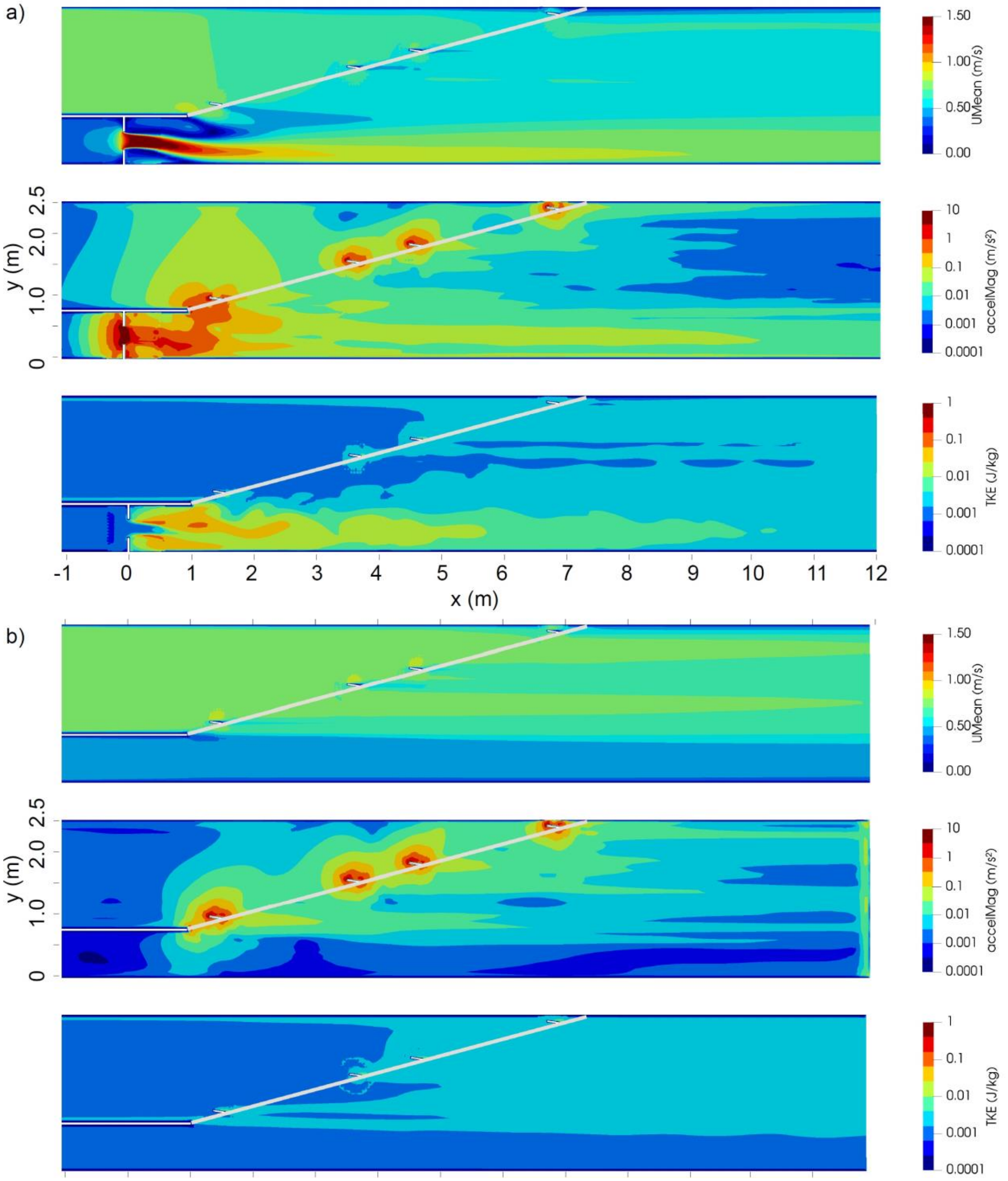

Figure 4: Horizontal flow field slices of setup 1 (a) and setup 2 (b) illustrate the effect of the slot and jet and its removal. Flow from left to right. Plane at z = 0.07 m above bottom where trout

preferred to stay, water depth = 0.60 m. Sub-panels in (a) and (b) show velocity magnitude U_m (top panels), acceleration magnitude (middle panels), and TKE (bottom panels). Note logarithmic scale for acceleration and TKE.

3.3 Random seed and time step sensitivity

To evaluate the influence of the different random seeds used, we compare the median coefficient of variation (σ/μ) for all ten setup/stimulus combinations. The values were low and ranged from 0.02-0.04, suggesting that our results were independent of the random seed chosen.

Halving the default time step to Δt = 0.25 s for the best wall distance parameter set deteriorated the RMSE in setup 1 and improved it in setup 2. Doubling the time step deteriorated the RMSE in both setups.

3.4 Parameter sensitivity

Parameter sensitivity was computed in both setups for the wall distance stimulus only. Setup 1 exhibited seven parameters of high influence ($\mu^*/\mu^*_{max} > 0.6$, Table 2), while setup 2 exhibited four parameters above this arbitrary limit. Ranks 17-19 comprised three parameters of low, negligible, effect on the result ($\mu^*/\mu^*_{max} \leq 0.1$). There was no first-order (independent) parameter (indicated by a high μ^* and low σ_M).

Both angles included in the migrating behavioral rule had a high influence and interaction in both setups. This result confirms that modeling orientation was important for the result. The vertical behavior correction angle was distinctly more important in setup 1 than in setup 2. This was likely caused by vertical currents induced by the plunging jet in setup 1.

All parameter sets which differed in one of the negligible parameters were excluded from the subsequent stimulus version tests. The parameter "wall detection range" was bound to the wall distance stimulus, anyway. The other two negligible parameters were not directly related to orientation, i.e. were expected to not interact strongly with the chosen stimulus. For the resulting number of parameter sets per stimulus version, n_{sets}, see Figure 3.

Table 2: Parameters of the behavioral model with wall distance stimulus, ranked by normalized influence, μ^*/μ^*_{max}, in setup 1. $\sigma_M/\sigma_{M,max}$ is normalized interaction with other parameters. The threshold for "low" values is indicated by a horizontal line. The last column, *No.*, is for reference to Table 3 in appendix S4, p. 8.

**Setup 1 (Jet)**			**Parameter name**	**Setup 2 (No jet)**			**No.**
**Rank**	**μ^*/μ^*_{max}**	**$\sigma_M/\sigma_{M,max}$**		**Rank**	**μ^*/μ^*_{max}**	**$\sigma_M/\sigma_{M,max}$**	
1	1.00	1.00	Migrating behavior max. random angle	2	0.74	0.60	14
2	0.82	0.87	Holding behavior extent	5	0.55	0.56	9
3	0.77	0.89	Migrating behavior wall distance angle	4	0.63	0.59	16
4	0.70	0.86	Fatigue (decreasing) memory coefficient	1	1.00	1.00	6
5	0.68	0.72	Vertical behavior correction angle	16	0.12	0.21	12
6	0.67	0.68	Spot memory coefficient	3	0.68	0.89	5
7	0.66	0.79	Motivation denominator ("fast fish")	7	0.47	0.69	3
8	0.56	0.64	Motivation memory coefficient	6	0.47	0.60	2
9	0.47	0.53	Stuck time threshold	10	0.22	0.34	13
10	0.41	0.46	Vertical behavior elevation threshold	13	0.13	0.17	11
11	0.33	0.41	Fatigue denominator	14	0.13	0.19	8
12	0.32	0.37	Motivation denominator ("slow fish")	9	0.27	0.37	4
13	0.24	0.34	Motivation initial value	11	0.21	0.30	1
14	0.24	0.29	Initialization "slow fish right"/"right fish"	15	0.13	0.17	20
15	0.21	0.20	Initialization "slow fish left"/"left fish"	12	0.13	0.34	19
16	0.20	0.23	Initialization "left fish"/"total fish"	8	0.44	0.44	18
17	0.09	0.10	Drifting behavior straight drift probability	17	0.10	0.23	10
18	0.07	0.07	Fatigue (increasing) memory coefficient	18	0.05	0.08	7
19	0.04	0.04	Migrating behavior wall detection range	19	0.02	0.02	15

3.5 Stimulus versions

We contrasted five stimulus versions for upstream swim angle selection in the model and ranked them (Table 3, Figure 5). The Kruskal-Wallis test obtained p = 16.2E-54 for setup 1 and p = 1.1E-78 for setup 2, suggesting that the stimulus versions did influence accordance with the laboratory observations significantly.

Table 3: Stimulus versions ranked by the RMSE 10th percentile per setup (appendix S6). The p-value was calculated *post hoc* between wall distance and the particular stimulus version. Significant differences between these versions at $p < \alpha$ = 1.0E-02 are denoted by an asterisk (*). p-values are rounded to two significant digits, percentile values to integer.

Setup	Stimulus version	RMSE 10th percentile	p-Value	Rating
1 - Jet	Wall distance	20	-	good
	Acceleration	21	1.2E-01	good
	Baseline	21	4.0E-01	good
	TKE	25	*4.1E-30	poor
	Velocity	26	*2.7E-27	poor
2 - No jet	Wall distance	14	-	good
	Velocity	16	8.9E-02	good
	Baseline	18	*1.2E-12	moderate
	Acceleration	20	*9.5E-29	moderate
	TKE	26	*2.0E-60	poor

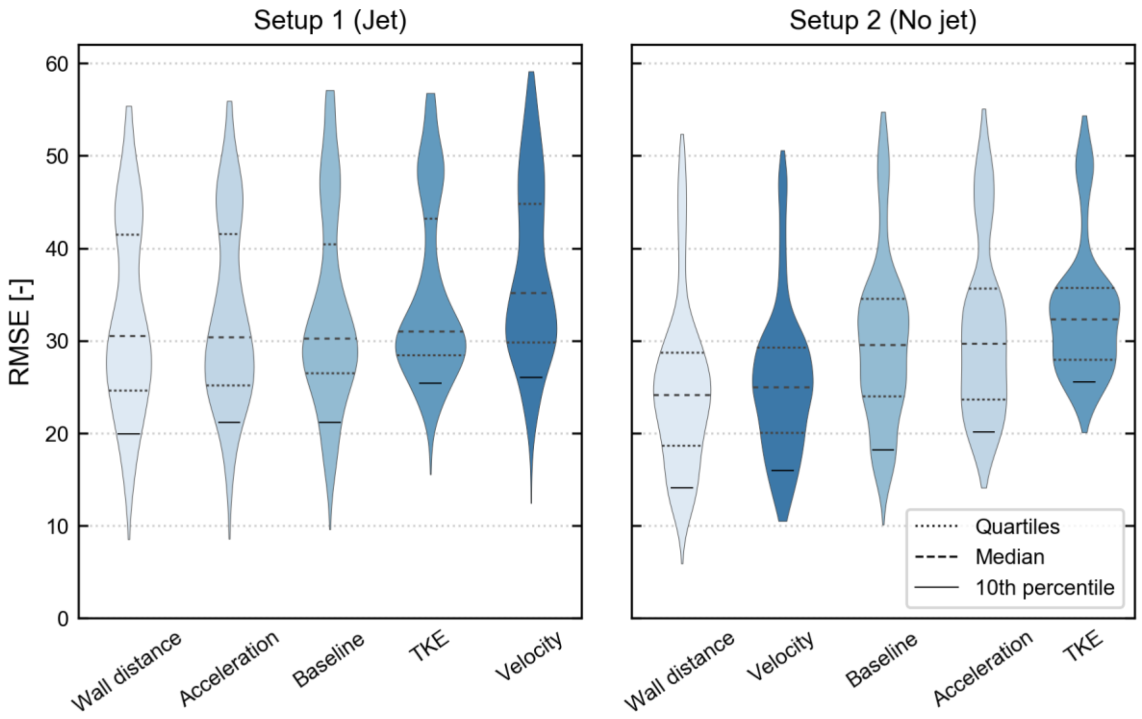

Figure 5: Violin plots indicate the frequency of root-mean-square error values (RMSE) in both setups (appendix S6). Ordering by increasing 10th percentile reveals similarities and significant differences between the stimulus versions. Results are better the more values are close to zero. The lower violin tip indicates the result of the best parameter set(s) for each version.

Wall distance was the best stimulus version in both setups. Acceleration and velocity differed widely in their ranks between setups, while baseline and TKE did not. In setup 1, the order of acceleration and baseline was not distinct, as well as the order of TKE and velocity (Figure 5). This is reflected in our qualitative "rating"/classification used for discussion (Table 3). The estimated distributions of the RMSE results are mainly multi-modal, which was the reason to rank them by their 10th percentile. The overall better results in setup 2 indicate that it was less demanding on the model than setup 1.

The wall distance version with its best parameter set reproduced all five patterns with high accuracy (RMSE = 9 for setup 1, RMSE = 6 for setup 2) (Figure 5, Figure 6). For comparison, e.g. Goodwin et al. (2014) aimed for RMSE ≤ 10. This finding shows that our model is able to produce all essential characteristics captured by our patterns (Grimm and Railsback 2005, p. 47).

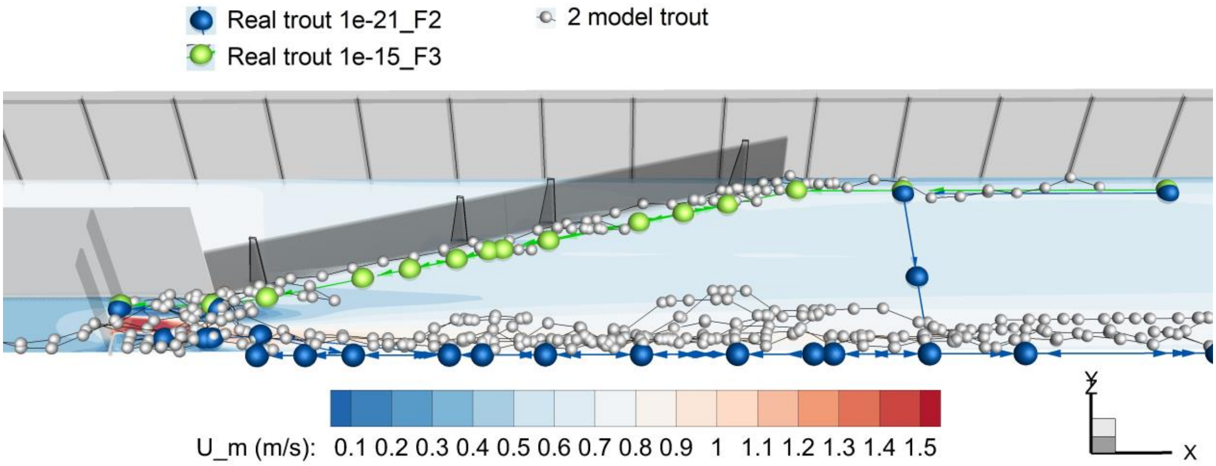

Figure 6: Two selected real and two selected model trout tracks in setup 1 (with jet). Each sphere shows one noted position or time step, respectively. Model results are from the best parameter set of the wall distance stimulus. Plane shows mean velocity, U_m, at z = 0.07 m from the bottom. Flow direction is from left to right, fish start from right on both flume sides. See appendices S1 and S2 for observed trout movement data.

4 Discussion

4.1 Stimuli for orientation

In this work, we combined the very different fields of ethology, hydraulic engineering, and behavior modeling to approach a classical problem of behavioral ecology: How can orientation behavior be explained through external stimuli? Our resulting movement patterns, flow fields, and significant differences between the model stimulus versions allow to develop explanations with respect to the ambient conditions in our two setups.

Results for the baseline version were moderate in setup 1 to good in setup 2, indicating that rheotactic orientation and a random component may already explain not only orientation (pattern P1), but also large parts of the remaining observed behavior captured by P2-P5. Positive rheotaxis was expected to be important as both flow direction and observed movement directions were largely uniform and aligned, fostering this basic behavior (Arnold 1974). Also, this result confirms that the random component was chosen in the right order to cover effects not modeled explicitly. Still, it was surprising that the other hydraulic variables commonly associated with behavior, i.e. velocity magnitude, TKE, and acceleration (e.g., Dockery et al. 2017; Goettel et al. 2015; Goodwin et al. 2014), were not required for this result. The apparent explanation that the flow field was not challenging enough to influence trout behavior will be discussed along the alternative stimulus versions tested.

The velocity model version yielded poor results in setup 1 and good results in setup 2. One reason for the failure in setup 1 was that model fish mostly avoided the jet of higher velocity on the right-hand side, while observed fish did not (P1). The laboratory observations indicate that trout did not aim to minimize their energy consumption by following low-velocity paths upstream (Wang and Chanson 2018; Zielinski et al. 2018). Hence, the good model results in setup 2 are not the result of a real mechanism. They are a

model effect of the low-velocity zone on the right-hand side, which fostered wall attraction and thus agreement to the observations. There is no apparent reason why trout would not seek lower velocity in setup 1 (with even larger bulk velocity) but would do so in setup 2. In conclusion, our model and laboratory results suggest that velocity was not a relevant stimulus to our trout.

To determine if a velocity/body length relation could explain why real trout ignored the higher velocity of the jet in setup 1, we made the following theoretical consideration. For trout (BL = 0.27 m) holding station, the relative flow velocity was about U_{fish} = 3.0 BL/s in large parts of the jet. At our water temperature, trout can maintain a sustained swimming speed of U_{fish} = 4.5 BL/s for up to 200 min according to Ebel's model equation for the rheophilic guild (Ebel 2014). Thus, in theory, trout could have moved upstream while experiencing almost no fatigue (Webb 1975) for longer than the test duration, indicating that the flow velocity was indeed too low to trigger a reaction. Some support for this explanation comes from two studies, where low-velocity seeking was observed at much larger relative velocities. Broadly estimated relative velocities were (0.37 m/s)/(0.07 m/BL) ≈ 5.3 BL/s for Duboulay's rainbowfish (Wang and Chanson 2018) and (0.39 m/s)/(0.06 m/BL) ≈ 6.5 BL/s for longnose dace (Dockery et al. 2017). In contrast, Kerr et al. (2016) reported low-velocity seeking already at (0.40 m/s)/(0.14 m/BL) ≈ 2.9 BL/s for small hatchery brown trout, which is similar to our relative velocity. The latter study shows that a general explanation has likely to include more factors than just relative velocity, even if it could be an explanation for our results.

Our next test for constant TKE attraction scored poor results in both setups. As both walls and bottom were smooth, they did not provide a distinct TKE stimulus for wall attraction, resulting in large deviations in P1 and the RMSE. These results challenge the statement that TKE is "by far the best stimulus" for fishway models (Gao et al. 2016) and the hypothesis that a constant turbulence level can be employed for orientation (Goettel et al. 2015). We did not test attraction towards increasing or decreasing TKE, but would expect similarly poor results for P1 as the TKE field offers no decent stimulus towards both sides (lateral asymmetry), which makes it difficult to match the laboratory-observed distribution along both sides.

Like for velocity, we compared TKE levels with literature values to determine if a relation to body length could explain our results. Our setups exhibited levels of TKE = 0.001-0.1 J/kg. Studies which explained behavior using TKE reported higher values for smaller fish: TKE = 0.1-0.3 J/kg for trout, BL = 0.20 m (Gao et al. 2016) and TKE = 0.015 J/kg for dace, BL = 0.07 m (Goettel et al. 2015). This qualitative comparison is in agreement with the explanation that the flow field did not cause behavioral reactions as it was not challenging for our trout. However, TKE is less studied for orientation than velocity and further research is required.

Results of the acceleration model version (good and moderate, respectively) were similar in absolute RMSE terms between setups, but differed widely in their in-setup rank because of better performances of the velocity and baseline versions in setup 2. Finding a theoretical explanation is difficult, as the few existing studies dealing with acceleration as an orientation stimulus focus on downstream movement (Kemp et al. 2006; Goodwin et al. 2014), which is associated with different responses than upstream movement. As for velocity (but with the better setup swapped), we suspect that a model effect, and not a

real mechanism, is responsible for the different ranking: Acceleration amplifies the velocity gradient at the lateral walls and produces a distinct stimulus for model wall attraction in setup 1, but not in setup 2, thus failing to match P1 observations. Again, the most probable explanation for our inconsistent results with acceleration, velocity, and TKE is that the live trout did not orient by means of the flow field (except direction), but by other factors. The most relevant is non-hydraulic perception of wall distance.

Using the wall distance version, our model performed well in terms of absolute RMSE and significantly better than any alternative version in at least one setup. This result is in line with the assumed limited hydraulic influence on orientation. Without hydraulic influence, it is particularly evident that any gradient which is based on following a stimulus carries the risk of imposing (Grimm and Railsback 2005) large residence times in the near-wall zones of pattern P1. This risk is mitigated by two mechanisms for moving to the middle zone, namely the random overlay angle and inclined drift. The good RMSE results for this version suggest that these mechanisms are in the right balance. We conclude that wall attraction by distance was a real behavior. Multiple reasons are possible including guidance, cover, or hydraulic advantages, but further experiments are required to evaluate them.

For the physiological mechanism of wall attraction, multiple explanations are conceivable. To us, visual distance estimation is the best explanation, considering that vision is a predominant stimulus in fish (Douglas and Hawryshyn 1990; Liao 2007; Elder and Coombs 2015). Given the clear visibility of the walls, it seems unlikely that another far-field stimulus (e.g. acoustic) dominated distance estimation. Distance estimation by means of a hydraulic signature is also unlikely according to our CFD model results. As discussed before, they show that wall influence was limited to some centimeters distance and/or was inconsistent between setups in flow variables (velocity, TKE, acceleration) which are commonly associated with behavioral responses (e.g., Dockery et al. 2017; Goettel et al. 2015; Goodwin et al. 2014). As a study on blind cave fish shows, hydrodynamic imaging by the lateral line close to smooth walls is limited to a small fraction of the body length (Windsor et al. 2010).

Taken together, our results indicate that the rarely considered wall distance can be a relevant stimulus for orientation in fishways and similarly confined migration paths. It requires, of course, ambient conditions which allow its perception by e.g. the visual or acoustic sense. Velocity and TKE stimuli being small in relation to fish body length could pose another necessary condition.

4.2 Stimulus combinations

In our model, we limited orientation stimuli to the baseline flow direction and one additional stimulus at a time. This is a simplification of reality, where most complex behavior is unlikely to depend on a single stimulus/sense alone (Liao 2006; Goettel et al. 2015), and is more likely to have a polysensory background (Kasumyan 2011). While making a model more realistic is not the same as making it more useful, combining stimuli to act on the same behavior is a worthwhile research direction for improving the generality of fish orientation IBMs.

A recent example is the use of calibrated weight factors for three hydraulic stimuli, which produced qualitative agreement to three carp tracks (Tan et al. 2018). A step further would be to model shifting

influence of stimuli depending on the ambient conditions, which was shown to exist in different species (Elder and Coombs 2015; Fuentes-Pérez et al. 2018). However, in our own model, a combination of the second-ranked acceleration version for setup 1 and the second-ranked velocity version for setup 2 would still not be as good as the first-ranked wall distance version. In addition, both stimuli were likely not real stimuli (as discussed above) and hence such a model version would reduce robustness and explanatory power. We conclude that our current model is not likely improved through stimuli combinations, but they could be important for transfer to more complex environments such as in-situ fishways.

4.3 Movement patterns in the laboratory

Transfer of our model also depends on how universal the underlaying movement patterns are. The observed tendency to swim close to the walls (pattern P1) matched descriptions of rainbow trout behavior (Przybilla et al. 2010) and barbel behavior in a confined model fishway (Fuentes-Pérez et al. 2018). It was not observed in faster flow and a more narrow flume for brook and brown trout (Castro-Santos et al. 2013). This discrepancy indicates a potential dependence on the ambient conditions. For our slow, shallow flow, we conclude that P1 is not an untypical behavior.

Swimming at the bottom is reported frequently, e.g. in a natural stream for brown trout (Cotel et al. 2006), and in flumes for rainbow trout (Przybilla et al. 2010), barbels (Silva et al. 2012), brown and brook trout (Castro-Santos et al. 2013), and longnose dace (Dockery et al. 2017). The water column in our experiment was shallow (d = 0.60 m), hence it was unclear whether fish preferred the bottom for orientation, for shelter, or for another reason. Despite possible behavioral or physiological reasons, P2 can be considered a typical behavior in our flume conditions.

For patterns P3 and P4, we did not find matching descriptions in the literature, because they represent a new way of systematically dealing with the striking feature "turns". P5 was also too specific to our setup for a meaningful comparison.

From the literature comparisons, it seems plausible that patterns P1 and P2 would be universal enough to support model transfer to similar flows, e.g. to an altered flume geometry. Transfer to the real world could be challenging, e.g. due to limited wall distance estimation and/or larger flow velocities.

4.4 General limitations

Finally, we want to point out some general limitations of this work to facilitate critical reception. First, our results are limited in scale to the estimated fish observation accuracy, which was coarser than the scale of changes in the hydraulic variables in a distance of 1-2 m downstream of the slot. Thus, potential effects of sudden changes in this area on the behavior could not be incorporated into the model.

Second, we found that different parameter combinations produced similarly good results, which indicates that parameter values are not robust and transfer of the behavioral model to other species and/or hydraulic environments requires recalibration. This does not affect our reproducible testing procedure of complex hypotheses, which is one of the major assets of IBMs (Willis 2011).

Third, the behavioral model was sensitive to changes in time step width. Main influences changing with the number of time steps include accuracy of flow field perception (Smith et al. 2014) and the number of orientation and random decisions.

Fourth, matching P1 depended, among other effects, on the initial distribution of fish positions to either side. The share of fish starting on the left flume side in the best wall distance version was off the real distributions observed in the flume (33 % vs. 68 % with jet and 33 % vs. 46 % without jet). Producing matching results from differing initial conditions could point to a difference between model and laboratory behavior or it could be a sign of minor relevance of the initial conditions to the model result.

Fifth, our model is currently limited to steady flow fields. Modeling e.g. utilization of transient vortices would require considerable model modification (Padgett et al. 2020).

Sixth and last, although swimming in groups (shoaling) was not pronounced in our data set and not modeled, it can influence orientation behavior. Determining its influence would require laboratory experiments with single fish releases. Points one to four are specific to this very data set and model instance, but point five and especially point six could improve future development of our IBM.

5 Conclusions

Our goal in this study was to better understand upstream fish orientation and navigation in confined space, e.g. in fishways, by means of concrete behavioral rules. Our thoroughly tested IBM was able to reproduce five brown trout behavioral patterns in two experimental flow fields, which represented a broad range of spatial behavior in a wide flume. The first two patterns – avoiding the middle and surface zones of the flume – were very distinct, and should be investigated further to determine their dependency on e.g. water width and depth.

The significant advantage of the wall distance stimulus version illustrates that a focus on hydraulic stimuli for predicting fish orientation can be too narrow, especially in hydraulic conditions not demanding for the observed individuals. Wall distance estimation, mediated by whatever sense, should be considered more frequently as a stimulus in confined space. This is true for studies in IBMs, laboratory flumes, and in the field, if the conditions enable wall perception by e.g. the visual or acoustic sense.

The evaluation of observed patterns and key parameter variety indicated that transfer of our present model is likely limited to similar species and conditions, i.e. brown trout of a given size in a relatively slow, shallow flume flow. This does not affect the IBM's ability to contrast different behavior hypotheses.

In summary, the interdisciplinary IBM technique facilitates the investigation of hypotheses for stimuli-response mechanisms for external stimuli with flexibility and rigor. It is an important option to approach the many urgent questions of fishway designers.

6 Acknowledgments

We thank all persons involved in the realization of the brown trout movement experiments.

We thank Dr. Andy Goodwin of USACE for providing the Fortran 90 model code used in Goodwin et al. (2014) as a starting point.

7 References

Arenas, Antonio; Politano, Marcela; Weber, Larry; Timko, Mark (2015): Analysis of movements and behavior of smolts swimming in hydropower reservoirs. In *Ecological Modelling* 312, pp. 292–307. DOI: 10.1016/j.ecolmodel.2015.05.015.

Arnold, G. P. (1974): Rheotropism in fishes. In *Biological Reviews* 49 (4), pp. 515–576. DOI: 10.1111/j.1469-185X.1974.tb01173.x.

Bleckmann, Horst; Zelick, Randy (2009): Lateral line system of fish. In *Integrative zoology* 4 (1), pp. 13–25. DOI: 10.1111/j.1749-4877.2008.00131.x.

Bunt, C. M.; Castro-Santos, T.; Haro, A. (2016): Reinforcement and Validation of the Analyses and Conclusions Related to Fishway Evaluation Data from Bunt et al.: 'Performance of Fish Passage Structures at Upstream Barriers to Migration'. In *River Res. Applic.* 32 (10), pp. 2125–2137. DOI: 10.1002/rra.3095.

Bunt, Christopher M.; Castro-Santos, Theodore; Haro, Alex (2012): Performance of fish passage structures at upstream barriers to migration. In *River Res. Applic.* 28 (4), pp. 457–478. DOI: 10.1002/rra.1565.

Campolongo, Francesca; Cariboni, Jessica; Saltelli, Andrea (2007): An effective screening design for sensitivity analysis of large models. In *Environmental Modelling & Software* 22 (10), pp. 1509–1518. DOI: 10.1016/j.envsoft.2006.10.004.

Castro-Santos, Theodore; Sanz-Ronda, Francisco Javier; Ruiz-Legazpi, Jorge; Jonsson, Bror (2013): Breaking the speed limit — comparative sprinting performance of brook trout (Salvelinus fontinalis) and brown trout (Salmo trutta). In *Can. J. Fish. Aquat. Sci.* 70 (2), pp. 280–293. DOI: 10.1139/cjfas-2012-0186.

Chapman, Ben B.; Hulthén, Kaj; Blomqvist, David R.; Hansson, Lars-Anders; Nilsson, Jan-Åke; Brodersen, Jakob et al. (2011): To boldly go. Individual differences in boldness influence migratory tendency. In *Ecology letters* 14 (9), pp. 871–876. DOI: 10.1111/j.1461-0248.2011.01648.x.

Cotel, Aline J.; Webb, Paul W.; Tritico, Hans M. (2006): Do Brown Trout Choose Locations with Reduced Turbulence? In *Transactions of the American Fisheries Society* 135 (3), pp. 610–619. DOI: 10.1577/T04-196.1.

Dockery, D. R.; McMahon, T. E.; Kappenman, K. M.; Blank, M. (2017): Evaluation of swimming performance for fish passage of longnose dace Rhinichthys cataractae using an experimental flume. In *Journal of fish biology* 90 (3), pp. 980–1000. DOI: 10.1111/jfb.13217.

Douglas, Ron; Hawryshyn, Craig (1990): Behavioral studies of fish vision: An analysis of visual capabilities. In Ron Douglas, Mustafa Djamgoz (Eds.): The Visual System of Fish. Dordrecht: Springer Netherlands, pp. 373–418.

Ebel, Guntram (2014): Modellierung der Schwimmfähigkeit europäischer Fischarten — Zielgrößen für die hydraulische Bemessung von Fischschutzsystemen. In *Wasserwirtschaft* 104 (7-8), pp. 40–47. DOI: 10.1365/s35147-014-1095-1.

Elder, John; Coombs, Sheryl (2015): The influence of turbulence on the sensory basis of rheotaxis. In *Journal of comparative physiology. A, Neuroethology, sensory, neural, and behavioral physiology* 201 (7), pp. 667–680. DOI: 10.1007/s00359-015-1014-7.

Fuentes-Pérez, Juan Francisco; Eckert, Mario; Tuhtan, Jeffrey A.; Ferreira, Maria Teresa; Kruusmaa, Maarja; Branco, Paulo (2018): Spatial preferences of Iberian barbel in a vertical slot fishway under variable hydrodynamic scenarios. In *Ecological Engineering* 125, pp. 131–142. DOI: 10.1016/j.ecoleng.2018.10.014.

Gao, Zhu; Andersson, Helge I.; Dai, Huichao; Jiang, Fengjian; Zhao, Lihao (2016): A new Eulerian–Lagrangian agent method to model fish paths in a vertical slot fishway. In *Ecological Engineering* 88, pp. 217–225. DOI: 10.1016/j.ecoleng.2015.12.038.

Gisen, David C. (2018): Modeling upstream fish migration in small-scale using the Eulerian-Lagrangian-agent method (ELAM). Dissertation. Universität der Bundeswehr München, Munich, Germany. Available online at https://hdl.handle.net/20.500.11970/105158.

Goettel, Michael T.; Atkinson, Joseph F.; Bennett, Sean J. (2015): Behavior of western blacknose dace in a turbulence modified flow field. In *Ecological Engineering* 74, pp. 230–240. DOI: 10.1016/j.ecoleng.2014.10.012.

Goodwin, Richard Andrew; Politano, M.; Garvin, J. W.; Nestler, J. M.; Hay, D.; Anderson, James J. et al. (2014): Fish navigation of large dams emerges from their modulation of flow field experience. In *Proceedings of the National Academy of Sciences* 111 (14), pp. 5277–5282. DOI: 10.1073/pnas.1311874111.

Grimm, Volker; Berger, Uta; Bastiansen, Finn; Eliassen, Sigrunn; Ginot, Vincent; Giske, Jarl et al. (2006): A standard protocol for describing individual-based and agent-based models. In *Ecological Modelling* 198 (1-2), pp. 115–126. DOI: 10.1016/j.ecolmodel.2006.04.023.

Grimm, Volker; Railsback, Steven F. (2005): Individual-based modeling and ecology. Princeton: Princeton University Press (Princeton series in theoretical and computational biology).

Grimm, Volker; Railsback, Steven F. (2012): Pattern-oriented modelling: a 'multi-scope' for predictive systems ecology. In *Philosophical transactions of the Royal Society of London. Series B, Biological sciences* 367 (1586), pp. 298–310. DOI: 10.1098/rstb.2011.0180.

Grimm, Volker; Railsback, Steven F.; Vincenot, Christian E.; Berger, Uta; Gallagher, Cara; DeAngelis, Donald L. et al. (2020): The ODD Protocol for Describing Agent-Based and Other Simulation Models. A Second Update to Improve Clarity, Replication, and Structural Realism. In *JASSS* 23 (2). DOI: 10.18564/jasss.4259.

Jones, M. J.; Baumgartner, L. J.; Zampatti, B. P.; Beyer, K. (2017): Low light inhibits native fish movement through a vertical-slot fishway. Implications for engineering design. In *Fish Manag Ecol* 24 (3), pp. 177–185. DOI: 10.1111/fme.12205.

Kasumyan, A. O. (2011): Tactile reception and behavior of fish. In *J. Ichthyol.* 51 (11), pp. 1035–1103. DOI: 10.1134/S003294521111004X.

Kemp, P. S. (2016): Meta-analyses, Metrics and Motivation: Mixed Messages in the Fish Passage Debate. In *River Res. Applic.* 32 (10), pp. 2116–2124. DOI: 10.1002/rra.3082.

Kemp, Paul S.; Gessel, Michael H.; Sandford, Benjamin P.; Williams, John G. (2006): The behaviour of Pacific salmonid smolts during passage over two experimental weirs under light and dark conditions. In *River Res Applic* 22 (4), pp. 429–440. DOI: 10.1002/rra.913.

Kerr, James R.; Manes, Costantino; Kemp, Paul S. (2016): Assessing hydrodynamic space use of brown trout, Salmo trutta, in a complex flow environment: a return to first principles. In *Journal of Experimental Biology* 219 (Pt 21), pp. 3480–3491. DOI: 10.1242/jeb.134775.

Liao, James C. (2006): The role of the lateral line and vision on body kinematics and hydrodynamic preference of rainbow trout in turbulent flow. In *Journal of Experimental Biology* 209 (20), pp. 4077–4090. DOI: 10.1242/jeb.02487.

Liao, James C. (2007): A review of fish swimming mechanics and behaviour in altered flows. In *Philosophical Transactions of the Royal Society B: Biological Sciences* 362 (1487), pp. 1973–1993. DOI: 10.1098/rstb.2007.2082.

Montgomery, J.; Carton, G.; Voigt, R.; Baker, C.; Diebel, C. (2000): Sensory processing of water currents by fishes. In *Philosophical Transactions of the Royal Society B: Biological Sciences* 355 (1401), pp. 1325–1327. DOI: 10.1098/rstb.2000.0693.

Padgett, Thomas E.; Thomas, Robert E.; Borman, Duncan J.; Mould, David C. (2020): Individual-based model of juvenile eel movement parametrized with computational fluid dynamics-derived flow fields informs improved fish pass design. In *Royal Society open science* 7 (1), p. 191505. DOI: 10.1098/rsos.191505.

Przybilla, Anja; Kunze, Sebastian; Rudert, Alexander; Bleckmann, Horst; Brücker, Christoph (2010): Entraining in trout: a behavioural and hydrodynamic analysis. In *Journal of Experimental Biology* 213 (Pt 17), pp. 2976–2986. DOI: 10.1242/jeb.041632.

Rodríguez, Álvaro; Bermúdez, María; Rabuñal, Juan R.; Puertas, Jerónimo (2015): Fish tracking in vertical slot fishways using computer vision techniques. In *Journal of Hydroinformatics* 17 (2), pp. 275–292. DOI: 10.2166/hydro.2014.034.

Schütz, Cornelia; Herbst, Marcus; Pitsch, Matthias; Henning, Martin; Czerny, Rebekka (2021): Addition of auxiliary discharge into a fishway - a contribution to fish pass design at large rivers. Manuscript submitted.

Silva, Ana T.; Katopodis, Christos; Santos, José M.; Ferreira, Maria Teresa; Pinheiro, António N. (2012): Cyprinid swimming behaviour in response to turbulent flow. In *Ecological Engineering* 44, pp. 314–328. DOI: 10.1016/j.ecoleng.2012.04.015.

Silva, Ana T.; Lucas, Martyn C.; Castro-Santos, Theodore; Katopodis, Christos; Baumgartner, Lee J.; Thiem, Jason D. et al. (2018): The future of fish passage science, engineering, and practice. In *Fish and Fisheries* 19 (2), pp. 340–362. DOI: 10.1111/faf.12258.

Smith, David L.; Goodwin, Richard Andrew; Lai, Young; Nestler, John Michael (2014): Fish path selection and fatigue in complex, continuous velocity fields. In Atle Harby, Håkon Sundt (Eds.): 10th International Symposium on Ecohydraulics. 10th International Symposium on Ecohydraulics. Trondheim.

Tan, Junjun; Tao, Lin; Gao, Zhu; Dai, Huichao; Shi, Xiaotao (2018): Modeling Fish Movement Trajectories in Relation to Hydraulic Response Relationships in an Experimental Fishway. In *Water* 10 (11). DOI: 10.3390/w10111511.

The OpenFOAM Foundation (2014): OpenFOAM. Version 2.3.1. Available online at https://github.com/OpenFOAM/OpenFOAM-2.3.x, checked on 4/28/2020.

Thiele, Jan C.; Kurth, Winfried; Grimm, Volker (2014): Facilitating Parameter Estimation and Sensitivity Analysis of Agent-Based Models. A Cookbook Using NetLogo and 'R'. In *JASSS* 17 (3). DOI: 10.18564/jasss.2503.

Wang, H.; Chanson, H. (2018): Modelling upstream fish passage in standard box culverts. Interplay between turbulence, fish kinematics, and energetics. In *River Res. Applic.* 128 (5), p. 245. DOI: 10.1002/rra.3245.

Webb, Paul W. (1975): Hydrodynamics and Energetics of Fish Propulsion. Edited by J. C. Stevenson. Fisheries Research Board of Canada. Ottawa, Canada (Bulletin of the Fisheries Research Board of Canada, 190). Available online at https://scholarworks.umass.edu/fishpassage_journal_articles/569.

Willis, Jay (2011): Modelling swimming aquatic animals in hydrodynamic models. In *Ecological Modelling* 222 (23-24), pp. 3869–3887.

Windsor, Shane P.; Norris, Stuart E.; Cameron, Stuart M.; Mallinson, Gordon D.; Montgomery, John C. (2010): The flow fields involved in hydrodynamic imaging by blind Mexican cave fish (Astyanax fasciatus). Part II: Gliding parallel to a wall. In *The Journal of experimental biology* 213 (22), pp. 3832–3842. DOI: 10.1242/jeb.040790.

Zielinski, D. P.; Voller, VR.; Sorensen, P. W. (2018): A physiologically inspired agent-based approach to model upstream passage of invasive fish at a lock-and-dam. In *Ecological Modelling* 382, pp. 18–32. DOI: 10.1016/j.ecolmodel.2018.05.004.

8 Supporting information appendix

8.1 S1 Brown trout movement observations, setup 1

Unpublished movement data from Schütz et al. (2021). See S1_brown_trout_setup1.xlsx.

Column name	Definition	Units
fish_id	"1e" is setup 1; "2c" is setup 2. Next part of the ID is trial number, and last is fish number (3 per trial).	-
time_stamp	Time of observation, interpolated from minutes to seconds by event count per minute	UTC DD.MM.YYYY HH:MM:ss
sec	Seconds since start (lifting of staging area mesh screen)	s
swarmsize	Size of group observed, 1-3 fish, "group" was defined subjectively by swimming together	-
x_raw	Longitudinal position observed, axis zero at flume start	m
y_raw	Lateral position, observed to be close to left wall/screen (L), right wall/screen (R), or close to neither wall/screen (M). Used for pattern P1.	Categorical
z_raw	Vertical position observed in the water column, either close to water surface (o), close to bottom (u), or in between (m). Used for pattern P2.	Categorical
x_value	Longitudinal position, axis zero at slot. Used for patterns P3, P4, and P5.	m
y_value	Lateral position, defined from y_raw as being either 0.1 m away from a wall/screen or in the middle. Coordinates account for inclined screen. Used only for computation of cells in Figure 2, not for determining pattern P1.	m

8.2 S2 Brown trout movement observations, setup 2

See S2_brown_trout_setup2.xlsx. Description above.

8.3 S3 Hydraulic field data

See S3_hydraulic_fields.zip containing S3a_hydraulic_fields_setup1.csv and S3b_hydraulic_fields_setup2.csv. For coordinate system origin, see Figure 1.

Column name	Description	Units
ccx, ccy, ccz	Cell center coordinates	m
u, v, w	Velocity vector components	m/s
acclMag	Acceleration magnitude	m/s^2
alpha.water	Water phase, share of cell volume	-
k	Turbulence kinetic energy (TKE)	m^2/s^2

8.4 S4 Behavioral model description

See S4_Gisen_behavioral_model_description.pdf

8.5 S5 Behavioral model code

See https://github.com/baw-de/ELAM-flume. The code is written in C++ and Fortran 90. The Fortran code uses the FORTRAN 77 fixed format for historic reasons.

8.6 S6 Root mean square errors

See S6_RMSE_5_stimuli_2_setups.csv. The order is Wall distance (setup 1, setup 2) – Acceleration 1, 2 – Velocity 1, 2 – TKE 1, 2 – Baseline 1, 2.

Supporting Information 1

Click here to access/download
Supporting Information
S1_Brown_trout_setup1.xlsx

Supporting Information 2

Click here to access/download
Supporting Information
S2_Brown_trout_setup2.xlsx

Click here to access/download
Supporting Information
S4_Behavioral_model_description.pdf

Click here to access/download
Supporting Information
S6_RMSE_5_stimuli_2_setups.csv

Supporting Information 3

Click here to access/download

Supporting Information - Compressed/ZIP File Archive

S3_hydraulic_fields.zip

Supporting Information 5

Click here to download Data Review URL https://github.com/baw-de/ELAM-flume
