# Supplementary material for: Development of behavioral rules for upstream orientation of fish in confined space
Source: PLoS One. 2022 Feb 18;17(2):e0263964. doi: 10.1371/journal.pone.0263964 (PMC8856537; doi:10.1371/journal.pone.0263964)
Supplement: Attachment — Submitted filename: PONE-D-21-08612_R1.pdf [file pone.0263964.s009.pdf]

PLOS ONE

Development of behavioral rules for upstream orientation of fish in confined space

--Manuscript Draft--

**Manuscript Number:**	PONE-D-21-08612R1
**Article Type:**	Research Article
**Full Title:**	Development of behavioral rules for upstream orientation of fish in confined space
**Short Title:**	Development of behavioral rules for upstream orientation of fish in confined space
**Corresponding Author:**	David C. Gisen<br>Bundesanstalt fur Wasserbau<br>Karlsruhe, GERMANY
**Keywords:**	Fish behavior; fishway; individual-based model; computational fluid dynamics; fish migration
**Abstract:**	Improving the effectiveness of fishways requires a better understanding of fish behavior near hydraulic structures, especially of upstream orientation. One of the most promising approaches to this problem is the use of model behavioral rules.<br>We developed a three-dimensional individual-based model based on observed brown trout (Salmo trutta fario) movement in a laboratory flume and tested it against two hydraulically different flume setups. We used the model to examine which of five behavioral rule versions would best explain upstream trout orientation. The versions differed in the stimulus for swim angle selection. The baseline stimulus was positive rheotaxis with a random component. It was supplemented by attraction towards either lower velocity magnitude, constant turbulence kinetic energy, increased flow acceleration, or smaller wall distance.<br>As expected, modeling rheotaxis was central to matching the laboratory observations. The baseline version was already able to explain large parts of the observed behavior. Mixed results for velocity magnitude, turbulence kinetic energy, and flow acceleration indicated that the brown trout did not orient primarily by means of flow features, probably because the flow field did not challenge them physically.<br>The wall distance version produced significantly improved results, suggesting that wall distance was the dominant orientation stimulus for brown trout in our hydraulic conditions. The absolute root mean square error (RMSE) was small for the best parameter set (RMSE = 9 for setup 1, RMSE = 6 for setup 2). Our best explanation for this result is dominance of the visual sense favored by absence of challenging hydraulic stimuli. We conclude that under similar conditions (moderate flow and visible walls), wall distance could be a relevant stimulus in confined space, particularly for fishway studies and design in IBMs, laboratory, and the field.
**Order of Authors:**	David C. Gisen
	Cornelia Schütz
	Roman B. Weichert
**Response to Reviewers:**	PLEASE SEE BELOW FOR ORIGINAL TABULAR FORM<br><br>Response to reviewers<br>First, we would like to thank the reviewers for their insightful comments, which helped us to improve the manuscript. Our particular responses can be found below. Generally, we tried to further streamline terms, correct errors, and to improve readability throughout the whole manuscript.<br><br>We note that the manuscript does not yet meet the style requirements of PLOS ONE, but decided to await the provisional Editorial Accept decision (per https://journals.plos.org/plosone/s/getting-started) to avoid confusion during review e.g. by changing the citation style.<br><br>Reviewer #1<br>CommentAddressed?How addressed or why not?Line (original)<br>1. The introduction is poorly organized and confusing. The discussion of hydraulic parameters is insufficient and unclear.YesWe've rewritten large parts of the

introduction, focusing on readability and the motivation for our study. We've also deleted information on stimuli that went too much into detail or - where helpful - moved them to the model description to provide a rationale on why each stimulus was chosen.

2. Why would learning effects matter in this scenario?NoFish already familiar with the flume geometry and hydraulic field could deviate in their behavior unpredictably, e.g. navigate straight towards the upstream end or don't move at all. Excluding such effects by testing every individual just once makes results more reproducible.110

3. I don't understand why the fish swimming study was designed the way it was. Was it specifically designed to calibrate/validate the model?Yes(See also comments #9 and #15 of reviewer #3.)
The previous, unrelated study was primarily designed to investigate the influence of auxiliary water velocity coming from the screen on passage success and delay of five species.
Conditions were chosen to create hydraulic conditions typical for a large multi-species fishway in Germany. It is now published in Ecological Engineering, where more information is available: https://doi.org/10.1016/j.ecoleng.2021.106257
We used the both setups to establish an application range for our model, see also comments #9 and #15 of reviewer #3.
We've added information on design choice and the word "unrelated" in section 2.1 for clarification.100

4. I cannot provide a good review of the movement patterns section (2.2) because it is not something I've studied.---

5. Please provide more information on CFD model setup, calibration and validation.NoWe feel the information provided on the model setup is sufficient for reproduction. Calibration in the classical sense was not performed, as boundary conditions are fully determined by the laboratory flume. Mesh, turbulence model, and screen negligence were validated using ADV probe data (line 362f). Comprehensive detail information can be found in (Gisen 2018, p. 33ff.).153ff

6. I would like more explanation for why decisions were made ....e.g. why use averaged/steady results from the CFD model?YesUsing transient CFD results is a promising direction to capture behavior currently modelled stochastically. However, interpolating thousands of 3D fish positions on 7200 flow field snapshots (dt = 0.5 s for 1 h) is currently far beyond our simulation capacity, even using a high-performance cluster. We've added a brief mention to the limitations section 4.4.

7. I love this stuff and I think its interesting work, I just want more information in the methods that is clearly organized and suggest a full rewrite where you focus on streamlining terminology, writing good paragraphs, and correcting grammar errors. YesWe've added information to the methods section and tried to streamline terminology in the whole manuscript. We considered to change the order of subsections in section 2.5, but refrained from doing so, as there are too many dependencies in both ways, e.g. between RMSE, RMSE(model-run), and parameter set evaluation. We rely on figure 3 to explain these dependencies.

8. I'm a bit concerned that it is unclear how much of this work is Goodwin's and how much is new work.YesWe've tried to clarify in the introduction that Goodwin (2014) worked on downstream migration on dam-scale, while we work on upstream migration on fishway-scale, which required e.g. a completely new behavioral model. The CFD model was also replaced, of course, and the software framework was heavily modified (section 2.4.1).

Reviewer #2
Reviewer #2: "Nicely thought out work. Please see manuscript PDF where I mention a few places where better word choice will help the reader. More broadly, I think the manuscript could be improved in terms of readability by improving the flow of information. Some detail and terms within the manuscript make it hard to follow at times, so perhaps consider moving some technical pieces/info together (or use more general terms where possible) to allow more portions of the manuscript to flow (read) easier."

Powered by Editorial Manager® and ProduXion Manager® from Aries Systems Corporation

CommentAddressed?How addressed or why not?Line (original)
1. Unclear, please restate another way.YesRephrased50
2. Unclear.YesReplaced “like for P1” with “using Eq. 1”136
3. Unclear, please restate another way.YesRephrased321
4. Unclear, please restate another way.YesRephrased322
5. underlying ?YesDeleted “a”537
6. misplaced "e.g." ?NoOther influences than width and depth are conceivable, such as smooth boundaries and illumination584
7. demanding enough forNoFrom our perception “enough” would suggest a need to create more demanding hydraulic conditions, which was not our intention in this study (see reviewer #1, comment #3).586f.

Reviewer #3
Reviewer #3: “In this manuscript, a multi-faceted study is used to develop a framework to model fish movement to test different behavioral rules to explain fish orientation and navigation. The study uses fish tracking data from an unpublished laboratory study of brown trout passage through a partitioned flume. Pattern orienting modelling was used to capture 5 distinct movement patterns. The patterns were used as a metric for which to evaluate the performance of the behavioral rules. The authors use the Eulerian-Lagrangian agent method (ELAM) developed by Goodwin et al. (2014) as a foundation for their individual based model (IBM). The authors IBM was used to evaluate three behavioral rules using five different guidance stimuli. The authors found that wall distance guidance stimulus performed best at replicating the movement patterns. Overall, the manuscript was well written and combined with the extensive detail in the appendices (e.g., ODD report) provides a reasonable account of a complex study. Several aspects of the study are not entirely novel. The laboratory study is addressed in a separate manuscript, the CFD analysis is standard practice, and the ELAM provides the general computational infrastructure for the IBM. Therefore, the novelty of this study lies entirely within the behavioral rule and guidance stimulus selection and analysis.
While the sensitivity analysis and modelling output are rigorous, the findings are somewhat muted. As detailed later, I found the laboratory study to be incongruous with the movement hypotheses being tested, likely leading to the less explanatory stimulus of wall distance being the best fit. Despite this issue, I feel the manuscript is still an important finding in the field of predictive modelling of fish movement. Due to the number of comments and level of effort required to address, I recommend the manuscript undergo major revisions.”

CommentAddressed?How addressed or why not?Line (original)
Introduction
1. Line 42 – A reference to Goodwin et al. (2014) would seem appropriate here.YesThe reference Silva et al. 2018 serves to underline the “high research priority” of behavioral rules. We did not intend to reference general studies using behavioral rules here, elsewise we would have to list much more than just Goodwin’s. We’ve tried to make this clearer by using a direct quote of Silva et al.

2. Line 48 – While the introduction touches briefly upon aspects of the fuller study, it lacks sufficient detail to understand why certain selections of behaviors and guiding stimuli were chosen. In this instance, citing the IPOS framework to describe what aspects of turbulence are important to fish behaviors would be relevant.YesFor choice of behaviors and stimuli see response to comment #1 of reviewer #1.

We’ve added a reference to IPOS (DOI:10.1002/rra.1584) for general understanding. However, for our IBM study (and others we are aware of), periodicity, orientation, and scale are still too detailed (see comment #5 below and new reference Roth et al. 2021).

3. What about motivation to move or context specific behaviors? The authors need to address the complexities caused by differences in the internal state of a fish to make different decisions to the same stimuli.YesThis is an important aspect and we’ve added a corresponding paragraph in the introduction. As we focused on orientation and fishway design, we modeled internal stimuli only rather basically (using proxies for motivation and fatigue). We now discuss this in the model description (2.4).

Powered by Editorial Manager® and ProduXion Manager® from Aries Systems Corporation

4. The introduction would further benefit from more details on how the current effort extends or differs from previous IBMs. Specifically, it is not clear how the model is differentiated from the ELAM used by Goodwin et al. (2014). The only reference to the ELAM in on Lines 73 and 84 stating the proposed model is “ELAM-type”. I feel the general reader is not going to understand what this means.YesSee response to comment #8 of reviewer #1.
We also point more directly to lacks of previous upstream IBMs (testing, resting behavior). We’ve removed the term “ELAM”, as it is not required to understand our work.

5. The authors need to more explicitly state what hypotheses they are testing and why. The preceding paragraphs lists evidence that points to a lot of hydraulic variables that could be influential to fish movement. The authors do not explain why they examine baseline rheotaxis, velocity magnitude, TKE, flow acceleration, and wall distance relative to other choices including turbulent intensity, velocity gradients, eddy sizes, etc.YesSee response to comment 1 of reviewer #1.
We’ve added a justification for each stimulus tested in the behavioral model description (2.4) and a summary in the introduction. Generally, we preferred the more comprehensive variable over specialized variables. E.g. all three turbulence intensity components are contained in the TKE (see e.g. IPOS paper, p. 432).
The velocity gradient tensor is contained in the acceleration metric.
Strain rate and rotation are contained in the velocity gradient tensor.
Reynolds shear stresses could be relevant to future work.
Eddy size, as well as eddy orientation, is an interesting, but advanced measure left for future work (see response #2 above). It is partly covered in the rheotaxis stimulus.
Methods

6. Line 110 – Where does the “x=9.74” come from? Figure 1 indicates an observation point at x=7.49.YesAt x=9.74 m, there was an external pole on the flume used as mark for quick orientation in the original study. We’ve removed this information, as it does not affect this manuscript.

7. Figure 1. The figure caption is the first mention of patterns P1 and P5 without defining them. I suggest moving this statement into the main text after P1 and P5 are defined.YesWe’ve inserted into the caption a reference to section 2.2 where the patterns are defined. If the caption was changed per your suggestion, readers could be confused about the meaning of the gray areas and line D.

8. Line 109-119 – The methods and scales at which fish movement was tracked is unclear. Where observers tracking movements in real-time on paper as well as noting location relative to the wall or screen or position in a group? How often was the position recorded? The authors state that observations were verified qualitatively with video records, why not use the video to obtain more detailed tracks. Overall, the methods on how tracking was accomplished needs significant more detail.YesWe’ve added this lacking information to the methods (real-time recording on paper; positions were recorded on every notable change; definitions of changes in the three dimensions).
We’ve also added to methods and appendix S1 the information that x_value and y_value were computed in postprocessing.
A manual video analysis of fish tracks would have been too time consuming and (because cameras were only positioned laterally) would not have been reliable for lateral movements; we also worked on an automated 3D-fishtracking of the videos (along 11 overlapping cameras) but with three fish swimming simultaneously this is demanding and proved to be less efficient than the manual records for this project.

9. How were the set-ups chosen for the laboratory tests and how does this relate to the central hypotheses being tested?Yes(See also response to comment #15 of reviewer #3 and comment #3 of reviewer #1.)

The previous study (now available Schütz et al. 2021, https://doi.org/10.1016/j.ecoleng.2021.106257) was not related to our study. It was designed to investigate the influence of auxiliary water velocity coming from the screen on behavior of five species.
We’ve added the word “unrelated” in section 2.1 for clarification.

Powered by Editorial Manager® and ProduXion Manager® from Aries Systems Corporation

We chose two hydraulically different setups (information added) from this study to test the application range of our model/hypotheses. We did not expect the behavior to be this similar (e.g., Kerr 2016 reported low-energy seeking for trout at smaller U=0.40 m/s).
We still found the result interesting, as it expands the common focus of fishways designers on hydraulic stimuli towards vision under certain conditions (possibly low relative flow velocity and visible boundaries).

10. Line 121-147 – How were these patterns chosen and what hypotheses drove these decisions? Their selection seems somewhat random as written. For example, what details informed splitting the channel at a distance of 0.25 m? Does this distance coincide with an observed hydraulic feature or behavior?YesAdded explanation: To capture the most striking spatial behaviors observed (wall and bottom proximity and turns). 0.25 m was chosen arbitrarily to define wall proximity.

11. Line 155 – Omission of the screen bars is not sufficiently detailed in the methods. I understand the reason for not modelling the bars explicitly, but why not model them as a permeable surface to replicate some of the fine-scale turbulence. See Ho et al. (2011).
Ho, J., Coonrod, J., Hanna, L.J., and Mefford, B.W. 2011. Hydrodynamic modelling study of a fish exclusion system for a river diversion. River Res. Applic. 27: 184–192. doi:10.1002/rra.1349.NoWhile it is possible to model the screen with bars as a permeable baffle, we avoided the effort of implementation and calibration, as we judged the flow field to be sufficiently close to ADV measurements with respect to our behavioral data accuracy. See also reference Gisen 2018, p. 33ff., for detailed comparisons.

Regarding fine-scale turbulence, we would expect some kind of damping effect from a permeable baffle, which would require extra attention.155

12. Line 172 – Again, what underlying hypothesis drove the selection of just these 3 hydraulic variables?YesSee response to comment #1 of reviewer #1.

13. Line 200 – Was distance to the floor included in the wall distance evaluations?NoNo, it wasn't, as the vertical behavior was treated as a separate behavioral rule, which relied on the vertical coordinate (~ pressure) as a stimulus.

14. Since the behavioral rules and selection of stimuli are the main contributions of this work, I found the model description provided in the main text to be underwhelming. I generally understand the adherence to the ODD protocol, but this should not sacrifice the completeness of the main text to act as a standalone document.YesWe've added information on motivation and fatigue, the basic internal states of the model and added a reference (Castro-Santos 2004) to substantiate the importance of repeated passage attempts in the model description.

Results
15. Table 1 – The general magnitude and relation of patterns appears to be nearly identical between set-ups. I would even doubt there is any statistical difference between values. This would indicate one of two possible failures in the experimental design: 1). The modifications to the laboratory set-up did not achieve a discernable change in behavior; or 2). The patterns do not adequately capture the behavioral changes caused by the modification to the set-up. Either way, since the authors do not provide any rationale as to what the set-up change was indented to do makes interpretation difficult.No(see also response #9)
We agree that our trout behavior as measured by the patterns is very similar between setups. This would be your case (1).
However, the previous, unrelated study (Schütz et al. 2021) did not aim to produce different behavior, but was intentionally limited to a typical maximum slot velocity in a multi-species fishway (1.5 m/s). We've added this information in section 2.1.
This limitation coincides with the goal of the present study to provide behavioral rules for fishway design. Hence, the only goal of using two hydraulically different setups was to quantify the (minimum) application range of our model. Finding the maximum application range would be interesting, but less relevant to design of multi-species fishways, which also need to address species with lesser swimming abilities than trout.

Powered by Editorial Manager® and ProduXion Manager® from Aries Systems Corporation

16. Figure 6 – This figure was very helpful to understand the model results and laboratory data. Additional versions of this figure to compare the modelled movement against observed movement would be beneficial. Illustrations from both set-ups should be included.NoThis figure was intended primarily to foster qualitative understanding. It has little explanatory power, as it depicts only two selected observed and two selected model tracks. Showing more tracks like this would not add quantitative information. Also, a figure showing results for setup 2 would add little additional information (as behavior was not much different). Hence, we would prefer to leave the figure unchanged.

Discussion

17. Considering that the observed movement patterns were not largely different between the two set-ups, the conclusion that velocity does not play a role in orientation and navigation is stated too strongly. Based on the available data, it would appear that fish largely exhibited exploratory behavior and followed the walls because the hydraulics did not require any modified behavior. The authors should at least comment that their rule of avoiding high velocities is rather simple. It would not appear to fit the behavioral data either, since passage required movement through an area of elevated velocity in set-up 1. Perhaps a more refined guidance rule surrounding velocity fields is necessary.NoBy no means we intend to state that velocity does not play are role in orientation, generally. However, we found that in our conditions, which are typical for large multi-species fishways built in Germany, brown trout did not use velocity for orientation (probably because it was not demanding enough). We've added explanations to the discussion to clarify this distinction.

Reviewer #3 is right in the point that orientation towards reduced velocity hinders passage through the jet region and slot in setup 1. It is not impossible, however, because the motivation/fatigue driver for movement is independent of the orientation stimulus and there is also a random component to orientation. Further, it cannot be the single cause for the poor performance of the velocity stimulus version in setup 1, as pattern P5 (which measures slot passage) only contributes 20% to the RMSE metric (section 2.5.1).

Still, more refined behavioral rules allowing adaptation to changing flow fields are definitely worthwhile for future work, as discussed in section 4.2.

18. Line 530-532 – The rationale for not running the model with combinations of stimuli is lacking. I can understand the reason for not including this step in this study, but to dismiss their validity is premature. The authors do not explicitly indicate how multiple stimuli could be integrated into a single model, so I do not understand how they can claim the acceleration and velocity versions could not improve upon the wall distance version without explicitly testing this.YesOur statement was based on comparing the 10th percentile/rank results of the stimulus versions under the assumption of using only one stimulus per setup. It is true that we did not test using multiple stimuli within one setup. However, we argue that velocity and acceleration are not relevant for brown trout orientation in our flume conditions (see comment and response #17), therefore we would expect similar results from a model using both stimuli in the same setup. We rephrased the relevant lines to clarify this point.

19. Line 589 – The authors repeatedly indicate that wall perception could be accomplished by vision or audition, but it is not entirely clear how or what role audition may play in wall distance perception. I would still expect near field hydrodynamics would override any true acoustical input for most fish. This just strikes me as an odd statement and should be further explained if it remains in the manuscript.YesWe agree that this is a hypothetic stimulus on the scales investigated here and removed the word "acoustic" in the abstract and conclusions to make that clearer.

Appendix S4

20. Page 4, Para. 1 – The authors state that the relationship between fatigue and swimming speed is not well understood. I find this statement to be highly inaccurate. Numerous studies are available that examine brown trout (and similar salmonids) swimming performance, that is their swim speed vs. duration curves. The authors also site work by Castro-Santos et al. (2005 and 2013) that shows how swimming fatigue could be modelled and swimming performance data for brown trout.YesAgreed, we

	deleted this statement.<br><br>21. Page 8, Table 3 – How was Ug estimated? Castro-Santos et al. (2013) clearly shows laboratory data on brown trout indicated an optimal ground speed of 5.65 BL/s.YesWe estimated the value based on Castro-Santos' 2005 result for striped bass (2.56 BL/s) as stated. We agree that their 2013 paper is a better reference as it deals directly with brown trout. However, note that their value of 5.65 BL/s is a theoretical value for prolonged swim speed and most individuals chose to swim "well below" it (p. 287). Hence, our estimation seems to be in a realistic order. We changed the reference in Table 3, footnote 2, as well as the rationale in section 7.5 accordingly.<br><br>22. Page 10, Section 7.4 – Energy usage is cumulative at the time scale of this study. Once fish expend energy to swim above their sustained swim speed, they are unlikely to recover within 60 mins. However, since the authors assume no fish became fully exhausted from swimming in the flume, their handling of fatigue as a behavioral metric is understandable. This caveat could be clearer in the main text.YesWe did not intend to model fatigue and recovery processes in detail, but just enough to drive model movement as measured by the patterns to enable orientation analyses.<br><br>We've mentioned the caveat in section 2.4, and included references to Castro-Santos et al. 2013 and Ebel 2014.<br><br>23. Page 6, Section 4.7 – The authors should comment on whether the random effects encompass stochasticity in animal behavior and the hydraulic stochasticity (i.e., the real flow field is not static).YesWe've added the explanation that our behavioral model's stochasticity also covers transient flow field effects on the behavior.S4 section 4.7
**Additional Information:**	
**Question**	**Response**
**Financial Disclosure**<br><br>Enter a financial disclosure statement that describes the sources of funding for the work included in this submission. Review the submission guidelines for detailed requirements. View published research articles from *PLOS ONE* for specific examples.<br><br>This statement is required for submission and **will appear in the published article** if the submission is accepted. Please make sure it is accurate.	This work was funded by the German Federal Ministry of Transport and Digital Infrastructure (BMVI, https://www.bmvi.de). The funders had no role in study design, data collection and analysis, decision to publish, or preparation of the manuscript.

<table>
<tr>
<td>
Unfunded studies
Enter: The author(s) received no specific funding for this work.

Funded studies
Enter a statement with the following details:
• Initials of the authors who received each award
• Grant numbers awarded to each author
• The full name of each funder
• URL of each funder website
• Did the sponsors or funders play any role in the study design, data collection and analysis, decision to publish, or preparation of the manuscript?
• NO - Include this sentence at the end of your statement: The funders had no role in study design, data collection and analysis, decision to publish, or preparation of the manuscript.
• YES - Specify the role(s) played.

* typeset
</td>
<td></td>
</tr>
<tr>
<td>
Competing Interests

Use the instructions below to enter a competing interest statement for this submission. On behalf of all authors, disclose any competing interests that could be perceived to bias this work—acknowledging all financial support and any other relevant financial or non-financial competing interests.

This statement is required for submission and will appear in the published article if the submission is accepted. Please make sure it is accurate and that any funding sources listed in your Funding Information later in the submission form are also declared in your Financial Disclosure statement.

View published research articles from PLOS ONE for specific examples.
</td>
<td>The authors have declared that no competing interests exist.</td>
</tr>
</table>

Powered by Editorial Manager® and ProduXion Manager® from Aries Systems Corporation

**NO authors have competing interests**<br><br>Enter: *The authors have declared that no competing interests exist.*<br><br>**Authors with competing interests**<br><br>Enter competing interest details beginning with this statement:<br><br>*I have read the journal's policy and the authors of this manuscript have the following competing interests: [insert competing interests here]*<br><br>* typeset	
**Ethics Statement**<br><br>Enter an ethics statement for this submission. This statement is required if the study involved:<br><br>• Human participants<br>• Human specimens or tissue<br>• Vertebrate animals or cephalopods<br>• Vertebrate embryos or tissues<br>• Field research<br><br>Write "N/A" if the submission does not require an ethics statement.<br><br>General guidance is provided below. Consult the submission guidelines for detailed instructions. **Make sure that all information entered here is included in the Methods section of the manuscript.**	For model development, we used trout observation data obtained by, but not used within, a previous study (Schütz et al. 2021 / DOI:10.1016/j.ecoleng.2021.106257).<br><br>In this study, all care and procedures involving handling and holding fish were conducted as stated and permitted by the district government Karlsruhe (license AZ 35-9185.82/A-6/16).

Powered by Editorial Manager® and ProduXion Manager® from Aries Systems Corporation

**Format for specific study types**<br><br>**Human Subject Research (involving human participants and/or tissue)**<br>• Give the name of the institutional review board or ethics committee that approved the study<br>• Include the approval number and/or a statement indicating approval of this research<br>• Indicate the form of consent obtained (written/oral) or the reason that consent was not obtained (e.g. the data were analyzed anonymously)<br><br>**Animal Research (involving vertebrate animals, embryos or tissues)**<br>• Provide the name of the Institutional Animal Care and Use Committee (IACUC) or other relevant ethics board that reviewed the study protocol, and indicate whether they approved this research or granted a formal waiver of ethical approval<br>• Include an approval number if one was obtained<br>• If the study involved *non-human primates*, add *additional details* about animal welfare and steps taken to ameliorate suffering<br>• If anesthesia, euthanasia, or any kind of animal sacrifice is part of the study, include briefly which substances and/or methods were applied<br><br>**Field Research**<br><br>Include the following details if this study involves the collection of plant, animal, or other materials from a natural setting:<br>• Field permit number<br>• Name of the institution or relevant body that granted permission	
**Data Availability**<br><br>Authors are required to make all data underlying the findings described fully available, without restriction, and from the time of publication. PLOS allows rare exceptions to address legal and ethical concerns. See the PLOS Data Policy and FAQ for detailed information.	Yes - all data are fully available without restriction

A Data Availability Statement describing where the data can be found is required at submission. Your answers to this question constitute the Data Availability Statement and **will be published in the article**, if accepted.<br><br>**Important:** Stating 'data available on request from the author' is not sufficient. If your data are only available upon request, select 'No' for the first question and explain your exceptional situation in the text box.<br><br>Do the authors confirm that all data underlying the findings described in their manuscript are fully available without restriction?	
**Describe where the data may be found in full sentences. If you are copying our sample text, replace any instances of XXX with the appropriate details.**<br><br>• If the data are **held or will be held in a public repository**, include URLs, accession numbers or DOIs. If this information will only be available after acceptance, indicate this by ticking the box below. For example: *All XXX files are available from the XXX database (accession number(s) XXX, XXX.)*.<br>• If the data are all contained **within the manuscript and/or Supporting Information files**, enter the following: *All relevant data are within the manuscript and its Supporting Information files.*<br>• If neither of these applies but you are able to provide **details of access elsewhere**, with or without limitations, please do so. For example:<br><br>*Data cannot be shared publicly because of [XXX]. Data are available from the XXX Institutional Data Access / Ethics Committee (contact via XXX) for researchers who meet the criteria for access to confidential data.*<br><br>*The data underlying the results presented in the study are available from (include the name of the third party*	All relevant data are within the manuscript and its Supporting Information files.<br>All model source code files are available from Github, https://github.com/baw-de/ELAM-flume

Powered by Editorial Manager® and ProduXion Manager® from Aries Systems Corporation

and contact information or URL).<br>• This text is appropriate if the data are owned by a third party and authors do not have permission to share the data.<br><br>* typeset	
Additional data availability information:	

Powered by Editorial Manager® and ProduXion Manager® from Aries Systems Corporation

Manuscript
Click here to access/download;Manuscript;Manuscript.docx

Development of behavioral rules for upstream orientation of fish in confined space

David C. Gisen[1*], Cornelia Schütz[2], Roman B. Weichert[1]

[1] Waterways and environment unit, Bundesanstalt für Wasserbau, Karlsruhe, Germany

[2] Animal ecology unit, Bundesanstalt für Gewässerkunde, Koblenz, Germany

* Corresponding author
E-mail: david.gisen@baw.de (D.C.G.)

Abstract

Improving the effectiveness of fishways requires a better understanding of fish behavior near hydraulic structures, especially of upstream orientation. One of the most promising approaches to this problem is the use of model behavioral rules.

We developed a three-dimensional individual-based model based on observed brown trout (*Salmo trutta fario*) movement in a laboratory flume and tested it against two hydraulically different flume setups. We used the model to examine which of five behavioral rule versions would best explain upstream trout orientation. The versions differed in the stimulus for swim angle selection. The baseline stimulus was positive rheotaxis with a random component. It was supplemented by attraction towards either lower velocity magnitude, constant turbulence kinetic energy, increased flow acceleration, or smaller wall distance.

As expected, modeling rheotaxis was central to matching the laboratory observations. The baseline version was already able to explain large parts of the observed behavior. Mixed results for velocity magnitude, turbulence kinetic energy, and flow acceleration indicated that the brown trout did not orient primarily by means of flow features, probably because the flow field did not challenge them physically.

The wall distance version produced significantly improved results, suggesting that wall distance was the dominant orientation stimulus for brown trout in our hydraulic conditions. The absolute root mean square error (RMSE) was small for the best parameter set (RMSE = 9 for setup 1, RMSE = 6 for setup 2). Our best explanation for this result is dominance of the visual sense favored by absence of challenging hydraulic stimuli. We conclude that under similar conditions (moderate flow and visible walls), wall distance could be a relevant stimulus in confined space, particularly for fishway studies and design in IBMs, laboratory, and the field.

1 Introduction

Worldwide, fishways play a key role in efforts to restore upstream-directed fish migration at dams. The widely differing effectiveness of existing projects (Bunt et al. 2012; Bunt et al. 2016) illustrates that their design is still challenging, especially for species other than Pacific salmon (Kemp 2016). Improving fishway effectiveness for all species requires a better understanding of fish behavior near hydraulic structures, especially of orientation and navigation (Willis 2011; Silva et al. 2018). One of the most promising approaches to better understand such behavior and "a high research priority" (Silva et al. 2018) is the development of behavioral rules. Behavioral rules are the logical and/or algebraical formulation of behavioral hypotheses. They can be implemented and tested in individual-based models (IBMs, Grimm and Railsback 2005).

IBMs allow to implement ~~very closely the~~ concepts of orientation (directional response to local conditions, Willis 2011) and navigation (movement towards a goal outside the local sensory environment, Willis 2011). ~~Typically~~, orientation is ~~the~~ direct outcome of a behavioral rule using a local stimulus, and navigation emerges indirectly from the sum of orientation decisions over time (e.g., Goodwin et al. 2014; Smith et al. 2014; Arenas et al. 2015; Zielinski et al. 2018). Together with their ability to integrate CFD (computational fluid dynamics) model results, IBMs are therefore highly suitable to support fishway design.

A preferred object of research for fishway design are hydraulic stimuli, because they can be manipulated to optimize passage efficiency. The most widely used stimulus in fish orientation IBMs, regardless of whether the model considers upstream or downstream fish migration, is velocity direction (Goodwin et al. 2014; Gao et al. 2016; Tan et al. 2018; Zielinski et al. 2018; Padgett et al. 2020). It is a fundamental stimulus, as it informs positive and negative rheotaxis, which are crucial for guiding upstream and downstream navigation, respectively (Arnold 1974; Elder and Coombs 2015; Coombs et al. 2020). Velocity magnitude is also commonly considered (Arenas et al. 2015; Goodwin et al. 2014; Gao et al. 2016; Tan et al. 2018; Zielinski et al. 2018; Padgett et al. 2020) and especially important for upstream migration, since the energy demand for swimming against the flow is proportional to the cube of relative fish velocity (Wang and Chanson 2018).

Apart from velocity direction and magnitude, stimulus selection to formulate ~~concrete~~ behavioral rules remains difficult (Goodwin et al. 2014). To integrate information on turbulence into IBMs, the turbulence kinetic energy (TKE) is a common choice (Gao et al. 2016; Tan et al. 2018). Turbulence may also be integrated as velocity fluctuation (Zielinski et al. 2018). Other approaches to capture hydraulic information in IBMs include using spatial acceleration (Goodwin et al. 2014; Smith et al. 2014; Arenas et al. 2015) or the strain rate (Tan et al. 2018).

Fish studies from laboratory flumes offer further potential stimuli for modelling upstream orientation, for example the horizontal Reynolds shear stress (τ_{xy}), turbulence intensity, and eddy orientation and scale (see Lacey et al. 2012 for a framework). However, results are manifold and could depend on the specific setup and/or species tested. In different studies, dace preferred adapted

turbulence levels (TKE and τ_{xy}) (Goettel et al. 2015), adult Iberian barbels primarily avoided areas of high τ_{xy} (Silva et al. 2012), and brown trout preferred low drag areas (Kerr et al. 2016). Eddy orientation and scale did influence Iberian barbel behavior (Silva et al. 2012), but are still difficult to define in common vertical-slot fishways (Roth et al. 2021) and are not used in IBMs to date. As for IBMs, the selection of hydraulic stimuli for laboratory studies remains controversial (Kerr et al. 2016).

Non-hydraulic external stimuli for fish orientation, such as visual, olfactory, acoustic, thermal, and magnetic stimuli, are rarely investigated in flows that are comparable to a fishway flow and the authors are not aware of corresponding fish orientation IBMs that include such stimuli. This is particularly notable for vision, as it is considered important for orientation (Montgomery et al. 2000; Liao 2007). Published laboratory and field studies on upstream orientation using visual stimuli as explanatory variable are limited to general phototaxis, i.e. attraction or repulsion (Elder and Coombs 2015; Jones et al. 2017).

For successful upstream migration, a basic motivation to swim against the flow is crucial. However, this motivation may vary widely between species and even between individuals (Kemp 2016). Existing upstream orientation IBMs avoid ~~to model~~ motivation as an internal state by determining that fish constantly try to move upstream (Smith et al. 2014; Gao et al. 2016; Tan et al. 2018; Zielinski et al. 2018; Padgett et al. 2020). However, differing internal states are necessary to produce differing behavioral answers to identical environmental situations. For example, this approach prevents the potentially important ability to drift, recover, and repeat passage attempts after initial failure (Castro-Santos 2004).

Furthermore, existing IBMs for upstream migration suffer from a lack of comprehensive quantitative testing as available for IBMs for downstream migration (Goodwin et al. 2014; Arenas et al. 2015).

In the present study, we thus developed a new IBM for fish upstream orientation and navigation in confined space to support fishway design. For the first time, such a model was tested against detailed data of fish (brown trout) movement in a laboratory flume, and allowed holding behavior, recovery, and thus repeated passage attempts. Five external stimuli were selected for testing based on the literature:

- positive rheotaxis as a fundamental behavior in upstream migrating fish,
- velocity magnitude as being fundamentally related to energy demand,
- TKE as one of the most common and general turbulence variables,
- spatial acceleration as having been tested against the largest data sets by far,
- wall distance as a potential proxy for visual orientation.

The present paper describes how we (1) defined movement patterns from brown trout flume data, (2) developed and tested a CFD model to generate stimuli data, (3) developed and tested a behavioral model to reproduce the movement patterns, and (4) examined which of the five external stimuli would best explain upstream brown trout orientation and navigation in confined space.

2 Methods

2.1 Flume setups

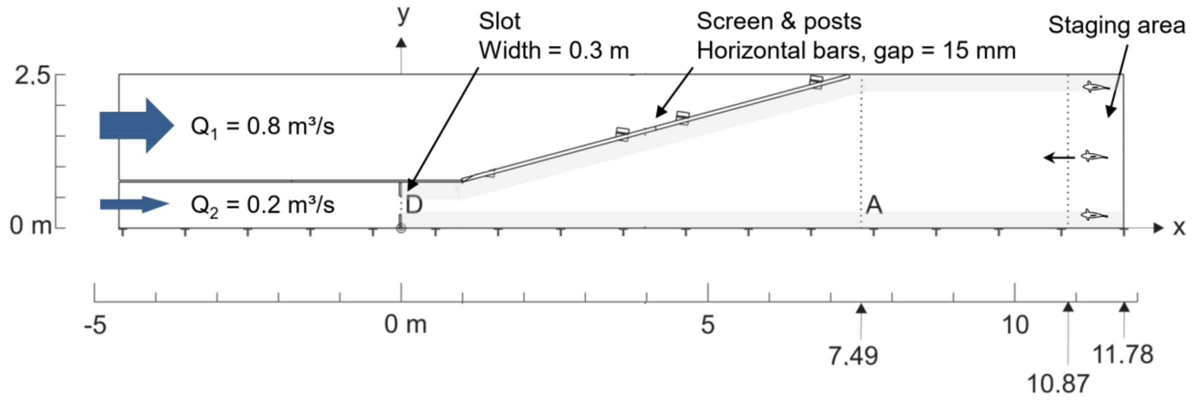

Figure 1: Plan view of flume setup 1. Total length and width = 16.78 m x 2.50 m, water depth = 0.60 m. Flow direction was from left to right. The slot was removed in setup 2 to alter the flow field. Transparent gray areas mark left and right zones (in flow direction) for pattern P1 (defined below, section 2.2). *A* and *D* mark control lines to filter inactive fish and to define pattern P5.

For developing and testing the behavioral model, we used unpublished brown trout movement data from a previous, unrelated study (wild *Salmo trutta fario,* n = 66, mean body length ± standard deviation (SD) = 0.27±0.04 m, Schütz et al. 2021). Fish were caught in close-by rivers during their spawning period in October/November, when an increased motivation to migrate can be expected (Schütz et al. 2021). The study was conducted in a wide indoor flume (Figure 1). The external walls and bottom consisted of smooth acryl glass and metal and the internal walls consisted of coated wood. Water temperature was 16.2-18.2 °C. Water was clear and evenly illuminated. All care and procedures involving handling and holding fish were conducted as stated and permitted by the district government Karlsruhe (license AZ 35-9185.82/A-6/16).

We use two hydraulically different setups from Schütz et al. (2021) to test the application range of our model. The first setup included a jet created by a slot; the second setup had no slot and consequently, no jet. The total discharge ~~was~~ Q = 1.00 m^3/s in both setups ~~to produce~~ a jet velocity of U = 1.5 m/s, which is typical for slots of large multi-species fishways in Germany. The flume was wide enough to enable distinct lateral movement, but narrow enough to influence behavior through the walls and screen.

Fish were released unmarked in batches of three at the downstream end (staging area in Figure 1) and were used only once to avoid learning effects. Fish positions were manually recorded in real time independently by two biologists upon notable change. A change of position was defined as either:

- crossing a meter line of the flume in longitudinal direction
- moving between three zones in lateral direction: close to left wall, close to right wall, far from wall (close meaning max. ~ 0.25 m)
- moving between three zones in vertical direction (close to bottom, close to surface, in between (close meaning max. ~ 0.15 m)

Moreover, it was recorded if fish swam alone or in groups of two or three. Records were attributed to the current test minute. During postprocessing, multiple position changes within the same minute were distributed evenly over the seconds (appendices S1 and S2). Data were verified qualitatively using video records.

To ensure that all fish were actually motivated to move during the experiment, only fish that crossed line *A* (Figure 1) within 30 min after removing the staging mesh were considered valid. A trial was finished either after all three fish passed line *D* or 60 min after the first crossing of line *A*. Data points following a passage of line *D* were excluded. After filtering, we obtained n = 25 tracks for setup 1 and n = 24 tracks for setup 2.

2.2 Movement patterns

We processed the trout track data using *patterns* in the sense of pattern-oriented modeling (Grimm and Railsback 2012) using MATLAB R2018b. We defined five patterns, P1-P5, to capture the most striking spatial behaviors observed during the original experiment. They included a preference for wall and bottom proximity as well as frequently swimming back and forth (turns):

P1, lateral distribution: The flume was divided into a left, middle, and right zone (looking downstream, Figure 1). The dividing lines were located at a lateral distance of Δy = 0.25 m (10 % flume width) to one of either side walls or the screen. Time spent by single fish (i.e., center of mass) was summed for each zone. To account for the differences in total track duration (see Rodríguez et al. 2015), resulting time sums per fish and zone, $t_{zone,i}$, were divided by the particular fish's track duration, $t_{s,i}$, to get relative track fractions, s_{zone}. Finally, s_{zone} was averaged by the fish count, n, to get:

$$\bar{s}_{zone} = \frac{1}{n}\sum_{i=1}^{n}\frac{t_{zone,i}}{t_{s,i}} * 100\ [\%] \qquad \text{(Eq. 1)}$$

where i was a fish iterator. The resulting three lateral zone averages constituted pattern P1. As it is the most direct outcome of upstream orientation, matching it was a major goal of model development.

P2, vertical distribution: Vertically, the flume was divided into a surface, middle, and bottom zone. The dividing lines were located at a vertical distance of Δz = 0.15 m (25 % water depth) to either the water surface or flume bottom, respectively. Tracks were averaged using Eq. 1 to obtain three vertical zone average values. Due to the shallow water, we expected P2 to be of less significance for orientation.

P3, turn zone and *P4, few turns*: Turns were defined as changes in the longitudinal movement direction, x, leading to a displacement $\Delta x \geq 2$ BL before the next direction change. About half of the fish in both data sets performed 4 or less turns. To avoid bias, the data set was divided into turning (> 4 turns, P3, n_{turns}) and few-/no-turning fish (≤ 4 turns, P4, $n_{few\text{-}turns}$). For computing P3, the flume was divided longitudinally into three zones of equal length, in which the relative turn shares were computed and averaged by n_{turns} analog to Eq. 1. P4 was defined as $n_{few\text{-}turns}/n$.

P5, arrival rate: The count of fish navigating the total length of the experimental flume area and crossing line *D*, divided by the count of fish crossing line *A*, formed P5: $n_D/n \cdot 100$ [%].

For use in discussion, we also calculated the lateral distribution of fish at the start position and the proportion of track time fish spent without other fish nearby (i.e., not shoaling).

2.3 Computational fluid dynamics model

A CFD model was employed to calculate the velocity, TKE, and acceleration fields of both setups. We applied the free-surface solver *interFoam* of OpenFOAM 2.3.1 (The OpenFOAM Foundation 2014) to solve the 3D incompressible URANS (unsteady Reynolds-averaged Navier-Stokes) equations.

A hexahedron-dominant unstructured mesh was generated using *snappyHexMesh*. Base resolution was uniform Δ_{xyz} = 5 cm, with local refinement to Δ_{xyz} = 1.25 cm around the slot and screen posts (Figure 1). Horizontal screen bars were omitted, as their influence on the velocity and turbulence field was negligible with respect to behavioral data accuracy (Gisen 2018). Total cell count for setup 1 was 848,694.

Inlet flow rates and outlet fixed water level were set to the laboratory values. For turbulence closure, we chose the k-ω-SST model. At the inlet, TKE ($\triangleq k$) was set to k = 0.001 m^2/s^2 and the specific rate of TKE dissipation was set to ω = 1 Hz. No-slip conditions (velocity vector U = (0,0,0)) and a small sand-equivalent roughness coefficient ($k_S = 1 \cdot 10^{-5}$ m) were set at the walls and bottom.

The fields of velocity U and water/air distribution α_{water} were initialized with a largely converged solution and ran for 80 s of simulated time. U was averaged over the final 20 s to obtain the steady flow velocity vector field $\vec{U}_m$. $\vec{U}_m$ can be expressed either as three-component vector (u,v,w) or as magnitude U_m along with its horizontal and vertical angles γ_m and β_m.

TKE is defined as half the sum of the velocity components' variances (squared turbulence intensities):

$$TKE = \frac{1}{2}\overline{U_i'U_i'} = \frac{1}{2}\left(\overline{u'^2} + \overline{v'^2} + \overline{w'^2}\right) \quad \left[\frac{m^2}{s^2} = \frac{J}{kg}\right] \qquad \text{(Eq. 2)}$$

using Reynolds decomposition, $U_i = \overline{U_i} + U_i'$.

Spatial acceleration a is caused by changes in the flume cross-section and by friction, in contrast to temporal acceleration caused by transient processes. Spatial acceleration is the product of velocity and velocity gradient (which comprises strain rate and rotation rate). In 3D Cartesian coordinates, it reads

$$a = \begin{pmatrix} a_x \\ a_y \\ a_z \end{pmatrix} = (U_m^T \, grad \, U_m)^T = \left(\begin{pmatrix} u & v & w \end{pmatrix} \begin{pmatrix} \frac{\partial u}{\partial x} & \frac{\partial v}{\partial x} & \frac{\partial w}{\partial x} \\ \frac{\partial u}{\partial y} & \frac{\partial v}{\partial y} & \frac{\partial w}{\partial y} \\ \frac{\partial u}{\partial z} & \frac{\partial v}{\partial z} & \frac{\partial w}{\partial z} \end{pmatrix} \right)^T \quad \left[\frac{m}{s^2}\right] \qquad \text{(Eq. 3)}$$

where the superscript ^{T} denotes transposition from column to row vector. Its magnitude $|a|$, U_m, and TKE were used as hydraulic stimuli variables in the behavioral model.

2.4 Behavioral model description

A complete, detailed model description, following the ODD (overview, design concepts, details) protocol (Grimm et al. 2006; Grimm et al. 2020) can be found in appendix S4. Here, we provide a summary. The overall purpose of our model was to find behavioral rules for fish ~~to e.g.~~ enable predictions of fishway attraction and passage efficiency in the long-term. Specifically, we addressed the following question: How well do different external stimuli explain orientation and navigation of upstream moving brown trout?

The entities of the model were fish and mesh cells. Fish were represented as spatially explicit points in 3D space. Their key state variables were position, motivation, and fatigue. Fish movement was modeled kinematically, i.e. there was no influence of fish on the flow field. Key state variables of the mesh cells were the cell label number and the hydraulic variables $|a|$, U_m, TKE, and α_{water}. Spatially, the behavioral model covered the fish-accessible areas of the CFD model (i.e. without the air phase and without areas upstream of the screen). The spatial resolution was that of the CFD model, i.e. 5 cm or smaller. The temporal extent was 60 min of clock time, equal to the maximum laboratory trial duration, divided into constant time steps of Δt = 0.5 s.

Each model run used n_{model} = 100 fish of BL = 0.27 m (as measured), each being assigned to either a "fast" or "slow" category. In this way we tried to model individual traits which could influence migratory tendencies, such as being bold or shy (Chapman et al. 2011) or being physically strong or weak. Model fish were positioned only in the left and right zones of the staging area (Figure 1) as per observations. Their exact distribution was controlled by a parameter.

Every time step, the model computed a new position for every fish. First, a sensory ovoid consisting of seven points around the fish center was determined (Goodwin et al. 2014). They were placed approximately at the skin/water interface, where water motions are detected by the lateral line system (Bleckmann and Zelick 2009). Hydraulic variables were interpolated to these points. Wall distances were computed towards the front and sides.

Next, we aimed to balance driving and retaining traits for upstream and downstream movement, summarized by the variables motivation (to swim upstream), M, and fatigue, F (both range 0.0–1.0). They resembled the typical internal states "need" and "cost" (Willis 2011). We chose this basic representation of internal stimuli because our focus was on orientation and external stimuli. Still, it enabled model fish to drift, recover, and swim upstream again, a behavior which we consider crucial for modeling fish migration through fishways (Castro-Santos 2004). Its success was measured foremost by patterns P3 and P4.

M was increased proportionally ~~to~~ time ~~as long as~~ the model fish made no spatial progress, presuming a principle motivation to move upstream. The maximum increase rate was reached after $k_{M,f}$ = 20 s for "fast" fish and $k_{M,s}$ = 130 s for "slow" fish (default values, varied later). F was computed proportionally to local flow velocity and swim speed. It reached the maximum value at a burst speed of k_F = 25 BL/s (Castro-Santos et al. 2013). We note that more realistic speed-fatigue relations are available (Castro-Santos et al.

2013; Ebel 2014), but they were not required for our purpose of modeling orientation. From the balance of time-averaged *M* and *F*, one of three horizontal behavioral rules was selected (Figure 2):

- migrating (moving upstream),
- holding (maintaining position),
- drifting (moving downstream while allowing random retardation and/or inclination towards the flow).

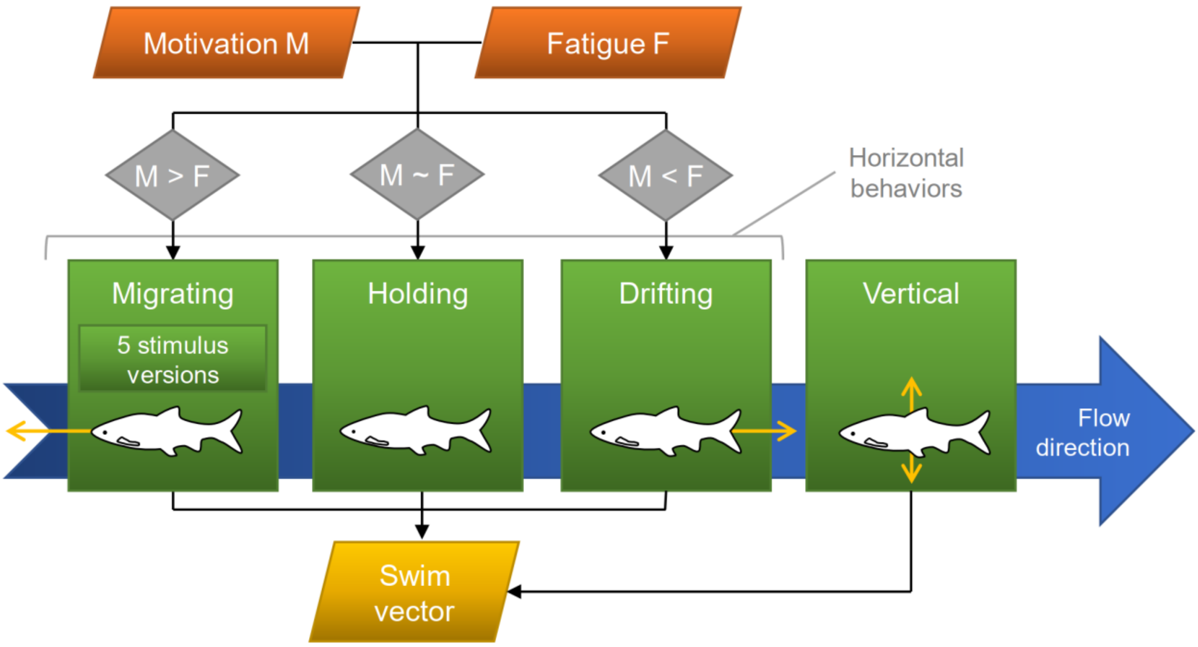

Figure 2: The process of computing the swim vector: Based on the balance between motivation and fatigue, a horizontal behavioral rule is selected. *Migrating* is the key behavioral rule for which five versions are contrasted. *Drifting* behavior includes swimming against, but slower than the flow. In combination with the vertical behavioral rule, the final swim vector is determined.

Migrating was the key behavioral rule. In its baseline version it only depended on the velocity direction. It set the horizontal and vertical swim angles against the velocity direction, as we judged rheotaxis to be a fundamental behavior in fish that is always active (e.g. in Mexican tetra, Elder and Coombs 2015). To also capture smaller, unpredictable behavior variations, a limited random angle was added to or subtracted from the horizontal swim angle. Migrating was expected to have the most direct effect on pattern P1.

Our guiding question ("how well do external stimuli explain upstream orientation?") was addressed by adding four different stimuli for orientation to the baseline migrating behavioral rule. That is, the horizontal swim angle could be adjusted in either one of five alternative stimulus versions:

- Baseline (rheotaxis): Against velocity direction & towards random side by random magnitude as described above.
- Velocity: Baseline & towards side of lower velocity magnitude.
  Rationale: Avoiding zones of high velocity to save energy (low-velocity seeking) is a common strategy for fish and was observed e.g. in laboratory flumes for longnose dace (Dockery et al.

2017) and brown trout (Kerr et al. 2016) and modeled in an IBM for carp and sturgeon (Zielinski et al. 2018).

- TKE: Baseline & towards front or side with the smallest TKE difference towards the fish center (constant turbulence level).
  Rationale: As it is still unclear how turbulence affects orientation in a specific situation or for a single species and fish size, we chose one of the most common and general 3D turbulence variables, containing the turbulence intensity. Orientation along a constant, adapted turbulence level was observed before in dace (Goettel et al. 2015) and modeled in an IBM (Gao et al. 2016).
- Spatial acceleration: Baseline & towards side of higher $|a|$ magnitude.
  Rationale: Acceleration is the hydraulic stimulus tested against the largest data sets by far, although for downstream migration (Goodwin et al. 2014; Arenas et al. 2015). As it also contains the strain rate (Tan et al. 2018) within the velocity gradient tensor, it is an information-rich representation of the flow field and we consider it potentially useful also for upstream migration (e.g., Smith et al. 2014). We chose attraction towards higher acceleration to mimic our laboratory results.
- Wall distance: Baseline & towards side of smaller wall distance.
  Rationale: Within the confined space of our model, wall distance can serve as a potential proxy for the little investigated, but potentially important (Montgomery et al. 2000; Liao 2007) visual orientation. Again, we chose attraction towards smaller wall distance to mimic our laboratory results.

An independent vertical behavioral rule was always active. It limited vertical movement using an elevation difference (Goodwin et al. 2014) and was primarily gauged by pattern P2.

The output of the process described was a 3D swim vector for each fish and time step. The new position was determined from this swim vector, the flow vector at the fish's position, and the time step width. Overall navigational success was measured by pattern P5.

2.4.1 Code and speed

The code of the behavior model and its software framework can be found in appendix S5. The software framework was based on the Fortran 90 code for downstream migrating smolts used in Goodwin et al. (2014). It accounted for e.g. variable storage, sensory point creation, vector transformation from/to Cartesian coordinates, and pseudo-random number creation. We modified it to work with unstructured, polyhedral meshes in OpenFOAM 4.1 using C++. Computation time varied depending on how fast fish exited the domain. A typical runtime for a single model run with n_{model} = 100 fish was about 85 s, using 8.1 GB RAM for up to 7200 time steps on one core of an Intel E5-2660-v3 ("Haswell") CPU.

2.5 Model function test

Besides behavioral rules, parameters (i.e., variables which do not change during a model run, but can change between model runs) are the second main component of a behavioral model. Our methods for testing their influence are described in this section before returning to our actual purpose of testing different stimuli in section 2.6.

2.5.1 Evaluation metric RMSE

To rate the quality of each model run, we computed pattern values P1-P5 in analogy to the laboratory patterns from n_{model} = 100 fish. As evaluation metric, we computed a weighted root-mean-square error (RMSE) between all model and laboratory pattern mean values (Table 1 below) for each model run as

$$RMSE_{model-run} = \sqrt{a_1 \Delta P_1 + a_2 \Delta P_2 + a_3 \Delta P_3 + a_4 \Delta P_4 + a_5 \Delta P_5} \quad [-] \qquad \text{(Eq. 4)}$$

Average of pattern P1-P3: $\Delta P_i = \frac{1}{3} \sum_j \left(P_{i,j,model} - P_{i,j,lab} \right)^2$

Pattern P4-P5: $\Delta P_i = \left(P_{i,model} - P_{i,lab} \right)^2$

where a was a pattern weight factor chosen per pattern importance (a_1 = 0.3, a_2 = 0.1, $a_{3\text{-}5}$ = 0.2), i = 1..5 was a pattern iterator, and j = 1..3 was a pattern value iterator. As P1-P3 consisted of three pattern values each, these were averaged. A value of RMSE = 0 implies perfect agreement between model and laboratory results. We chose RMSE as it is both simple and penalizes large differences, which makes it useful for filtering outliers and achieving an overall good agreement.

The different weights of patterns P1 and P2 reflected their expected importance for modeling orientation (section 2.2). We also tested equal weighting per pattern average value and equal weighting per pattern value, but obtained negligible effects on the stimulus version ranking. We focused on comparing the mean instead of SD values to reduce the complexity of model development, accepting that SD agreement cannot be evaluated.

2.5.2 Random seed sensitivity

The behavioral rules used pseudo-random numbers which were generated from a random seed number fixed for each model run. For identical seeds, results were identical. To minimize and to quantify influence of the random seed choice, we tested all parameter sets with N_r = 10 different seeds. Then, we averaged the resulting $\text{RMSE}_{\text{model-run}}$ values to obtain one RMSE value per parameter set. As seeds, we obtained ten true random numbers from www.random.org in the range from 1-999: 656, 36, 849, 934, 679, 758, 743, 392, 655, 171.

2.5.3 Parameter set generation

In total, the model had 7 fixed and $D = 20$ variable parameters (Table 2 in results and Table 3 in appendix S4, p. 8). The latter could be split into $D_1 = 15$ parameters for general movement, $D_2 = 3$ parameters for initialization, and $D_3 = 1$-2 parameters depending on the chosen stimulus version.

For sensitivity testing and stimulus version testing, we needed to generate a number of parameter sets with systematic variation in their parameter values. To ensure comparability and to save computational effort, we first included all parameters of the five stimulus versions and subsequently filtered the resulting parameter sets as required (Figure 3).

Values of an initial default parameter set were established by trial-and-error. For variation, we chose another 5 values in equal distances around the default value to test a total of $p = 6$ values for each parameter. The default values were either at the 3rd or 4th place of the range (appendix S4).

The full parameter space would comprise p^D parameter sets. We sampled it using the revised Morris method (Campolongo et al. 2007, $r = 1000$ random trajectories, $T = 50$ optimum trajectories) implemented in Python 3.7.4 with SALib 1.3.8. We obtained $n_{sets,base} = (D+1) \cdot T = 1050$ systematically varying base parameter sets consisting of D parameter values each.

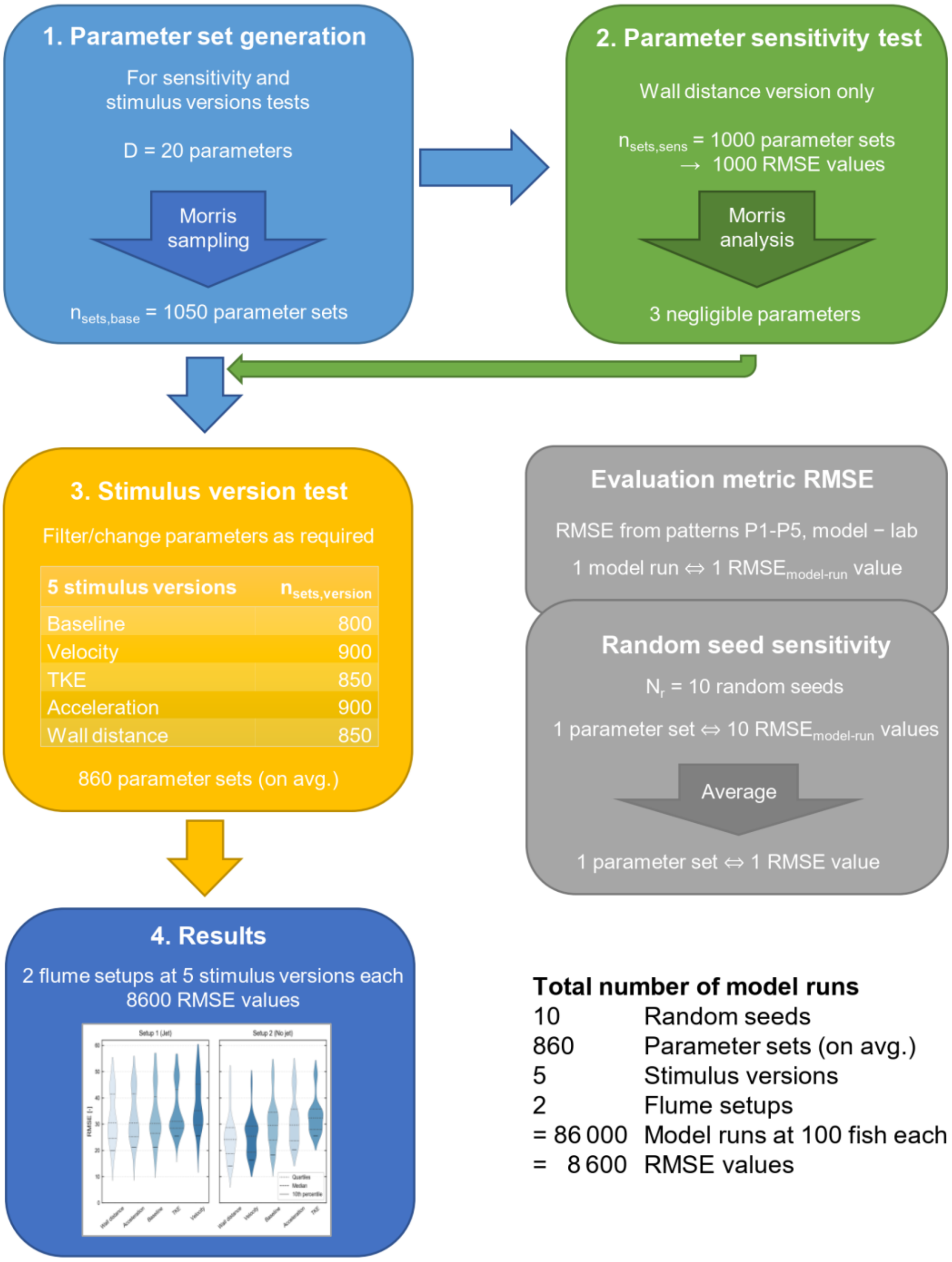

Figure 3: The process of testing our behavioral model. It starts with the generation of varying parameter sets, which are used for testing parameter sensitivity and contrasting stimulus versions in the following. Each box corresponds to one text section.

2.5.4 Parameter sensitivity test

To identify parameters of strong and negligible influence, we analyzed parameter sensitivity. As stimulus version for this investigation, we arbitrarily chose the wall distance in both flume setups. Thus, the acceleration/TKE stimulus threshold parameter was not required here. This reduced the number of parameter sets by T variations to $n_{sets,sens}$ = 1000 parameter sets. As described above, for each parameter set we ran the model with N_r random seeds and averaged the resulting RMSE$_{\text{model-run}}$ values.

Results were analyzed by means of revised Morris screening (Campolongo et al. 2007; Thiele et al. 2014). This method uses the effect of single parameter variations on the averaged RMSE to determine an influence measure, μ^*, and an interaction measure, σ_M, for each parameter. We used μ^* for ranking parameters by influence. For further interpretation, the values of the measures needed to be classified as "high" or "low". This is usually achieved graphically (e.g., Campolongo et al. 2007). We took a quantitative approach through normalizing μ^* and σ_M by their respective maximum values and, then, defining limits in dependency of gaps in our results. We neglected three parameters with "low" influence (see results) in the subsequent stimulus version tests.

2.6 Stimulus version test

2.6.1 Parameter influence

To find the stimulus which best explained upstream orientation, we tested five versions of the migrating behavioral rule in both setups. All other behavioral rules of the model (including behavior selection, holding, drifting, and vertical behavior) were kept constant (for a protocol, see Grimm and Railsback 2012). The latter were tested implicitly by means of the overall result, to which they contributed.

To eliminate the risk of favoring a stimulus by a certain calibration/parameter set ("overfitting"), we applied a large number of identical parameter sets to each of the stimulus versions. They were generated from the base parameter sets by filtering the negligible parameters identified in sensitivity analysis. Slight differences in the number of sets, n_{sets}, arose from three stimulus-specific parameters:

- the wall detection range was used only by the wall distance stimulus;
- the threshold factor was used only by the velocity and TKE stimuli;
- the default angle for the wall distance stimulus was half the default angle of the velocity, TKE, and acceleration stimuli.

The procedure yielded an average of $n_{sets,avg}$ = 860 parameter sets per stimulus version (range 800–900, Figure 3). For each parameter set, we ran the model with N_r random seeds and averaged the resulting $RMSE_{model\text{-}run}$ values to obtain one RMSE value. From the combination of parameter sets ($n_{sets,avg}$ = 860), flume setups (n_{setups} = 2), and stimulus versions ($n_{stimuli}$ = 5), we received a total of 8600 RMSE values (Figure 3).

2.6.2 Ranking

Since sampling of the parameter sets is a random process and not optimized towards better performance results, it includes many unfavorable parameter sets that are not suitable to assess stimuli performance. Thus, a selection of the best model runs/RMSE values was necessary to find out if the different stimulus versions performed equally good or differed from each other. Based on the frequency distribution, we sought a threshold where a clear rejuvenation with regard to better values was visible,

while still enough RMSE values for a statistical valid comparison remained. We opted for the 10th percentile of RMSE values, which was then used to rank the stimulus versions.

We treated both setups separately, as setup 1 was more demanding on the model due to the more heterogeneous flow field and the slot geometry.

To evaluate if differences between the stimulus versions were statistically significant, we computed the *p*-values (Kruskal-Wallis test with post-hoc Nemenyi test) between the RMSE results (cut-off above 10th percentile) of the stimulus versions separately for both setups using SciPy 1.0.1 with scikit-posthocs 0.6.6.

Finally, to facilitate vivid understanding and discussion, we classified the versions qualitatively. The best version per setup was classified as "good", which was backed by comparing the RMSE of its best parameter set to absolute reference values. Versions differing significantly ($p < \alpha = 0.01$) from the best version were classified as "moderate" or "poor" under consideration of the 10th percentile. Versions not differing significantly were classified as "good".

3 Results

3.1 Movement patterns

Analysis of the movement patterns provided several insights into observed trout behavior (Table 1). Pattern P1 indicated that brown trout spent the major part of trial time close to the lateral walls and screen, i.e. in a rather small share of the flume width. P2 showed that fish swam close to the bottom almost exclusively. P3-P5 were less distinct. Large SD values indicated wide individual differences in observed behavior for P1 and P3.

Brown trout start positions were distributed to the P1 left/middle/right zones 68 % - 4 % - 28 % (setup 1) and 46 % - 13 % - 42 % (setup 2). They spent an average of about 60 % of track duration without other fish nearby.

Table 1: Mean and standard deviation (SD) of brown trout patterns in the two flume setups.

**Pattern**	**Flume zone**		**Setup 1 (jet)**			**Setup 2 (no jet)**		
	Name (facing downstream)	Extent (m)	*n*	mean (%)	SD (%)	*n*	mean (%)	SD (%)
P1 Lateral distribution	Left	0.25	25	33	±37	24	38	±39
	Middle	2.00	25	10	±12	24	6	±10
	Right	0.25	25	57	±38	24	56	±40
P2 Vertical distribution	Surface	0.15	25	1	±2	24	4	±17
	Middle	0.30	25	1	±2	24	0	±1
	Bottom	0.15	25	99	±3	24	96	±17
P3 Turn zone	Upstream	3.25	9	24	±19	12	27	±17
	Middle	3.25	9	29	±17	12	40	±16
	Downstream	3.25	9	47	±16	12	33	±26
P4 Few turns	-	-	15	60	-	12	50	-
P5 n_D/n	-	-	25	84	-	24	79	-

Values rounded to the closest integer. n_D and n, number of fish at lines *D* and *A*.

3.2 Computational fluid dynamics model

CFD model results were in good agreement with acoustic Doppler velocimeter measurements from the laboratory (Gisen 2018). Advection dominated the flow field; boundary roughness influence was limited to a few centimeters in both setups (Figure 4). Absence of the jet in setup 2 considerably reduced mean velocity U_m, acceleration magnitude, and TKE on the right-hand flume side (facing downstream). The flow field of both setups was asymmetric in lateral direction. The bulk flow velocity was U_{bulk} = Q/(width*water depth) ≈ 0.67 m/s. In large parts of the jet region of setup 1, flow velocity was about U_m ≈ 0.8 m/s.

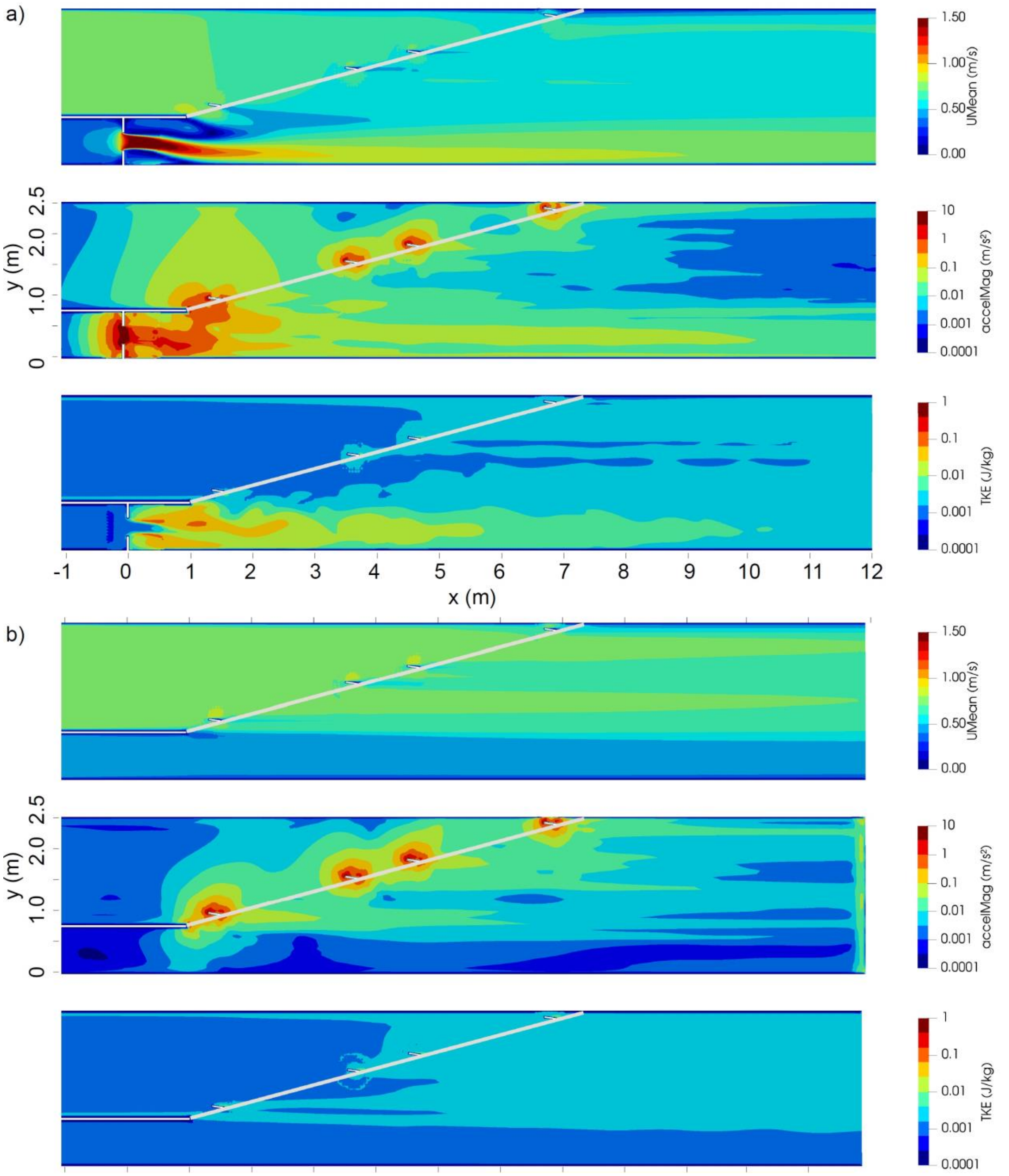

Figure 4: Horizontal flow field slices of setup 1 (a) and setup 2 (b) illustrate the effect of the slot and jet and its removal. Flow from left to right. Plane at z = 0.07 m above bottom where trout preferred to stay. Water depth = 0.60 m. Sub-panels in (a) and (b) show velocity magnitude U_m (top panels), acceleration magnitude (middle panels), and TKE (bottom panels). Note logarithmic scale for acceleration and TKE.

3.3 Random seed and time step sensitivity

To evaluate the influence of the different random seeds used, we compared the median coefficient of variation (σ/μ) for all ten setup/stimulus combinations. The values were low and ranged from 0.02-0.04, suggesting that our results were independent of the random seed chosen.

Halving the default time step to Δt = 0.25 s for the best wall distance parameter set deteriorated the RMSE in setup 1 and improved it in setup 2. Doubling the time step deteriorated the RMSE in both setups.

3.4 Parameter sensitivity

Parameter sensitivity was computed in both setups for the wall distance stimulus only. Setup 1 exhibited seven parameters of high influence ($\mu^*/\mu^*_{max} > 0.6$, Table 2), while setup 2 exhibited four parameters above this arbitrary limit. Ranks 17-19 comprised three parameters of low, negligible, effect on the result ($\mu^*/\mu^*_{max} \leq 0.1$). There was no first-order (independent) parameter (indicated by a high μ^* and low σ_M).

Both swim angles included in the migrating behavioral rule had a high influence and interaction in both setups. This result confirms that modeling orientation was important for the result. The vertical behavior correction angle was distinctly more important in setup 1 than in setup 2. This was likely caused by vertical currents induced by the plunging jet in setup 1.

All parameter sets which differed in one of the negligible parameters were excluded from the subsequent stimulus version tests. The parameter "wall detection range" was bound to the wall distance stimulus, anyway. The other two negligible parameters were not directly related to orientation, i.e. were expected to not interact strongly with the chosen stimulus. For the resulting number of parameter sets per stimulus version, n_{sets}, see Figure 3.

Table 2: Parameters of the behavioral model with wall distance stimulus, ranked by normalized influence, μ^*/μ^*_{max}, in setup 1. $\sigma_M/\sigma_{M,max}$ is normalized interaction with other parameters. The threshold for "low" values is indicated by a horizontal line. The last column, *No.*, is for reference to Table 3 in appendix S4, p. 8.

**Setup 1 (Jet)**			**Parameter name**	**Setup 2 (No jet)**			**No.**
**Rank**	**μ^*/μ^*_{max}**	**$\sigma_M/\sigma_{M,max}$**		**Rank**	**μ^*/μ^*_{max}**	**$\sigma_M/\sigma_{M,max}$**	
1	1.00	1.00	Migrating behavior max. random angle	2	0.74	0.60	14
2	0.82	0.87	Holding behavior extent	5	0.55	0.56	9
3	0.77	0.89	Migrating behavior wall distance angle	4	0.63	0.59	16
4	0.70	0.86	Fatigue (decreasing) memory coefficient	1	1.00	1.00	6
5	0.68	0.72	Vertical behavior correction angle	16	0.12	0.21	12
6	0.67	0.68	Spot memory coefficient	3	0.68	0.89	5
7	0.66	0.79	Motivation denominator ("fast fish")	7	0.47	0.69	3
8	0.56	0.64	Motivation memory coefficient	6	0.47	0.60	2
9	0.47	0.53	Stuck time threshold	10	0.22	0.34	13
10	0.41	0.46	Vertical behavior elevation threshold	13	0.13	0.17	11
11	0.33	0.41	Fatigue denominator	14	0.13	0.19	8
12	0.32	0.37	Motivation denominator ("slow fish")	9	0.27	0.37	4
13	0.24	0.34	Motivation initial value	11	0.21	0.30	1
14	0.24	0.29	Initialization "slow fish right"/"right fish"	15	0.13	0.17	20
15	0.21	0.20	Initialization "slow fish left"/"left fish"	12	0.13	0.34	19
16	0.20	0.23	Initialization "left fish"/"total fish"	8	0.44	0.44	18
17	0.09	0.10	Drifting behavior straight drift probability	17	0.10	0.23	10
18	0.07	0.07	Fatigue (increasing) memory coefficient	18	0.05	0.08	7
19	0.04	0.04	Migrating behavior wall detection range	19	0.02	0.02	15

3.5 Stimulus versions

We contrasted five stimulus versions for upstream swim angle selection in the model and ranked them (Table 3, Figure 5). The Kruskal-Wallis test obtained p = 16.2E-54 for setup 1 and p = 1.1E-78 for setup 2, suggesting that the stimulus versions did influence accordance with the laboratory observations significantly.

Table 3: Stimulus versions ranked by their RMSE 10th percentile, per setup (appendix S6). The p-value was calculated *post hoc* between wall distance and the particular stimulus version. Significant differences between these versions at $p < \alpha$ = 1.0E-02 are denoted by an asterisk (*). p-values are rounded to two significant digits, percentile values are rounded to integer.

Setup	Stimulus version	RMSE 10th percentile	p-Value	Rating
1 - Jet	Wall distance	20	-	good
	Acceleration	21	1.2E-01	good
	Baseline	21	4.0E-01	good
	TKE	25	*4.1E-30	poor
	Velocity	26	*2.7E-27	poor
2 - No jet	Wall distance	14	-	good
	Velocity	16	8.9E-02	good
	Baseline	18	*1.2E-12	moderate
	Acceleration	20	*9.5E-29	moderate
	TKE	26	*2.0E-60	poor

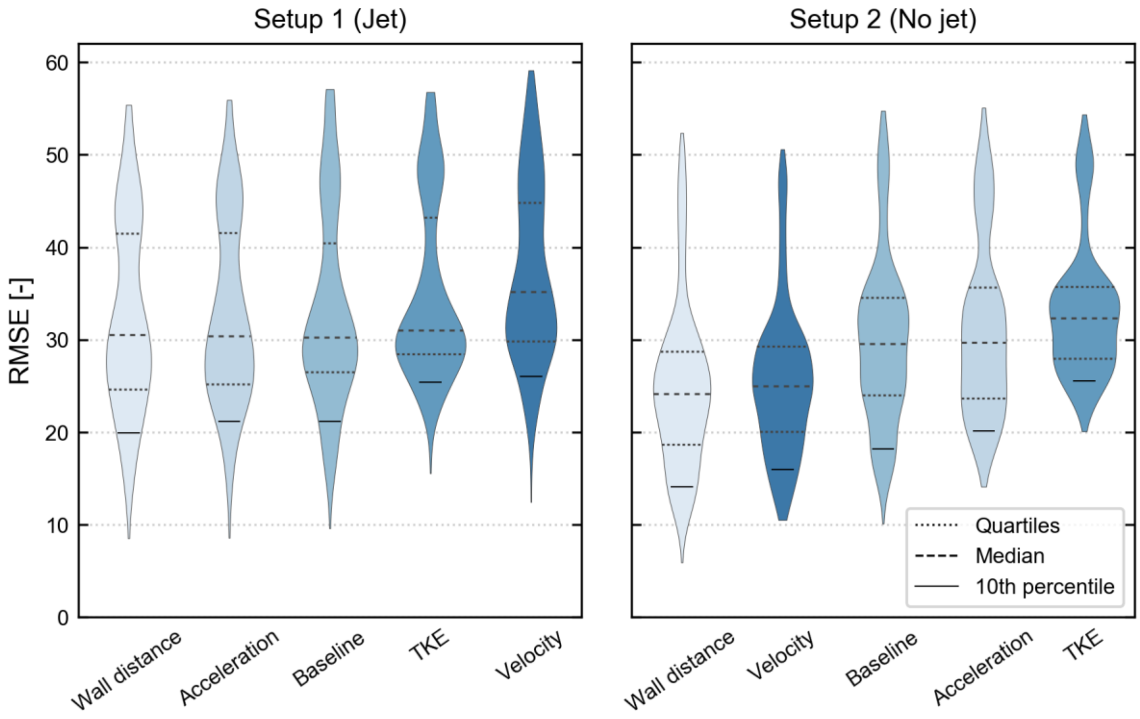

Figure 5: Violin plots indicating the frequency of root-mean-square error values (RMSE) in both setups (appendix S6). Ordering by increasing 10th percentile reveals similarities and significant differences between the stimulus versions. Results are better if more values are close to zero.

The lower violin tip indicates the result of the best parameter set(s) for each stimulus version.

Wall distance was the best stimulus version in both setups. Acceleration and velocity differed widely in their ranks between setups, while baseline and TKE did not. In setup 1, the order of acceleration and baseline was not distinct, as well as the order of TKE and velocity (Figure 5). This is reflected in our qualitative “rating”/classification used for discussion (Table 3). The estimated distributions of the RMSE results are mainly multi-modal, which was the reason to rank them by their 10th percentile. The overall better results in setup 2 indicate that it was less demanding on the model than setup 1.

The wall distance version with its best parameter set reproduced all five patterns with high accuracy (RMSE = 9 for setup 1, RMSE = 6 for setup 2) (Figure 5, Figure 6). For comparison, e.g. Goodwin et al. (2014) aimed for RMSE ≤ 10. This finding shows that our model is able to produce all essential characteristics captured by our patterns (Grimm and Railsback 2005, p. 47).

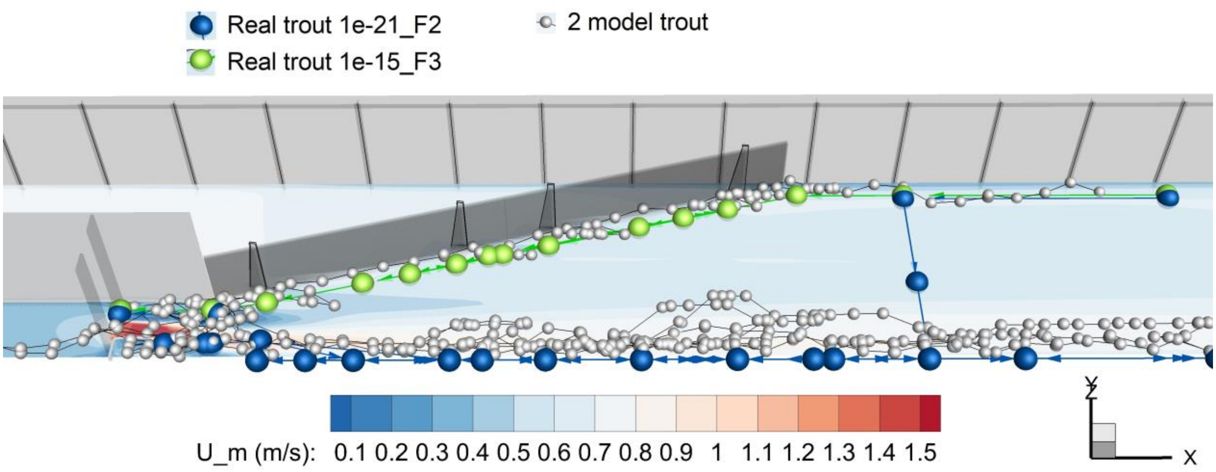

Figure 6: Two selected real and two selected model trout tracks in setup 1 (with jet). Each sphere shows one noted position or model time step, respectively. Model results are from the best parameter set of the wall distance stimulus. Plane shows mean velocity, U_m, at z = 0.07 m above the bottom. Flow direction is from left to right, fish start from right on both flume sides. See appendices S1 and S2 for observed trout movement data.

4 Discussion

4.1 Stimuli for orientation

In this work, we combined the very different fields of ethology, hydraulic engineering, and behavior modeling to approach a classical problem of behavioral ecology: How can orientation behavior be explained through external stimuli? Our resulting movement patterns, flow fields, and significant differences between the model stimulus versions allow to develop explanations with respect to the ambient conditions in our two setups.

Results for the baseline version ranged from moderate in setup 1 to good in setup 2, indicating that rheotactic orientation and a random component may already explain not only orientation (pattern P1),

but also large parts of the remaining observed behavior captured by P2-P5. Positive rheotaxis was expected to be important as both velocity direction and observed movement directions were largely uniform and aligned, fostering this basic behavior(Coombs et al. 2020). Also, this result confirms that the random component was chosen in the right order to cover effects not modeled explicitly. Still, it was unexpected that the other hydraulic variables commonly associated with behavior, i.e. velocity magnitude, TKE, and acceleration (e.g., Dockery et al. 2017; Goettel et al. 2015; Goodwin et al. 2014), were not required for this result. The apparent explanation that the flow field was not challenging enough to influence trout behavior will be discussed along the alternative stimulus versions tested.

The velocity model version yielded poor results in setup 1 and good results in setup 2. One reason for the failure in setup 1 was that model fish mostly avoided the jet of higher velocity on the right-hand side, while observed fish frequented this zone in both setups (P1). The laboratory observations indicate that the tested trout did not aim to minimize their energy consumption by following low-velocity paths upstream as expected (Wang and Chanson 2018; Zielinski et al. 2018). Hence, the good model results in setup 2 are not the result of a real mechanism. They are rather a model effect of the low-velocity zone on the right-hand side, which fostered wall attraction and thus agreement to the observations. There is no apparent reason why trout would not seek lower velocity in setup 1 (with larger bulk velocity) but would do so in setup 2. In conclusion, our model and laboratory results suggest that velocity was not a relevant stimulus for our trout.

To find an explanation, we used the body length as a proxy for swimming ability and considered the velocity/body length relation. A trout (BL = 0.27 m) holding station experienced a relative flow velocity of about U_{fish} = 3.0 BL/s in large parts of the jet in setup 1. At our water temperature, trout can maintain a sustained swimming speed of U_{fish} = 4.5 BL/s for up to 200 min according to Ebel's model equation for the rheophilic guild (Ebel 2014). Thus, in theory, trout could hold or move upstream while experiencing almost no fatigue (Webb 1975) for longer than the test duration, indicating that the flow velocity was indeed too low to trigger a reaction. Some support for this explanation comes from two studies, where low-velocity seeking was observed only at much larger relative velocities. Broadly estimated relative velocities were (0.37 m/s)/(0.07 m/BL) ≈ 5.3 BL/s for Duboulay's rainbowfish (Wang and Chanson 2018) and (0.39 m/s)/(0.06 m/BL) ≈ 6.5 BL/s for longnose dace (Dockery et al. 2017). In contrast, Kerr et al. (2016) reported low-velocity seeking already at (0.40 m/s)/(0.14 m/BL) ≈ 2.9 BL/s for small hatchery brown trout, which is similar to our relative velocity. This discrepancy indicates that a general movement hypothesis has to include more factors than just relative velocity.

Our next test for constant TKE attraction scored poor results in both setups. As both walls and bottom were smooth, they did not provide a distinct TKE stimulus for wall attraction, resulting in large deviations in P1 and the RMSE. These results challenge the statement that TKE is "by far the best stimulus" for fishway models (Gao et al. 2016) and the hypothesis that a constant turbulence level can be employed for orientation (Goettel et al. 2015). We did not test attraction towards increasing or decreasing TKE, but would expect similarly poor results for P1 as the TKE field offers a decent stimulus only towards one side (lateral asymmetry), while the fish observed visited both sides.

Like for velocity, we compared TKE levels with literature values to determine if a relation to body length could explain our results. Our setups exhibited levels of TKE = 0.001-0.1 J/kg. Studies which explained behavior using TKE reported higher values for smaller fish: TKE = 0.1-0.3 J/kg for trout, BL = 0.20 m (Gao et al. 2016) and TKE = 0.015 J/kg for dace, BL = 0.07 m (Goettel et al. 2015). This qualitative comparison is in agreement with the explanation that the flow field did not cause behavioral reactions as it was not challenging for trout. However, TKE is less studied for orientation than velocity and further research is required.

Results of the acceleration model version (good and moderate, respectively) were similar in absolute RMSE terms between setups, but differed widely in their in-setup rank because of better performances of the velocity and baseline versions in setup 2. Finding a theoretical explanation is difficult, as the few existing quantitative studies dealing with acceleration as an orientation stimulus focus on downstream movement (Kemp et al. 2006; Goodwin et al. 2014), which is associated with different responses than upstream movement. As for velocity (but with the better setup swapped), we suspect that a model effect, and not a real mechanism, is responsible for the different ranking: Acceleration amplifies the velocity gradient at the lateral walls and produces a distinct stimulus for model wall attraction in setup 1, but not in setup 2, thus failing to match P1 observations (which are similar in both setups). Again, the most probable explanation for our inconsistent results with acceleration, velocity, and TKE is that the live trout did not orient by means of the flow field (except for direction), but by other factors. The most relevant is non-hydraulic perception of wall distance.

Using the wall distance version, our model performed well in terms of absolute RMSE and significantly better than any alternative version in at least one setup. This result is in line with the assumed limited hydraulic influence on orientation in our conditions. As this model version is based on following a gradient pointing towards the wall, it carries the risk of imposing (Grimm and Railsback 2005) large residence durations near the walls. This risk is mitigated by two mechanisms for moving away from the wall, namely the random overlay angle and inclined drift. The good RMSE results for this version suggest that these mechanisms are in the right balance and that wall attraction by distance was a real behavior. Potential reasons include guidance, cover, or hydraulic advantages from the walls, but further experiments are required to evaluate their probability.

For the physiological mechanism of wall attraction, multiple explanations are conceivable. To us, visual distance estimation is the best explanation, considering that vision is a predominant stimulus in fish (Douglas and Hawryshyn 1990; Liao 2007; Elder and Coombs 2015). Given the clear visibility of the walls, it seems unlikely that another potential far-field stimulus (e.g. acoustic) dominated distance estimation. Distance estimation by means of a hydraulic signature is also unlikely according to our CFD model results: as discussed before, they show that wall influence was limited to some centimeters distance and/or was inconsistent between setups in hydraulic variables (velocity, TKE, acceleration) which are commonly associated with behavioral responses (e.g., Dockery et al. 2017; Goettel et al. 2015; Goodwin et al. 2014). As a study on blind cave fish shows, hydrodynamic imaging by the lateral line close to smooth walls is limited to a small fraction of the body length (Windsor et al. 2010).

Taken together, our results indicate that the rarely considered wall distance can be a relevant stimulus for orientation in fishways and similarly confined migration paths. It requires, of course, ambient conditions which allow its perception, e.g. clear and light water. Further, it may be overruled by hydraulic stimuli if they are relatively larger (in relation to fish swimming ability) than in our conditions.

4.2 Stimulus combinations

In our model, we limited orientation stimuli to the baseline velocity direction and one additional stimulus at a time. This is a simplification of reality, where most complex behavior is unlikely to depend on a single stimulus/sense alone (Liao 2006; Goettel et al. 2015), and is more likely to have a polysensory background (Kasumyan 2011). While making a model more realistic is not the same as making it more useful, combining stimuli to act on the same behavior is a worthwhile research direction for improving the generality of fish orientation IBMs.

A recent example is the use of calibrated weight factors for three hydraulic stimuli in an upstream orientation IBM for carp (Tan et al. 2018). A further step would be to model shifting influence of stimuli depending on the ambient conditions, which was shown to exist in different species (Elder and Coombs 2015; Fuentes-Pérez et al. 2018). However, adapting our model to use e.g. acceleration in setup 1 and velocity in setup 2 would still yield results not as good as the wall distance version (two times rank 2 vs. two times rank 1). In addition, both stimuli were likely not real stimuli (as discussed above) and hence such a model version would reduce robustness and explanatory power for our experimental conditions. Therefore, we decided not to run our model with stimuli combinations.

4.3 Movement patterns in the laboratory

Transfer of our model also depends on how universal the underlying movement patterns are. The observed tendency to swim close to the walls (pattern P1) matched descriptions of rainbow trout behavior (Przybilla et al. 2010) and barbel behavior in a confined model fishway (Fuentes-Pérez et al. 2018). It was not observed in faster flow and a more narrow flume for brook and brown trout (Castro-Santos et al. 2013). This discrepancy indicates a potential dependence on the ambient conditions. For our slow, shallow flow, we conclude that P1 is not an untypical behavior.

Swimming at the bottom is reported frequently, e.g. in a natural stream for brown trout (Cotel et al. 2006), and in flumes for rainbow trout (Przybilla et al. 2010), barbels (Silva et al. 2012), brown and brook trout (Castro-Santos et al. 2013), and longnose dace (Dockery et al. 2017). The water column in our experiment was shallow (d = 0.60 m), hence it was unclear whether fish preferred the bottom for orientation, for shelter, or for another reason. Despite possible behavioral or physiological reasons, P2 can be considered a typical behavior in our flume conditions.

For patterns P3 and P4, we did not find matching descriptions in the literature, because they represent a new way of systematically dealing with the striking feature “turns”. P5 was also too specific to our setup.

From the literature comparisons, it seems plausible that patterns P1 and P2 would be universal enough to support model transfer to similar flows, e.g. to an altered flume geometry. Transfer to the real world could be challenging, e.g. due to limited wall distance estimation and/or larger flow velocities.

4.4 General limitations

Finally, we want to point out some general limitations of this work to facilitate critical reception. First, our results are limited in scale to the estimated fish observation accuracy, which was coarser than the scale of changes in the hydraulic variables in a distance of 1-2 m downstream of the slot. Thus, potential effects of sudden changes in this area on the behavior could not be incorporated into the model.

Second, we found that different parameter combinations produced similarly good results, which indicates that parameter values are not robust and transfer of the behavioral model to other species and/or hydraulic environments requires recalibration. This does not affect our reproducible testing procedure of complex hypotheses, which is one of the major assets of IBMs (Willis 2011).

Third, the behavioral model was sensitive to changes in time step width. Main influences changing with the number of time steps include accuracy of flow field perception (Smith et al. 2014) and the number of orientation and random decisions.

Fourth, matching P1 depended, among other effects, on the initial distribution of fish positions to either side. The share of fish starting on the left flume side in the best wall distance version was off the real distributions observed in the flume (33 % vs. 68 % with jet and 33 % vs. 46 % without jet). Producing matching results from differing initial conditions could point to a difference between model and laboratory behavior or it could be a sign of minor relevance of the initial conditions to the model result.

Fifth, our model is currently limited to steady flow fields. Modeling e.g. utilization of transient vortices would require considerable model modification and computational resources (Padgett et al. 2020).

Sixth and last, although swimming in groups (shoaling) was not pronounced in our data set and not modeled, it can influence orientation behavior. Determining its influence would require laboratory experiments with single fish releases.

5 Conclusions

Our goal in this study was to better understand upstream fish orientation and navigation in confined space, e.g. in fishways, by means of ~~concrete~~ behavioral rules. Our thoroughly tested IBM was able to reproduce five movement patterns of brown trout in two experimental flow fields, which represented a broad range of behavior in a wide flume. The first two patterns – preferring wall and bottom proximity – were very distinct, and should be investigated further to determine their dependency on ~~e.g.~~ water width and depth.

The significant advantage of the wall distance stimulus version illustrates that a focus on hydraulic stimuli for predicting fish orientation can be too narrow, especially in hydraulic conditions not challenging for the observed individuals. These can occur ~~e.g~~. for strong swimmers in multi-species fishways. In such conditions, particularly when the walls are visible, wall distance should be considered more frequently as a stimulus in IBMs, laboratory experiments, and field studies.

The evaluation of observed patterns and key parameter ~~variety~~ indicates that transfer of our present model is likely limited to similar species and conditions, i.e. brown trout of a given size in a relatively slow, shallow flume flow. This does not affect the IBM's ability to contrast different behavior hypotheses.

In summary, the interdisciplinary IBM technique facilitates testing of behavioral rules using alternative stimuli with flexibility and rigor. It is an important option to approach the many urgent questions of fishway designers.

6 Acknowledgments

We thank all persons involved in the realization of the brown trout movement experiments.

We thank Dr. Andy Goodwin of USACE for providing the Fortran 90 model code used in Goodwin et al. (2014) as a starting point.

7 References

Arenas, Antonio; Politano, Marcela; Weber, Larry; Timko, Mark (2015): Analysis of movements and behavior of smolts swimming in hydropower reservoirs. In *Ecological Modelling* 312, pp. 292–307. DOI: 10.1016/j.ecolmodel.2015.05.015.

Arnold, G. P. (1974): Rheotropism in fishes. In *Biological Reviews* 49 (4), pp. 515–576. DOI: 10.1111/j.1469-185X.1974.tb01173.x.

Bleckmann, Horst; Zelick, Randy (2009): Lateral line system of fish. In *Integrative zoology* 4 (1), pp. 13–25. DOI: 10.1111/j.1749-4877.2008.00131.x.

Bunt, C. M.; Castro-Santos, T.; Haro, A. (2016): Reinforcement and Validation of the Analyses and Conclusions Related to Fishway Evaluation Data from Bunt et al.: 'Performance of Fish Passage Structures at Upstream Barriers to Migration'. In *River Res. Applic.* 32 (10), pp. 2125–2137. DOI: 10.1002/rra.3095.

Bunt, Christopher M.; Castro-Santos, Theodore; Haro, Alex (2012): Performance of fish passage structures at upstream barriers to migration. In *River Res. Applic.* 28 (4), pp. 457–478. DOI: 10.1002/rra.1565.

Campolongo, Francesca; Cariboni, Jessica; Saltelli, Andrea (2007): An effective screening design for sensitivity analysis of large models. In *Environmental Modelling & Software* 22 (10), pp. 1509–1518. DOI: 10.1016/j.envsoft.2006.10.004.

Castro-Santos, Theodore (2004): Quantifying the combined effects of attempt rate and swimming capacity on passage through velocity barriers. In *Can. J. Fish. Aquat. Sci.* 61 (9), pp. 1602–1615. DOI: 10.1139/f04-094.

Castro-Santos, Theodore; Sanz-Ronda, Francisco Javier; Ruiz-Legazpi, Jorge; Jonsson, Bror (2013): Breaking the speed limit — comparative sprinting performance of brook trout (Salvelinus fontinalis) and brown trout (Salmo trutta). In *Can. J. Fish. Aquat. Sci.* 70 (2), pp. 280–293. DOI: 10.1139/cjfas-2012-0186.

Chapman, Ben B.; Hulthén, Kaj; Blomqvist, David R.; Hansson, Lars-Anders; Nilsson, Jan-Åke; Brodersen, Jakob et al. (2011): To boldly go. Individual differences in boldness influence migratory tendency. In *Ecology letters* 14 (9), pp. 871–876. DOI: 10.1111/j.1461-0248.2011.01648.x.

Coombs, Sheryl; Bak-Coleman, Joe; Montgomery, John (2020): Rheotaxis revisited. A multi-behavioral and multisensory perspective on how fish orient to flow. In *The Journal of experimental biology* 223 (Pt 23). DOI: 10.1242/jeb.223008.

Cotel, Aline J.; Webb, Paul W.; Tritico, Hans M. (2006): Do Brown Trout Choose Locations with Reduced Turbulence? In *Transactions of the American Fisheries Society* 135 (3), pp. 610–619. DOI: 10.1577/T04-196.1.

Dockery, D. R.; McMahon, T. E.; Kappenman, K. M.; Blank, M. (2017): Evaluation of swimming performance for fish passage of longnose dace Rhinichthys cataractae using an experimental flume. In *Journal of fish biology* 90 (3), pp. 980–1000. DOI: 10.1111/jfb.13217.

Douglas, Ron; Hawryshyn, Craig (1990): Behavioral studies of fish vision: An analysis of visual capabilities. In Ron Douglas, Mustafa Djamgoz (Eds.): The Visual System of Fish. Dordrecht: Springer Netherlands, pp. 373–418.

Ebel, Guntram (2014): Modellierung der Schwimmfähigkeit europäischer Fischarten — Zielgrößen für die hydraulische Bemessung von Fischschutzsystemen. In *Wasserwirtschaft* 104 (7-8), pp. 40–47. DOI: 10.1365/s35147-014-1095-1.

Elder, John; Coombs, Sheryl (2015): The influence of turbulence on the sensory basis of rheotaxis. In *Journal of comparative physiology. A, Neuroethology, sensory, neural, and behavioral physiology* 201 (7), pp. 667–680. DOI: 10.1007/s00359-015-1014-7.

Fuentes-Pérez, Juan Francisco; Eckert, Mario; Tuhtan, Jeffrey A.; Ferreira, Maria Teresa; Kruusmaa, Maarja; Branco, Paulo (2018): Spatial preferences of Iberian barbel in a vertical slot fishway under variable hydrodynamic scenarios. In *Ecological Engineering* 125, pp. 131–142. DOI: 10.1016/j.ecoleng.2018.10.014.

Gao, Zhu; Andersson, Helge I.; Dai, Huichao; Jiang, Fengjian; Zhao, Lihao (2016): A new Eulerian–Lagrangian agent method to model fish paths in a vertical slot fishway. In *Ecological Engineering* 88, pp. 217–225. DOI: 10.1016/j.ecoleng.2015.12.038.

Gisen, David C. (2018): Modeling upstream fish migration in small-scale using the Eulerian-Lagrangian-agent method (ELAM). Dissertation. Universität der Bundeswehr München, Munich, Germany. Available online at https://hdl.handle.net/20.500.11970/105158.

Goettel, Michael T.; Atkinson, Joseph F.; Bennett, Sean J. (2015): Behavior of western blacknose dace in a turbulence modified flow field. In *Ecological Engineering* 74, pp. 230–240. DOI: 10.1016/j.ecoleng.2014.10.012.

Goodwin, Richard Andrew; Politano, M.; Garvin, J. W.; Nestler, J. M.; Hay, D.; Anderson, James J. et al. (2014): Fish navigation of large dams emerges from their modulation of flow field experience. In *Proceedings of the National Academy of Sciences* 111 (14), pp. 5277–5282. DOI: 10.1073/pnas.1311874111.

Grimm, Volker; Berger, Uta; Bastiansen, Finn; Eliassen, Sigrunn; Ginot, Vincent; Giske, Jarl et al. (2006): A standard protocol for describing individual-based and agent-based models. In *Ecological Modelling* 198 (1-2), pp. 115–126. DOI: 10.1016/j.ecolmodel.2006.04.023.

Grimm, Volker; Railsback, Steven F. (2005): Individual-based modeling and ecology. Princeton: Princeton University Press (Princeton series in theoretical and computational biology).

Grimm, Volker; Railsback, Steven F. (2012): Pattern-oriented modelling: a 'multi-scope' for predictive systems ecology. In *Philosophical transactions of the Royal Society of London. Series B, Biological sciences* 367 (1586), pp. 298–310. DOI: 10.1098/rstb.2011.0180.

Grimm, Volker; Railsback, Steven F.; Vincenot, Christian E.; Berger, Uta; Gallagher, Cara; DeAngelis, Donald L. et al. (2020): The ODD Protocol for Describing Agent-Based and Other Simulation Models. A Second Update to Improve Clarity, Replication, and Structural Realism. In *JASSS* 23 (2). DOI: 10.18564/jasss.4259.

Jones, M. J.; Baumgartner, L. J.; Zampatti, B. P.; Beyer, K. (2017): Low light inhibits native fish movement through a vertical-slot fishway. Implications for engineering design. In *Fish Manag Ecol* 24 (3), pp. 177–185. DOI: 10.1111/fme.12205.

Kasumyan, A. O. (2011): Tactile reception and behavior of fish. In *J. Ichthyol.* 51 (11), pp. 1035–1103. DOI: 10.1134/S003294521111004X.

Kemp, P. S. (2016): Meta-analyses, Metrics and Motivation: Mixed Messages in the Fish Passage Debate. In *River Res. Applic.* 32 (10), pp. 2116–2124. DOI: 10.1002/rra.3082.

Kemp, Paul S.; Gessel, Michael H.; Sandford, Benjamin P.; Williams, John G. (2006): The behaviour of Pacific salmonid smolts during passage over two experimental weirs under light and dark conditions. In *River Res Applic* 22 (4), pp. 429–440. DOI: 10.1002/rra.913.

Kerr, James R.; Manes, Costantino; Kemp, Paul S. (2016): Assessing hydrodynamic space use of brown trout, Salmo trutta, in a complex flow environment: a return to first principles. In *Journal of Experimental Biology* 219 (Pt 21), pp. 3480–3491. DOI: 10.1242/jeb.134775.

Lacey, J. R. W.; Neary, V. S.; Liao, J. C.; Enders, E. C.; Tritico, H. M. (2012): The IPOS framework: linking fish swimming performance in altered flows from laboratory experiments to rivers. Special Issue Paper. In *River Res. Applic.* 28 (4), pp. 429–443. DOI: 10.1002/rra.1584.

Liao, James C. (2006): The role of the lateral line and vision on body kinematics and hydrodynamic preference of rainbow trout in turbulent flow. In *Journal of Experimental Biology* 209 (20), pp. 4077–4090. DOI: 10.1242/jeb.02487.

Liao, James C. (2007): A review of fish swimming mechanics and behaviour in altered flows. In *Philosophical Transactions of the Royal Society B: Biological Sciences* 362 (1487), pp. 1973–1993. DOI: 10.1098/rstb.2007.2082.

Montgomery, J.; Carton, G.; Voigt, R.; Baker, C.; Diebel, C. (2000): Sensory processing of water currents by fishes. In *Philosophical Transactions of the Royal Society B: Biological Sciences* 355 (1401), pp. 1325–1327. DOI: 10.1098/rstb.2000.0693.

Padgett, Thomas E.; Thomas, Robert E.; Borman, Duncan J.; Mould, David C. (2020): Individual-based model of juvenile eel movement parametrized with computational fluid dynamics-derived flow fields informs improved fish pass design. In *Royal Society open science* 7 (1), p. 191505. DOI: 10.1098/rsos.191505.

Przybilla, Anja; Kunze, Sebastian; Rudert, Alexander; Bleckmann, Horst; Brücker, Christoph (2010): Entraining in trout: a behavioural and hydrodynamic analysis. In *Journal of Experimental Biology* 213 (Pt 17), pp. 2976–2986. DOI: 10.1242/jeb.041632.

Rodríguez, Álvaro; Bermúdez, María; Rabuñal, Juan R.; Puertas, Jerónimo (2015): Fish tracking in vertical slot fishways using computer vision techniques. In *Journal of Hydroinformatics* 17 (2), pp. 275–292. DOI: 10.2166/hydro.2014.034.

Roth, Márcio S.; Jähnel, Christian; Stamm, Jürgen; Schneider, Lisa K. (2021): Turbulent eddy identification of a meander and vertical-slot fishways in numerical models applying the IPOS-framework. In *Journal of Ecohydraulics* 21 (6), pp. 1–20. DOI: 10.1080/24705357.2020.1869916.

Schütz, Cornelia; Henning, Martin; Czerny, Rebekka; Herbst, Marcus; Pitsch, Matthias (2021): Addition of auxiliary discharge into a fishway - A contribution to fish pass design at barrages of large rivers. In *Ecological Engineering* 167. DOI: 10.1016/j.ecoleng.2021.106257.

Silva, Ana T.; Katopodis, Christos; Santos, José M.; Ferreira, Maria Teresa; Pinheiro, António N. (2012): Cyprinid swimming behaviour in response to turbulent flow. In *Ecological Engineering* 44, pp. 314–328. DOI: 10.1016/j.ecoleng.2012.04.015.

Silva, Ana T.; Lucas, Martyn C.; Castro-Santos, Theodore; Katopodis, Christos; Baumgartner, Lee J.; Thiem, Jason D. et al. (2018): The future of fish passage science, engineering, and practice. In *Fish and Fisheries* 19 (2), pp. 340–362. DOI: 10.1111/faf.12258.

Smith, David L.; Goodwin, Richard Andrew; Lai, Young; Nestler, John Michael (2014): Fish path selection and fatigue in complex, continuous velocity fields. In Atle Harby, Håkon Sundt (Eds.): 10th International Symposium on Ecohydraulics. 10th International Symposium on Ecohydraulics. Trondheim.

Tan, Junjun; Tao, Lin; Gao, Zhu; Dai, Huichao; Shi, Xiaotao (2018): Modeling Fish Movement Trajectories in Relation to Hydraulic Response Relationships in an Experimental Fishway. In *Water* 10 (11). DOI: 10.3390/w10111511.

The OpenFOAM Foundation (2014): OpenFOAM. Version 2.3.1. Available online at https://github.com/OpenFOAM/OpenFOAM-2.3.x, checked on 4/28/2020.

Thiele, Jan C.; Kurth, Winfried; Grimm, Volker (2014): Facilitating Parameter Estimation and Sensitivity Analysis of Agent-Based Models. A Cookbook Using NetLogo and 'R'. In *JASSS* 17 (3). DOI: 10.18564/jasss.2503.

Wang, H.; Chanson, H. (2018): Modelling upstream fish passage in standard box culverts. Interplay between turbulence, fish kinematics, and energetics. In *River Res. Applic.* 128 (5), p. 245. DOI: 10.1002/rra.3245.

Webb, Paul W. (1975): Hydrodynamics and Energetics of Fish Propulsion. Edited by J. C. Stevenson. Fisheries Research Board of Canada. Ottawa, Canada (Bulletin of the Fisheries Research Board of Canada, 190). Available online at https://scholarworks.umass.edu/fishpassage_journal_articles/569.

Willis, Jay (2011): Modelling swimming aquatic animals in hydrodynamic models. In *Ecological Modelling* 222 (23-24), pp. 3869–3887.

Windsor, Shane P.; Norris, Stuart E.; Cameron, Stuart M.; Mallinson, Gordon D.; Montgomery, John C. (2010): The flow fields involved in hydrodynamic imaging by blind Mexican cave fish (Astyanax fasciatus). Part II: Gliding parallel to a wall. In *The Journal of experimental biology* 213 (22), pp. 3832–3842. DOI: 10.1242/jeb.040790.

Zielinski, D. P.; Voller, VR.; Sorensen, P. W. (2018): A physiologically inspired agent-based approach to model upstream passage of invasive fish at a lock-and-dam. In *Ecological Modelling* 382, pp. 18–32. DOI: 10.1016/j.ecolmodel.2018.05.004.

8 Supporting information appendix

8.1 S1 Brown trout movement observations, setup 1

Unpublished movement data from Schütz et al. (2021). See S1_brown_trout_setup1.xlsx.

Column name	Definition	Units
fish_id	"1e" is setup 1; "2c" is setup 2. Next part of the ID is trial number, and last is fish number (3 per trial).	-
time_stamp	Time of observation, interpolated from minutes to seconds by event count per minute	UTC DD.MM.YYYY HH:MM:ss
sec	Seconds since start (lifting of staging area mesh screen)	s
swarmsize	Size of group observed, 1-3 fish, "group" was defined subjectively by swimming together	-
x_raw	Longitudinal position observed, axis zero at flume start	m
y_raw	Lateral position, observed to be close to left wall/screen (L), right wall/screen (R), or close to neither wall/screen (M). Used for pattern P1.	Categorical
z_raw	Vertical position observed in the water column, either close to water surface (o), close to bottom (u), or in between (m). Used for pattern P2.	Categorical
x_value	Longitudinal position, computed in postprocessing, axis zero at slot. Used for patterns P3, P4, and P5.	m
y_value	Lateral position, computed in postprocessing, defined from y_raw as being either 0.1 m away from a wall/screen or in the middle. Coordinates account for inclined screen. Used only for computation of cells in Figure 2, not for determining pattern P1.	m

8.2 S2 Brown trout movement observations, setup 2

See S2_brown_trout_setup2.xlsx. Description above.

8.3 S3 Hydraulic field data

See S3_hydraulic_fields.zip containing S3a_hydraulic_fields_setup1.csv and S3b_hydraulic_fields_setup2.csv. For coordinate system origin, see Figure 1.

Column name	Description	Units
ccx, ccy, ccz	Cell center coordinates	m
u, v, w	Velocity vector components	m/s
acclMag	Acceleration magnitude	m/s^2
alpha.water	Water phase, share of cell volume	-
k	Turbulence kinetic energy (TKE)	m^2/s^2

8.4 S4 Behavioral model description

See S4_Gisen_behavioral_model_description.pdf

8.5 S5 Behavioral model code

See https://github.com/baw-de/ELAM-flume. The code is written in C++ and Fortran 90. The Fortran code uses the FORTRAN 77 fixed format for historic reasons.

8.6 S6 Root mean square errors

See S6_RMSE_5_stimuli_2_setups.csv. The order is Wall distance (setup 1, setup 2) – Acceleration 1, 2 – Velocity 1, 2 – TKE 1, 2 – Baseline 1, 2.

Supporting Information 1

Click here to access/download
Supporting Information
S1_Brown_trout_setup1.xlsx

Supporting Information 2

Click here to access/download
Supporting Information
S2_Brown_trout_setup2.xlsx

Click here to access/download
Supporting Information
S6_RMSE_5_stimuli_2_setups.csv

Click here to access/download
Supporting Information
S4_Behavioral_model_description.pdf

Click here to access/download
Supporting Information - Compressed/ZIP File Archive
S3_hydraulic_fields.zip

Development of behavioral rules for upstream orientation of fish in confined space ~~employing wall distance and hydraulic stimuli~~

David C. Gisen[1*], Cornelia Schütz[2], Roman B. Weichert[1]

[1] Waterways and environment unit, Bundesanstalt für Wasserbau, Karlsruhe, Germany

[2] Animal ecology unit, Bundesanstalt für Gewässerkunde, Koblenz, Germany

* Corresponding author
E-mail: david.gisen@baw.de (D.C.G.)

Abstract

Improving the effectiveness of fishways requires a better understanding of fish behavior near hydraulic structures, especially of upstream orientation. One of the most promising approaches to this problem is the use of model behavioral rules.

We developed a three-dimensional individual-based model based on observed brown trout (*Salmo trutta fario*) movement in a laboratory flume and~~. We~~ tested it against two hydraulically different flume setups~~ with different discharges~~. We used the model to examine which of five behavioral rule versions would best explain upstream trout orientation. The v~~V~~ersions differed in the stimulus~~ response mechanism~~ for swim angle selection. The baseline stimulus was positive rheotaxis with a random component. It was supplemented by attraction towards either lower velocity magnitude, constant turbulence kinetic energy, increased flow acceleration, or smaller wall distance.

As expected, modeling rheotaxis was central to matching the laboratory observations. ~~In combination with a random component for swim angle selection, t~~The baseline ~~model ~~version ~~version ~~was already able to explain large parts of the observed behavior. Mixed results for velocity magnitude, turbulence kinetic energy, and flow acceleration ~~supported the hypothesis~~indicated that the brown~~ live~~ trout did not orient primarily by means of flow features~~ characterized by these variables~~, probably because the flow field did not challenge them physically.

The wall distance version produced ~~S~~significantly improved results, suggesting~~ed~~ that wall distance was the dominant orientation stimulus for brown trout in our hydraulic conditions. The absolute root mean square error (RMSE) was small for the best parameter set (RMSE = 9 for setup 1, RMSE = 6 for setup 2). Our best explanation for this result is dominance of the visual sense favored by absence of challenging hydraulic stimuli. We conclude that~~,~~ under similar conditions (moderate flow and visible walls), wall distance could be a relevant stimulus in confined space, particularly for fishway studies and design in IBMs, laboratory, and the field.~~ We show that wall distance was most likely perceived visually. For dark or turbid water, also acoustic or tactile reception of walls would be conceivable. We conclude that wall distance estimation should be considered more frequently as an orientation stimulus in confined space. This is true wherever conditions enable wall distance perception, either in individual-based models, laboratory flumes, or in the field.~~

1 Introduction

Worldwide, fishways play a key role in efforts to restore upstream-directed fish migration at dams. The ~~W~~widely differing~~varying~~ effectiveness of existing projects (Bunt et al. 2012; Bunt et al. 2016) illustrates ~~how~~ that ~~challenging~~ their design is still challenging, especially for species other than Pacific salmon (Kemp 2016). Improving fishway effectiveness for all species requires a better understanding of fish behavior near hydraulic structures, especially of orientation and navigation ~~Orientation can be defined as directional response to local conditions and navigation as movement towards a goal outside the local sensory environment~~ (Willis 2011; Silva et al. 2018). One of the most promising approaches to better understand ~~fish~~ such behavior and “a high research priority” (Silva et al. 2018) is the ~~use~~ development of behavioral rules. ~~(Silva et al. 2018).~~ Behavioral rules are the logical and/or algebraical formulation of behavioral hypotheses. They ~~are~~ can be implemented and tested in individual-based models (IBMs, Grimm and Railsback 2005). ~~Development of behavioral hypotheses for orientation and testing derived rules requires data on movement and the selection of stimuli.~~

IBMs allow to implement very closely the concepts of orientation (directional response to local conditions, Willis 2011) and navigation (movement towards a goal outside the local sensory environment, Willis 2011). Typically, orientation is ~~usually~~ the direct outcome of a behavioral rule using a local stimulus, and navigation ~~typically~~ emerges indirectly from the sum of orientation decisions over time (e.g., Goodwin et al. 2014; Smith et al. 2014; Arenas et al. 2015; Zielinski et al. 2018). Together with their ability to integrate CFD (computational fluid dynamics) model results, IBMs are therefore highly suitable to support fishway design.

~~The~~A ~~External factors are typically the~~ ~~A~~ preferred objective of ~~the model~~ research for fishway design are hydraulic stimuli, because they can be~~ir~~ manipulat~~ion~~ed ~~may be used in fishway design~~ to optimize passage ~~efficency~~ efficiency. The most widely used stimulus in fish orientation IBMs, regardless of whether the model considers ~~no matter if for the~~ upstream or downstream fish migration~~direction~~, is

~~The most common hydraulic stimuli are velocity direction, velocity magnitude, and one of the various turbulence variables, as described in the following studies on stimuli response. Their wide differences in features like confinement (tailrace to fishway pool), method (laboratory observation or IBM), and migration direction (upstream or downstream) does only affect the particular response hypothesis, but not stimuli selection in general.~~

~~Velocity is defined by direction and magnitude. V~~ velocity direction ~~is used on nearly all reviewed fish migration models~~ (Goodwin et al. 2014; Gao et al. 2016; Tan et al. 2018; Zielinski et al. 2018; Padgett et al. 2020)~~(Zitate)~~. It is a fundamental stimulus, as it informs positive and negative ~~in most studies, as~~ rheotaxis, which are ~~is~~ crucial for guiding ~~both~~ upstream and downstream navigation, respectively ~~on different spatial scales~~ (Arnold 1974; Elder and Coombs 2015; Coombs et al. 2020) ~~and has shown significance as model stimulus in small scale models (Zitate)~~. Velocity magnitude is also commonly considered~~(Zitate)~~ (Arenas et al. 2015; Goodwin et al. 2014; Gao et al. 2016; Tan et al. 2018; Zielinski et al. 2018; Padgett et al. 2020) and especially important for upstream ~~navig~~migration, ~~most prominently for~~

Commented [SCUB1]: Line 48 – While the introduction touches briefly upon aspects of the fuller study, it lacks sufficient detail to understand why certain selections of behaviors and guiding stimuli were chosen. In this instance, citing the IPOS framework to describe what aspects of turbulence are important to fish behaviors would be relevant.

ascent (e.g., Padgett et al. 2020). Since ascentAssince the energy demand for swimming against the flow is proportional to the cube of relative fish velocity (Wang and Chanson 2018)this requires energy,.

fish have developed strategies to reduce their effort (Kerr et al. 2016)e.g. . One strategy is to avoiding zones of higher velocity for moving and holding (low-velocity seeking) and c, as energy demand is proportional to the cube of relative fish velocity (Wang and Chanson 2018). Corresponding behavior was observed in laboratory flumes for longnose dace (Dockery et al. 2017) and brown trout (Kerr et al. 2016). It was successfully modeled in an horizontally unconfined IBM for carp and sturgeon (Zielinski et al. 2018). Apart from velocity direction and magnitude, stimulus selection to formulate concrete behavioral rules remains difficult (Goodwin et al. 2014). To integrate information on turbulence into the behavioral rulesIBMs, the turbulence kinetic energy (TKE) is a common choice in IBMs (Gao et al. 2016; Tan et al. 2018). TurbulenceIt may also beOne study integrated it as velocity fluctuation (Zielinski et al. 2018). Other studies try to Other approaches to capture additional hydraulic information in IBMs include using advective (spatial) acceleration (Goodwin et al. 2014; Smith et al. 2014; Arenas et al. 2015) or theas strain rate (Tan et al. 2018).

Fish studies from laboratory flumes can offer .

Laboratory flume studies offer more comprehensive data on fish upstream orientation than integrated in the existing IBMs. Also, they add further potential stimuli for modelling upstream orientation, for example the horizontal Reynolds shear stress (τ_{xy}), turbulence intensity, and eddy orientation and scale (see Lacey et al. 2012 for a framework). However, results are not consistentmanifold and could depend on the specific setup and/or species tested. In different studies, dace preferred adapted turbulence levels (TKE and τ_{xy}) (Goettel et al. 2015), adult Iberian barbels primarily avoided areas of high τ_{xy} (Silva et al. 2012), and brown trout preferred low drag areas (Kerr et al. 2016). Eddy orientation and scale did influence Iberian barbel behavior (Silva et al. 2012), but are still difficult to define in common vertical-slot fishways (Roth et al. 2021) and are not used in IBMs to date. As for IBMs, the correct choiceselection of hydraulic variablesstimuli for laboratory studies is aremains controversial topic (Kerr et al. 2016). Turbulence as a model stimulus is usually implemented usually characterized asby means of turbulence kinetic energy (TKE) and/or Reynolds shear stress (τ_{xy}), which represent different components of the specific Reynolds stress tensor.Avoiding areas of low and high TKE proved a succesful model rule for the simulation of upstream swim paths through a vertical slot fishpass. In laboratory studies, adult Iberian barbels avoided areas of high τ_{xy} and were disoriented by eddies of size similar to their body length (BL = 0.15–0.35 m) (Silva et al. 2012). Analysis of the turbulence levels (TKE and τ_{xy}) at chosen locations showed a clear preference for maintaining adapted turbulence levels in dace (Goettel et al. 2015). Brown trout preferred areas of low drag, which was a better explanation than TKE, turbulence intensity, and τ_{xy} in one study (Kerr et al. 2016). In an IBM, two trout paths were best reproduced by avoiding areas of low and high TKE (Gao et al. 2016). In a follow-up model, TKE, velocity magnitude, and strain rate were combined, with the former two having larger influence than the latter on the tracks of three silver carp (Tan et al. 2018).

Aadvective acceleration was applied several times as an IBM stimulus for guiding juvenile salmon descent (Goodwin et al. 2014; Arenas et al. 2015). It was validated by observations from seven large dams

using an Eulerian-Lagrangian-agent method (ELAM, Goodwin et al. 2014). For acceleration use in ascent, a first unvalidated model of steelhead behavior exists (Smith et al. 2014).

Non-hydraulic external stimuli for fish orientation, such as visual, acoustic, olfactory, acoustic, thermal, and magnetic stimuli, are rarely investigated in flows that are comparable to a fishway flow and the authors are not aware of publishedcorresponding fish migrationorientation IBMs that include such factorsstimuli. This is particularly notable for vision, as it is considered important for orientation (Montgomery et al. 2000; Liao 2007). Published laboratory and field studies on upstream orientation While it may not be a significant model stimulus for navigation in large turbid rivers, it should be considered within the confinements of fishways. Vision was controlled only in few studies on space use of fish moving against the flow (Liao 2006; Kerr et al. 2016). Studies with similar objectives using visual stimuliion as explanatory variable were are limited to general phototaxis, i.e. attraction or repulsion (Elder and Coombs 2015; Jones et al. 2017). IBMs offer new opportunities to investigate vision in greater detail. For example, it is known that visually determined angle and distance can be used by goldfish to estimate object sizes (Douglas and Hawryshyn 1990).

For the migratory behavior and successful upstream migration, a basic

Especially motivation to swim against the flow is crucial. However, this motivation but may vary widely from diadromous to potamodromous species and even within these categoriesbetween species and even between individuals (Kemp 2016). EStill, existing upstream Oorientation IBMs therefore focus on a specific species during their obligatory up- oder downstream migration, avoid to model motivation as an internal state by determining that fish as aconstantly try to preference to move upstream presupposition of the model(Smith et al. 2014; Gao et al. 2016; Tan et al. 2018; Zielinski et al. 2018; Padgett et al. 2020). However, differing iInternal stimulistates are relevant for orientation and navigation and mayare necessary to produce differing behavioral answers toto identical environmental situations (Lennox 2019). For example, Tthis definitionapproach prevents prohibits e.g. emergence of the

Another potentially important aspect not yet addressed in upstream orientation IBMs is the ability to drift, recover, and repeat passage attempts after initial failure (Castro-Santos 2004).

Furthermore, existing IBMs for upstream migration (Smith et al. 2014; Gao et al. 2016; Tan et al. 2018; Zielinski et al. 2018; Padgett et al. 2020) still lacksuffer from a lack of comparablecomprehensive quantitative testing as available for IBMs for downstream migration. (Most orientation IBMs focus on a specific species during their obligatory up- oder downstream migration, allowing to integrate respective motivation to migrate as a presupposition of the model.)

IBMs which are tested against large data sets of real observed fish are only avaliable for orientation of salmon smolts during downstream migration (Goodwin et al. 2014; Arenas et al. 2015).,

In the present study, we thus developed a new IBM for fish upstream orientation and navigation in confined space to support fishway design. while published IBMs for upstream migration (Smith et al. 2014; Gao et al. 2016; Tan et al. 2018; Zielinski et al. 2018; Padgett et al. 2020) still lack comparable quantitative testing.

Developing an IBM for an existing laboratory study on fishways appears to be a promising way to Our long-term purpose is to findformulate and test behavioral rules for fish tothat enable predictions of fishway attraction and passage efficiency and thus can support fishway design. In the present study, we developed such a new IBM for upstream orientation and navigation. For the first time, such a model was tested against detailed data of fish (brown trout) movement in a laboratory flume, and allowed holding behavior, recovery, and thus repeated passage attempts. we approached it by asking how well certain external stimuli explain orientation and navigation on a fishway scale. gibt es hier doch Beispiele in IBMs?

The following Ffive external stimuli were selected for testing based on the model survey aboveBased on the literature and theoretical considerations, we selected the following five stimuli for testing:

- positive rheotaxis as a fundamental behavior in upstream migrating fish,
- velocity magnitude as a fundamentalvery likely cause forbeing fundamentally related to energy consumptiondemand,
- TKE as the one of the most common and general turbulence variables, containing turbulence intensity,
- flowspatial acceleration as having been tested against the largest data sets by far (for downstream migration) and containing the strain rate,
- wall distance as a potential proxy for to the little investigated, but potentially important visual orientation.

To address our question, we developed and applied a new IBM for upstream orientation and navigation of fish. For the first time, such a model (a) was tested against detailed data of fish movement in a laboratory flume, and (b) allowed holding behavior, recovery, and thus repeated attempts.

To find possible behavioral rules for upstream fish orientation and navigation in confined space, e.g. in fishways, To find possible behavioral rules for upstream fish orientation and navigation in confined space, e.g. in fishways,

For their implementation as concrete behavioral rules, we developed a new IBM based, on brown trout movement in a laboratory flume. Being ELAM-type (Goodwin et al. 2014), the IBMit consists of a (*Eulerian*) computational fluid dynamics (CFD) model, a software framework (*Lagrangian* transport etc.), and thea new behavioral model (defining *agent* behavior), and the associated software framework.

In the work described in this paperThe present paper describes how, we (1) defined movement patterns from brownthe brown trout flume data, (2) developed and tested a CFD model to generate stimuli data, (3) developed and tested a behavioral model to reproduce the trout movement patterns, and

Commented [SCUB2]: . I'm a bit concerned that it is unclear how much of this work is Goodwin's and how much is new work.

Commented [SCUB3]: The introduction would further benefit from more details on how the current effort extends or differs from previous IBMs. Specifically, it is not clear how the model is differentiated from the ELAM used by Goodwin et al. (2014). The only reference to the ELAM in on Lines 73 and 84 stating the proposed model is "ELAM-type". I feel the general reader is not going to understand what this means.

(4) examined which of the five external stimuli ~~(baseline rheotaxis, velocity magnitude, TKE, flow acceleration, and wall distance)~~ would best explain upstream brown trout orientation and navigation in confined space.

2 Methods

2.1 Flume setups

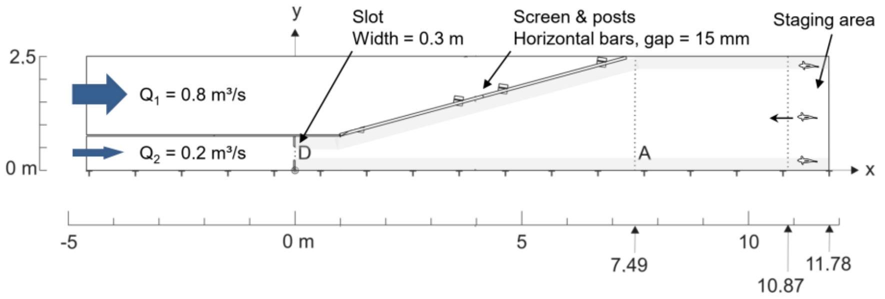

Figure 1: Plan view of flume setup 1. Total length and width = 16.78 m x 2.50 m, water depth = 0.60 m. Flow direction was from left to right. The slot was removed in setup 2 to alter the flow field. Transparent gray areas mark left and right zones (in flow direction) for pattern P1 (defined below, section 2.2). *A* and *D* mark control lines to filter inactive fish and to define pattern P5.

For developing and testing the behavioral model, we used unpublished brown trout movement data from a previous, unrelated study (wild *Salmo trutta fario,* n = 66, mean body length ± standard deviation (SD) = 0.27±0.04 m, Schütz et al. 2021) ~~(Schütz et al. 2021)~~. Fish were caught in close-by rivers during their spawning period in October/November, when an increased motivation to migrate can be expected (Schütz et al. 2021). The study was conducted in a wide indoor flume (Figure 1). The external walls and bottom consisted of smooth acryl glass and metal and the internal walls consisted of coated wood. Water temperature was 16.2-18.2 °C. Water was clear and evenly illuminated. All care and procedures involving handling and holding fish were conducted as stated and permitted by the district government Karlsruhe (license AZ 35-9185.82/A-6/16).

W~~The original fish experiment investigated the influx of auxiliary discharge into a fishway and its effects on fish passage. Two Fish were observed in two hydraulically different setups~~e use two hydraulically different setups from Schütz et al. (2021) to test the application range of our model~~were compared (Schütz et al. 2021)~~.~~.~~ The first setup included a jet created by a slot; the second setup had no slot and consequently, no jet. ~~-~~The total discharge was Q = 1.00 m^3/s in both setups to produce a~~-~~ jet velocity of U = 1.5 m/s, which is typical for slots of large multi-species fishways in Germany. The flume was wide enough to enable distinct lateral movement, but narrow enough to influence behavior through the walls and screen.

Commented [SCUB4]: The authors need to more explicitly state what hypotheses they are testing and why. The preceding paragraphs lists evidence that points to a lot of hydraulic variables that could be influential to fish movement. The authors do not explain why they examine baseline rheotaxis, velocity magnitude, TKE, flow acceleration, and wall distance relative to other choices including turbulent intensity, velocity gradients, eddy sizes, etc.

Commented [SCUB5]: . I love this stuff and I think its interesting work, I just want more information in the methods that is clearly organized and suggest a full rewrite where you focus on streamlining terminology, writing good paragraphs, and correcting grammar errors.

Commented [SCUB6]: Figure 1. The figure caption is the first mention of patterns P1 and P5 without defining them. I suggest moving this statement into the main text after P1 and P5 are defined.

Commented [SCUB7]: I don't understand why the fish swimming study was designed the way it was. Was it specifically designed to calibrate/validate the model?

Commented [SCUB8]: How were the set-ups chosen for the laboratory tests and how does this relate to the central hypotheses being tested?

Commented [SCUB9]: How were the set-ups chosen for the laboratory tests and how does this relate to the central hypotheses being tested?

Fish were released unmarked in batches of three at the downstream end (staging area in Figure 1) and were . Fish were not re-used only once to avoid learning effects. Fish positions were manually recorded in real time independently by two biologists upon notable change. A change of position was defined as followseither:

- crossing a meter line of the flume in longitudinal direction
- moving between three positionszones in lateral direction: close to left wall, close to right wall, far from wall (close meaningings max. ~ 0.25 cm)
- moving between three positionszones verticallyin vertical direction (close to bottom, close to surface, in between (close meaningings max. ~ 0.15 cm)

between x = 0.00 m and x = 9.74 m and visits to the staging area were tracked recorded manually and independently by two biologists upon notable change. The estimated spatial accuracy was in the order of sub-meters in longitudinal direction (side marks) and few decimeters in the vertical direction. In the lateral direction, position was recorded as being either "close" (< a few decimeters) or "far" from botha walls/the or the screen. Moreover, it was recorded if fish swam alone or in groups of two or three. Records were attributed to the current test minute. During postprocessing, longitudinal records were assigned the precise position of their side mark. multiple position changes Temporal records within the same minute were distributed evenly over the seconds were converted to seconds and evenly distributed within this test minute (appendices S1 and S2). Data were verified qualitatively using video records.

To ensure that all fish were actually motivated to migratemove during the experiment, only those fish that swam at least overcrossed line *A* *A* (Figure 1) within 30 minutes after removing the staging mesh were considered valid Fish which did not cross line *A* within 30 min after removing the staging mesh screen were excluded as inactive. A trial was finished either after all three fish passed line *D* or 60 min after the first crossing of line *A*. Data points following a passage of line *D* were also excluded. After filtering, we obtained n = 25 tracks for setup 1 and n = 24 tracks for setup 2.

2.2 Movement patterns

We processed the trout track data using *patterns* in the sense of pattern-oriented modeling (Grimm and Railsback 2012) using MATLAB R2018b. We defined five patterns, P1-P5, which to capture the most striking spatial behaviors observed during the original experiment. They included a preference for wall and bottom proximity as well as and frequently swimming back and forth (turns) in the whole domain:

P1, lateral distribution: The flume was divided into a left, middle, and right zone (looking downstream, Figure 1). The dividing lines were located at a lateral distance of Δy = 0.25 m (10 % flume width) to one of either side walls or the screen. Time spent by single fish (i.e., center of mass) was summed for each zone. To account for the differences in total track duration (see Rodríguez et al. 2015), resulting time sums per fish and zone, $t_{zone,i}$, were divided by the particular fish's track duration, $t_{s,i}$, to get relative track fractions, s_{zone}. Finally, s_{zone} was averaged by the fish count, n, to get:

$$\bar{s}_{zone} = \frac{1}{n}\sum_{i=1}^{n}\frac{t_{zone,i}}{t_{s,i}} * 100 \ \ [\%] \qquad \text{(Eq. 1)}$$

Commented [SCUB10]: 8. Line 109-119 – The methods and scales at which fish movement was tracked is unclear. Where observers tracking movements in real-time on paper as well as noting location relative to the wall or screen or position in a group? How often was the position recorded? The authors state that observations were verified qualitatively with video records, why not use the video to obtain more detailed tracks. Overall, the methods on how tracking was accomplished needs significant more detail.

Commented [SCUB11]: . Why would learning effects matter in this scenario?

Commented [SCUB12]: Line 110 – Where does the "x=9.74" come from? Figure 1 indicates an observation point at x=7.49.

Commented [SCUB13]: Line 121-147 – How were these patterns chosen and what hypotheses drove these decisions? Their selection seems somewhat random as written. For example, what details informed splitting the channel at a distance of 0.25 m? Does this distance coincide with an observed hydraulic feature or behavior?

where i was a fish iterator. The resulting three lateral zone averages constituted pattern P1. As it ~~constitutes~~ is the most direct ~~representation~~ outcome of upstream orientation ~~effects~~, matching it was a major goal of model development.

P2, vertical distribution: Vertically, the flume was divided into a surface, middle, and bottom zone. The dividing lines were located at a vertical distance of Δz = 0.15 m (25 % water depth) to either the water surface or flume bottom, respectively. Tracks were averaged ~~like for P1~~using Eq. 1 to obtain three vertical zone average values. Due to the shallow water, we ~~assumed~~ expected P2 to be of less significance for orientation.

P3, turn ~~area~~ zone and *P4, few turns*: Turns were defined as changes in the longitudinal movement direction, x, leading to a displacement $\Delta x \geq 2$ BL before the next direction change. About half of the fish in both data sets performed 4 or less turns. To avoid bias, the data set was divided into turning (> 4 turns, P3, n_{turns}) and few-/no-turning fish (≤ 4 turns, P4, $n_{few\text{-}turns}$). For computing P3, the flume was divided longitudinally into three zones of equal length, in which the relative turn shares were computed and averaged by n_{turns} analog to Eq. 1. P4 was defined as $n_{few\text{-}turns}/n$.

P5, arrival rate: The count of fish navigating the total length of the experimental flume area and crossing line D, divided by the count of fish crossing line A, formed P5: $n_D/n \cdot 100$ [%].

For use in discussion, we also calculated the lateral distribution of fish at the start position and the proportion of track time fish spent without other fish nearby (i.e., not shoaling).

2.3 Computational fluid dynamics model

A CFD model was employed to calculate the velocity, TKE, and acceleration fields of both setups. We applied the free-surface solver *interFoam* of OpenFOAM 2.3.1 (The OpenFOAM Foundation 2014) to solve the 3D incompressible URANS (unsteady Reynolds-averaged Navier-Stokes) equations.

A hexahedron-dominant unstructured mesh was generated using *snappyHexMesh*. Base resolution was uniform Δ_{xyz} = 5 cm, with local refinement to Δ_{xyz} = 1.25 cm around the slot and screen posts (Figure 1). Horizontal screen bars were omitted, as their influence on the velocity and turbulence field was negligible with respect to behavioral data accuracy (Gisen 2018). Total cell count for setup 1 was 848,694.

Inlet flow rates and outlet fixed water level were set to the laboratory values. For turbulence closure, we chose the k-ω-SST model. At the inlet, TKE (≙ k) was set to k = 0.001 m²/s² and the specific rate of TKE dissipation was set to ω = 1 Hz. No-slip conditions (velocity vector U = (0,0,0)) and a small sand-equivalent roughness coefficient ($k_S = 1 \cdot 10^{-5}$ m) were set at the walls and bottom.

The fields of velocity U and water/air distribution α_{water} were initialized with a largely converged solution and ra~~u~~n for 80 s of simulated time. U was averaged over the final 20 s to obtain the steady flow velocity vector field $\vec{U}_m$. $\vec{U}_m$ can be expressed either as three-component vector (u,v,w) or as magnitude U_m along with its horizontal and vertical angles γ_m and β_m.

Commented [SCUB14]: I would like more explanation for why decisions were made ....e.g. why use averaged/steady results from the CFD model?

Commented [SCUB15]: Please provide more information on CFD model setup, calibration and validation.

Commented [SCUB16]: Line 155 – Omission of the screen bars is not sufficiently detailed in the methods. I understand the reason for not modelling the bars explicitly, but why not model them as a permeable surface to replicate some of the fine-scale turbulence. See Ho et al. (2011).
Ho, J., Coonrod, J., Hanna, L.J., and Mefford, B.W. 2011. Hydrodynamic modelling study of a fish exclusion system for a river diversion. River Res. Applic. 27: 184–192. doi:10.1002/rra.1349.

TKE is defined as half the sum of the velocity components' variances (squared turbulence intensities):

$$TKE = \frac{1}{2}\overline{U_i'U_i'} = \frac{1}{2}\left(\overline{u'^2} + \overline{v'^2} + \overline{w'^2}\right) \quad \left[\frac{m^2}{s^2} = \frac{J}{kg}\right] \qquad \text{(Eq. 2)}$$

using Reynolds decomposition, $U_i = \overline{U_i} + U_i'$.

~~Advective~~ Spatial acceleration a is caused by changes in the flume cross-section and by friction, in contrast to temporal acceleration caused by transient processes. ~~It is computed from U_m, i.e. temporal acceleration is absent. In 3D Cartesian coordinates,~~ ~~Aadvective~~Spatial acceleration is the product of velocity and velocity gradient (which comprises strain rate and rotation rate). In 3D Cartesian coordinates, it reads

$$a = \begin{pmatrix} a_x \\ a_y \\ a_z \end{pmatrix} = (U_m^T \, grad \, U_m)^T = \left((u \quad v \quad w) \begin{pmatrix} \frac{\partial u}{\partial x} & \frac{\partial v}{\partial x} & \frac{\partial w}{\partial x} \\ \frac{\partial u}{\partial y} & \frac{\partial v}{\partial y} & \frac{\partial w}{\partial y} \\ \frac{\partial u}{\partial z} & \frac{\partial v}{\partial z} & \frac{\partial w}{\partial z} \end{pmatrix} \right)^T \quad \left[\frac{m}{s^2}\right] \qquad \text{(Eq. 3)}$$

where the superscript ^{T} denotes transposition from column to row vector. Its magnitude $|a|$, U_m, and TKE were used as hydraulic stimuli variables in the behavioral model.

Commented [SCUB17]: Line 172 – Again, what underlying hypothesis drove the selection of just these 3 hydraulic variables?

2.4 Behavioral model description

A complete, detailed model description, following the ODD (overview, design concepts, details) protocol (Grimm et al. 2006; Grimm et al. 2020) can be found in appendix S4. Here, we provide a ~~summary.~~

~~The~~summary. The overall purpose of our model was to find ~~possible stimulus-response relations (~~behavioral rules~~)~~ for fish to e.g. enable predictions of fishway attraction and passage efficiency in the long-term. Specifically, we addressed the following question: How well do different external stimuli explain orientation and navigation of upstream moving brown trout? ~~Orientation was modeled directly as stimulus-guided swim angle selection, and navigation was expected to emerge indirectly as a result of the whole behavioral model.~~

The entities of the model were fish and mesh cells. Fish were represented as spatially explicit points in 3D space. Their key state variables were position, motivation, and fatigue. Fish movement was modeled kinematically, i.e. there was no influence of fish on the flow field. Key state variables of the mesh cells were the cell label number and the hydraulic variables $|a|$, U_m, TKE, and α_{water}. Spatially, the behavioral model covered the fish-accessible areas of the CFD model (i.e. without the air phase and without areas upstream of the screen). The spatial resolution was that of the CFD model, i.e. 5 cm or smaller. The temporal extent was 60 min of clock time, equal to the maximum laboratory trial duration, divided into constant time steps of Δt = 0.5 s.

Each model run used n_{model} = 100 fish of BL = 0.27 m (as measured), each being assigned to either a "fast" or "slow" category. In this way we tried to model individual traits which could influence migratory

tendencies, such as being bold or shy (Chapman et al. 2011) or being physically strong or weak. Model fish were positioned only in the left and right zones of the staging area (Figure 1) as per observations. Their ~~initial~~ exact distribution was ~~steered~~ controlled by a parameter.

~~The following process was repeated e~~Every time step, the model computed~~for every fish to determine~~ a new position for every fish. ~~First~~First, a sensory ovoid consisting of seven points around the fish center was determined (Goodwin et al. 2014). ~~Hydraulic variables were interpolated to these points.~~ They were ~~chosen~~ placed approximately at the skin/water interface, where ~~e.g. pressure fluctuations are mediated by means of~~water motions are detected by the lateral line system (Bleckmann and Zelick 2009). Hydraulic variables were interpolated to these points. Wall distances were computed towards the front and sides.

Commented [SCUB18]: 13. Line 200 – Was distance to the floor included in the wall distance evaluations?

Next, we aimed to balance driving and retaining traits for upstream and downstream movement, summarized by the variables motivation (to swim upstream), *M*, and fatigue, *F* (both range 0.0–1.0). They resembled the typical internal states "need" and "cost" (Willis 2011). We chose this basic representation of internal stimuli because our focus was on orientation and external stimuli. Still, ~~the observation of frequent turns and repeated attempts needed to be initialised byit allowing~~it enabled~~was sufficient for~~ model fish to drift, recover, and swim upstream again~~repeat attempts at critical passages (e.g. the slot)~~, a behavior which we consider crucial for modeling fish migration through fishways (Castro-Santos 2004). Its success was measured foremost by patterns P3 and P4.

~~Next, *motivation (to swim upstream)*~~*M* was increased proportionally to time as long as the model fish ~~was not moving.~~ made no spatial progress, presuming a principle motivation to move upstream. The maximum increase rate was reached after $k_{M,f}$ = 20 s for "fast" fish and $k_{M,s}$ = 130 s for "slow" fish (default values, varied later). ~~*Fatigue*~~ *F* was ~~increased~~computed proportionally to local flow velocity and swim speed. It reached the~~s~~ maximum value ~~was reached~~ at a burst speed of k_F = 25 BL/s (Castro-Santos et al. 2013). We note that more realistic speed-fatigue relations are available (Castro-Santos et al. 2013; Ebel 2014), but they were not required for our purpose of modeling orientation. From the balance of time-averaged ~~*motivation*~~ *M* and ~~*fatigue*~~*F*, one of three horizontal behavioral rules was selected (Figure 2):

- migrating (moving upstream),
- holding (maintaining position),
- drifting (moving downstream while allowing random retardation and/or inclination towards the flow).

~~This approach allowed model fish to drift, rest, and repeat attempts at critical passages (e.g. slots), a behavior which we consider crucial for modeling fish migration through fishways. Its success will be measured foremost by patterns P3 and P4.~~

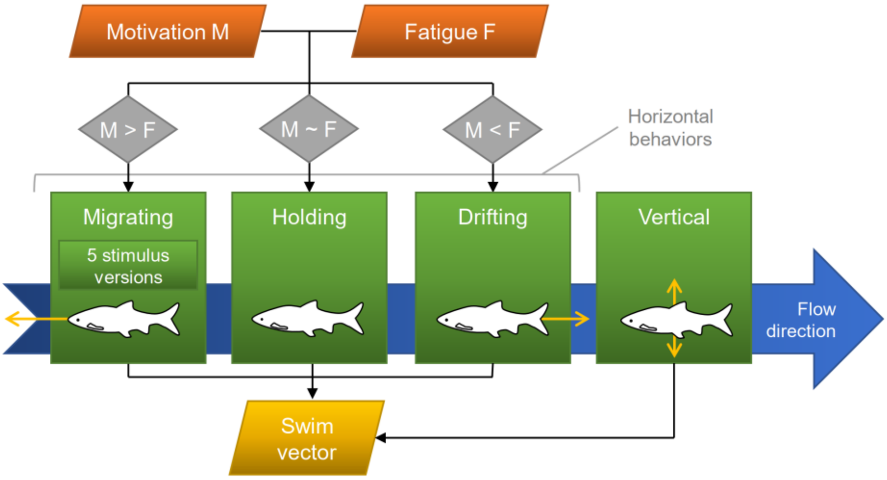

Figure 2: The process of computing the swim vector: Based on the balance between motivation and fatigue, a horizontal behavioral rule is selected. *Migrating* is the key behavioral rule for which five versions are contrasted. *Drifting* behavior includes swimming against, but slower than the flow. In combination with the vertical behavioral rule, the final swim vector is determined.

Migrating was the key behavioral rule. In its baseline version it only depended on the ~~flow~~ velocity direction. It sets the horizontal and vertical swim angles against the ~~flow~~ velocity direction, as we judged rheotaxis to be a fundamental behavior in fish that is always active (e.g. in Mexican tetra, Elder and Coombs 2015). To also capture smaller, unpredictable behavior variations, a limited random angle was added to or subtracted from the horizontal swim angle. Migrating was expected to have the most direct effect on pattern P1.

Our guiding question ("how well do external stimuli explain upstream orientation?") was addressed by adding four ~~options~~ different stimuli for orientation to the baseline migrating behavioral rule. ~~Each was based on a different stimulus for orientation.~~ That is, the horizontal swim angle could be adjusted in either one of five ~~different~~ alternative stimulus versions:

- Baseline (rheotaxis): Against ~~flow~~ velocity direction & towards random side by random magnitude as described above.~~;~~
- Velocity: Baseline & towards side of lower velocity magnitude ~~(low-velocity seeking, e.g. Zielinski et al. 2018);~~.
  Rationale: ~~To a~~Avoiding zones of high~~er~~ velocity to save energy (low-velocity seeking) is a common strategy for fish and was observed e.g. in laboratory flumes for longnose dace (Dockery et al. 2017) and brown trout (Kerr et al. 2016) and modeled in an IBM for carp and sturgeon (Zielinski et al. 2018).
  ~~reason: velocity magnitude stands for the extent to which fish have to consume energy for upstream movement which may be an important factor for fish decisions (Zitat)~~
-

- TKE: Baseline & towards front or side with the smallest TKE difference towards the fish center (constant turbulence level) (constant turbulence level, e.g. Gao et al. 2016; Goettel et al. 2015) ;
  Rationale:
  reason: whileAs it is still unclear how turbulence affects orientation in a specific situation or for a single species and fish size, we chose one of the most common and general 3D turbulence variables, containing the turbulence intensity. Orientation along a constant, adapted turbulence level was observed before in dace (Goettel et al. 2015) and modeled in an IBM (Gao et al. 2016). (constant turbulence level, e.g. Gao et al. 2016; Goettel et al. 2015)
- Advective Spatial acceleration: Baseline & towards side of higher $|a|$ magnitude.;
  Rationale:
  reason: it has been tested and proved significantAcceleration is the hydraulic stimulus tested against the largest data sets by far, although (for downstream migration (Goodwin et al. 2014; Arenas et al. 2015). As it also) and also contains the strain rate (Tan et al. 2018) within the velocity gradient tensor, it is an information-rich representation of the flow field and we consider it potentially useful also for upstream migration (e.g., Smith et al. 2014). We chose attraction towards higher acceleration to mimic our laboratory results.
- Wall distance (as a general proxy for non-hydraulic stimuli): Baseline & towards side of smaller wall distance.
  Rationale:
  reason: wWithin the constrictedconfined space of our model, wall distance it can serve as a potential proxy for the little investigated, but potentially important (Montgomery et al. 2000; Liao 2007) visual orientation. Again, we chose attraction towards smaller wall distance to mimic our laboratory results.

An independent vertical behavioral rule was always active. It limited vertical movement using an elevation difference (Goodwin et al. 2014) and was primarily gauged by pattern P2.

The output of the process described was a 3D swim vector for each fish and time step. The new position was determined from this swim vector, the flow vector at the fish's position, and the time step width. Overall navigational success was measured by pattern P5.

2.4.1 Code and speed

Commented [SCUB19]: Since the behavioral rules and selection of stimuli are the main contributions of this work, I found the model description provided in the main text to be underwhelming. I generally understand the adherence to the ODD protocol, but this should not sacrifice the completeness of the main text to act as a standalone document.

The code of the behavior model and its software framework can be found in appendix S5. The software framework was based on the Fortran 90 code for downstream migrating smolts used in Goodwin et al. (2014). It accounted for e.g. variable storage, sensory point creation, vector transformation from/to Cartesian coordinates, and pseudo-random number creation. We modified it to work with unstructured, polyhedral meshes in OpenFOAM 4.1 using C++. Computation time varied depending on how fast fish exited the domain. A typical runtime for a single model run with n_{model} = 100 fish was about 85 s, using 8.1 GB RAM for up to 7200 time steps on one core of an Intel E5-2660-v3 ("Haswell") CPU.

2.5 Model function test

Besides behavioral rules, parameters (i.e., variables which do not change during a model run, but can change between model runs) are the second main component of a behavioral model. Our methods for testing their influence are described in this section before returning to our actual purpose of testing different stimuli in section 2.6.

2.5.1 Evaluation metric RMSE

To rate the quality of each model run, we computed pattern values P1-P5 in analogy to the laboratory patterns from n_{model} = 100 fish. As evaluation metric, we computed a weighted root-mean-square error (RMSE) between all model and laboratory pattern mean values (Table 1 below) for each model run as

$$RMSE_{model-run} = \sqrt{a_1\Delta P_1 + a_2\Delta P_2 + a_3\Delta P_3 + a_4\Delta P_4 + a_5\Delta P_5} \quad [-] \qquad \text{(Eq. 4)}$$

$$\text{Average of pattern P1-P3:} \qquad \Delta P_i = \frac{1}{3}\sum_j \left(P_{i,j,model} - P_{i,j,lab}\right)^2$$

$$\text{Pattern P4-P5:} \qquad \Delta P_i = \left(P_{i,model} - P_{i,lab}\right)^2$$

where a was a pattern weight factor chosen per pattern importance (a_1 = 0.3, a_2 = 0.1, $a_{3\text{-}5}$ = 0.2), i = 1..5 was a pattern iterator, and j = 1..3 was a pattern value iterator. As P1-P3 consisted of three pattern values each, these were averaged. A value of RMSE = 0 implies perfect agreement between model and laboratory results. We chose RMSE as it is both simple and penalizes large differences, which makes it useful for filtering outliers and achieving an overall good agreement.

The different weights of patterns P1 and P2 reflected their ~~estimated~~ expected importance for modeling orientation (section 2.2). We also tested equal weighting per pattern average value and equal weighting per pattern value, but obtained negligible effects on the stimulus version ranking. We focused on comparing the mean instead of SD values to reduce the complexity of model development, accepting that SD agreement cannot be evaluated.

2.5.2 Random seed sensitivity

The behavioral rules used pseudo-random numbers which were generated from a random seed number fixed for each model run. For identical seeds, results were identical. To minimize and to quantify ~~the~~ influence of the random seed choice, we tested ~~each~~ all parameter sets with N_r = 10 different seeds. Then, we averaged the resulting $RMSE_{model\text{-}run}$ values to obtain one RMSE value per parameter set. As seeds, we obtained ten true random numbers from www.random.org in the range from 1-999: 656, 36, 849, 934, 679, 758, 743, 392, 655, 171.

2.5.3 Parameter set generation

In total, the model had 7 fixed and D = 20 variable parameters (Table 2 in results and Table 3 in appendix S4, p. 8). The latter could be split into D_1 = 15 parameters for general movement, D_2 = 3 parameters for initialization, and D_3 = 1-2 parameters depending on the chosen stimulus version.

For sensitivity testing and stimulus version testing, we needed to generate a number of parameter sets with systematic variation in their parameter values. To ensure comparability and to save computational effort, we first included all parameters of the five stimulus versions and subsequently filtered the resulting parameter sets as required (Figure 3).

Values of an initial default parameter set were established by trial-and-error. For variation, we chose another 5 values in equal distances around the default value to test a total of p = 6 values for each parameter. The default values were either at the 3rd or 4th place of the range (appendix S4).

The full parameter space would comprise p^D parameter sets. We sampled it using the revised Morris method (Campolongo et al. 2007, r = 1000 random trajectories, T = 50 optimum trajectories) implemented in Python 3.7.4 with SALib 1.3.8. We obtained $n_{sets,base} = (D+1) \cdot T$ = 1050 systematically varying base parameter sets consisting of D parameter values each.

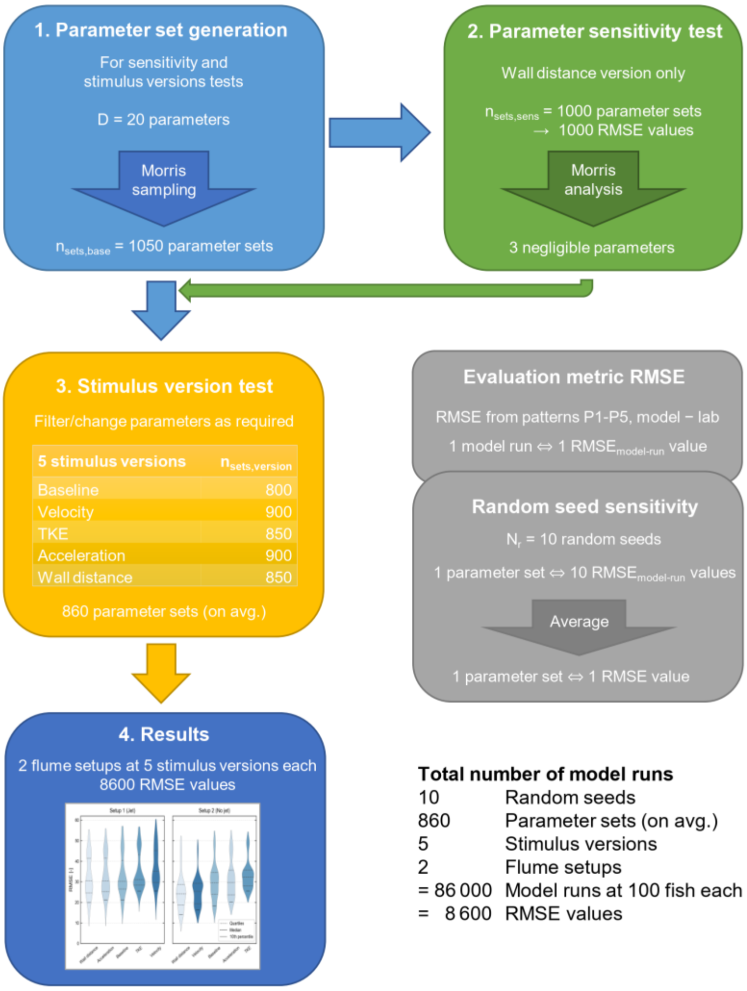

Figure 3: The process of testing our behavioral model. It starts with the generation of varying parameter sets, which are used for testing parameter sensitivity and contrasting stimulus versions in the following. Each box corresponds to one text section.

2.5.4 Parameter sensitivity test

To identify parameters of strong and negligible influence, we analyzed parameter sensitivity. As stimulus version for this investigation, we arbitrarily chose the wall distance in both flume setups. Thus, the acceleration/TKE stimulus threshold parameter was not required here. This reduced the number of parameter sets by T variations to $n_{sets,sens}$ = 1000 parameter sets. As described above, for each parameter set we ran the model with N_r random seeds and averaged the resulting $\text{RMSE}_{\text{model-run}}$ values.

Results were analyzed by means of revised Morris screening (Campolongo et al. 2007; Thiele et al. 2014). This method uses the effect of single parameter variations on the averaged RMSE to determine an influence measure, μ^*, and an interaction measure, σ_M, for each parameter. We used μ^* for ranking parameters by influence. For further interpretation, the values of the measures needed to be classified as "high" or "low". This is usually achieved graphically (e.g., Campolongo et al. 2007). We took a quantitative approach through normalizing μ^* and σ_M by their respective maximum values and, then, defining limits in dependency of gaps in our results. We neglected three parameters with "low" influence (see results) in the subsequent stimulus version tests.

2.6 Stimulus version test

2.6.1 Parameter influence

We focused on testing external stimuli, as they can be readily predicted. This is a necessary requirement for our long-term goal of improving fishway performance. To find the stimulus which best explained upstream orientation, we tested all five versions of the migrating behavioral rule in both setups. All other behavioral rules of the model (including behavior selection, holding, drifting, and vertical behavior) were kept constant (for a protocol, see Grimm and Railsback 2012). The latter were tested implicitly by means of the overall result, to which they contributed.

To eliminate the risk of favoring a stimulus by a certain calibration/parameter set ("overfitting"), we applied a large number of identical parameter sets to each of the stimulus versions. They were generated from the base parameter sets by filtering the negligible parameters identified in sensitivity analysis. Slight differences in the number of sets, n_{sets}, resulted aroseised from three stimulus-specific parameters:

- the wall detection range did only apply towas used only by the wall distance stimulus;
- the threshold factor did only apply towas used only by the velocity and TKE stimuli;
- the default angle for the wall distance stimulus was half the default angle of the velocity, TKE, and acceleration stimuli.

Theis procedure yielded an average of $n_{sets,avg}$ = 860 parameter sets per stimulus version (range 800–900, Figure 3). For each parameter set, we ran the model with N_r random seeds and averaged the resulting $RMSE_{model\text{-}run}$ values to obtain one RMSE value. From the combination of parameter sets ($n_{sets,avg}$ = 860), flume setups (n_{setups} = 2), and stimulus versions ($n_{stimuli}$ = 5), we received a total of 8600 RMSE values (Figure 3).

2.6.2 Ranking

Since sampling of the parameter sets is a random process and not optimized towards better performance results, it includes many unfavorable parameter sets that are not suitable to assess stimuli performance. Thus, a selection of the best model runs/RMSE values was necessary to find out if the different stimulus versions performed equally good or differed from each other. Based on the frequency distribution, we sought a threshold where a clear rejuvenation with regard to better values was visible,

while still enough RMSE values for a statistical valid comparison remained. We opted for the 10th percentile of RMSE values, which was then used to rank the stimulus versions.

We treated both setups separately, as setup 1 was more demanding on the model due to the more heterogeneous flow field and the slot geometry.

To evaluate if differences between the stimulus versions were statistically significant, we computed the *p*-values (Kruskal-Wallis test with post-hoc Nemenyi test) between the RMSE results (cut-off above 10th percentile) of the stimulus versions separately for both setups using SciPy 1.0.1 with scikit-posthocs 0.6.6.

Finally, to facilitate vivid understanding and discussion, we classified the versions qualitatively. The best version per setup was classified as "good", which was backed by comparing the RMSE of its best parameter set to absolute reference values. Versions differing significantly ($p < \alpha = 0.01$) from the best version were classified as "moderate" or "poor" under consideration of the 10th percentile. Versions not differing significantly were classified as "good".

3 Results

3.1 Movement patterns

Analysis of the movement patterns provided several insights into observed trout behavior (Table 1). Pattern P1 indicated~~s~~ that brown trout spent the major part of trial time close to the lateral walls and screen, i.e. in a rather small share of the flume width. P2 showed~~s~~ that fish swam close to the bottom almost exclusively. P3-P5 ~~are~~ were less distinct. Large SD values indicated wide individual differences in observed behavior for P1 and P3.

Brown trout start positions were distributed to the P1 left/middle/right zones 68 % - 4 % - 28 % (setup 1) and 46 % - 13 % - 42 % (setup 2). They spent an average of about 60 % of track duration without other fish nearby.

Table 1: Mean and standard deviation (SD) of brown trout patterns in the two flume setups.

Pattern	Flume ~~section~~zone		Setup 1 (jet)			Setup 2 (no jet)		
	Name (facing downstream)	Extent (m)	n	mean (%)	SD (%)	n	mean (%)	SD (%)
P1 Lateral distribution	Left	0.25	25	33	±37	24	38	±39
	Middle	2.00	25	10	±12	24	6	±10
	Right	0.25	25	57	±38	24	56	±40
P2 Vertical distribution	Surface	0.15	25	1	±2	24	4	±17
	Middle	0.30	25	1	±2	24	0	±1
	Bottom	0.15	25	99	±3	24	96	±17
P3 Turn ~~area~~zone	Upstream	3.25	9	24	±19	12	27	±17
	Middle	3.25	9	29	±17	12	40	±16
	Downstream	3.25	9	47	±16	12	33	±26
P4 Few turns	-	-	15	60	-	12	50	-
P5 n_D/n	-	-	25	84	-	24	79	-

Values rounded to the closest integer. n_D and n, number of fish at lines D and A.

3.2 Computational fluid dynamics model

CFD model results were in good agreement with acoustic Doppler velocimeter measurements from the laboratory (Gisen 2018). Advection dominated the flow field; boundary roughness influence was limited to a few centimeters in both setups (Figure 4). Absence of the jet in setup 2 considerably reduced mean velocity U_m, acceleration magnitude, and TKE on the right-hand flume side (facing downstream). The flow field of both setups was asymmetric in lateral direction. The bulk flow velocity was U_{bulk} = Q/(width*water depth) ≈ 0.67 m/s. In large parts of the jet region of setup 1, flow velocity was about U_m ≈ 0.8 m/s.

Commented [SCUB20]: 15. Table 1 – The general magnitude and relation of patterns appears to be nearly identical between set-ups. I would even doubt there is any statistical difference between values. This would indicate one of two possible failures in the experimental design: 1). The modifications to the laboratory set-up did not achieve a discernable change in behavior; or 2). The patterns do not adequately capture the behavioral changes caused by the modification to the set-up. Either way, since the authors do not provide any rationale as to what the set-up change was indented to do makes interpretation difficult.

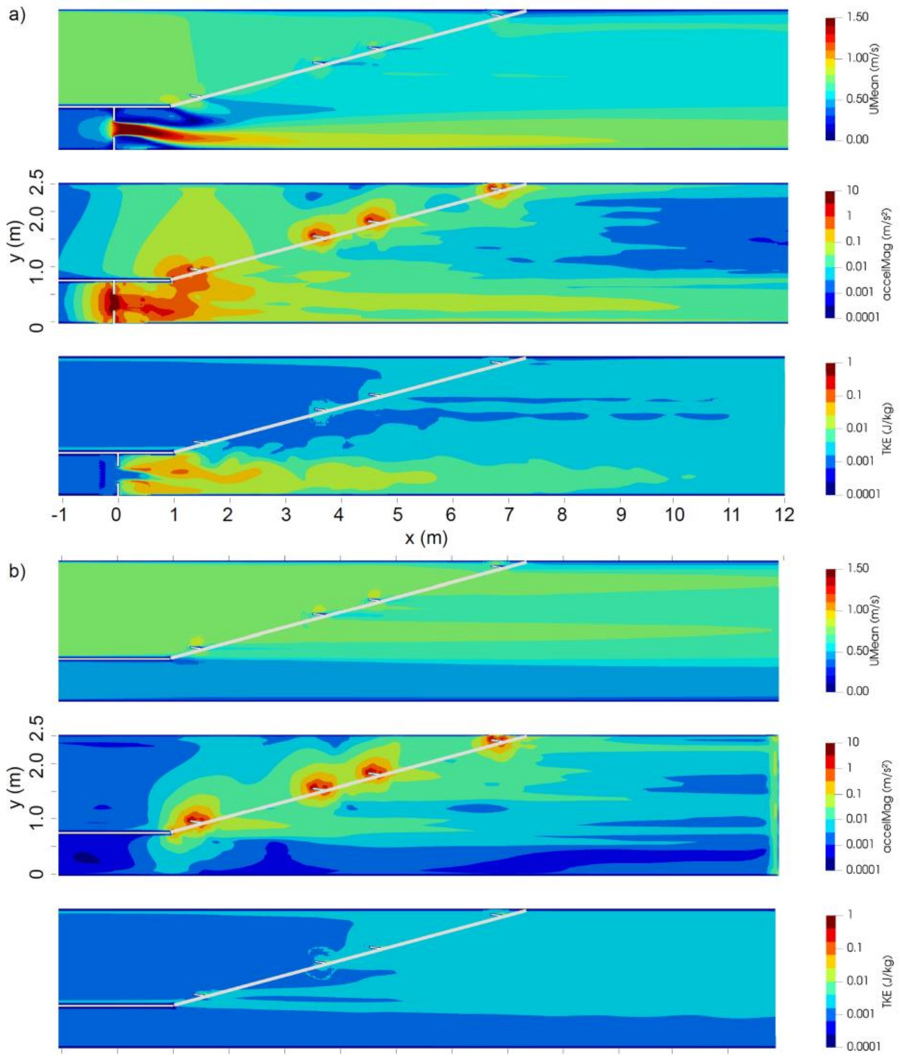

Figure 4: Horizontal flow field slices of setup 1 (a) and setup 2 (b) illustrate the effect of the slot and jet and its removal. Flow from left to right. Plane at z = 0.07 m above bottom where trout preferred to stay. Water depth = 0.60 m. Sub-panels in (a) and (b) show velocity magnitude U_m (top panels), acceleration magnitude (middle panels), and TKE (bottom panels). Note logarithmic scale for acceleration and TKE.

3.3 Random seed and time step sensitivity

To evaluate the influence of the different random seeds used, we compared the median coefficient of variation (σ/μ) for all ten setup/stimulus combinations. The values were low and ranged from 0.02-0.04, suggesting that our results were independent of the random seed chosen.

Halving the default time step to Δt = 0.25 s for the best wall distance parameter set deteriorated the RMSE in setup 1 and improved it in setup 2. Doubling the time step deteriorated the RMSE in both setups.

3.4 Parameter sensitivity

Parameter sensitivity was computed in both setups for the wall distance stimulus only. Setup 1 exhibited seven parameters of high influence (μ^*/μ^*_{max} > 0.6, Table 2), while setup 2 exhibited four parameters above this arbitrary limit. Ranks 17-19 comprised three parameters of low, negligible, effect on the result ($\mu^*/\mu^*_{max} \leq 0.1$). There was no first-order (independent) parameter (indicated by a high μ^* and low σ_M).

Both swim angles included in the migrating behavioral rule had a high influence and interaction in both setups. This result confirms that modeling orientation was important for the result. The vertical behavior correction angle was distinctly more important in setup 1 than in setup 2. This was likely caused by vertical currents induced by the plunging jet in setup 1.

All parameter sets which differed in one of the negligible parameters were excluded from the subsequent stimulus version tests. The parameter "wall detection range" was bound to the wall distance stimulus, anyway. The other two negligible parameters were not directly related to orientation, i.e. were expected to not interact strongly with the chosen stimulus. For the resulting number of parameter sets per stimulus version, n_{sets}, see Figure 3.

Table 2: Parameters of the behavioral model with wall distance stimulus, ranked by normalized influence, μ^*/μ^*_{max}, in setup 1. $\sigma_M/\sigma_{M,max}$ is normalized interaction with other parameters. The threshold for "low" values is indicated by a horizontal line. The last column, *No.*, is for reference to Table 3 in appendix S4, p. 8.

Setup 1 (Jet)			Parameter name	Setup 2 (No jet)			No.
Rank	μ^*/μ^*_{max}	$\sigma_M/\sigma_{M,max}$		Rank	μ^*/μ^*_{max}	$\sigma_M/\sigma_{M,max}$	
1	1.00	1.00	Migrating behavior max. random angle	2	0.74	0.60	14
2	0.82	0.87	Holding behavior extent	5	0.55	0.56	9
3	0.77	0.89	Migrating behavior wall distance angle	4	0.63	0.59	16
4	0.70	0.86	Fatigue (decreasing) memory coefficient	1	1.00	1.00	6
5	0.68	0.72	Vertical behavior correction angle	16	0.12	0.21	12
6	0.67	0.68	Spot memory coefficient	3	0.68	0.89	5
7	0.66	0.79	Motivation denominator ("fast fish")	7	0.47	0.69	3
8	0.56	0.64	Motivation memory coefficient	6	0.47	0.60	2
9	0.47	0.53	Stuck time threshold	10	0.22	0.34	13
10	0.41	0.46	Vertical behavior elevation threshold	13	0.13	0.17	11
11	0.33	0.41	Fatigue denominator	14	0.13	0.19	8
12	0.32	0.37	Motivation denominator ("slow fish")	9	0.27	0.37	4
13	0.24	0.34	Motivation initial value	11	0.21	0.30	1
14	0.24	0.29	Initialization "slow fish right"/"right fish"	15	0.13	0.17	20
15	0.21	0.20	Initialization "slow fish left"/"left fish"	12	0.13	0.34	19
16	0.20	0.23	Initialization "left fish"/"total fish"	8	0.44	0.44	18
17	0.09	0.10	Drifting behavior straight drift probability	17	0.10	0.23	10
18	0.07	0.07	Fatigue (increasing) memory coefficient	18	0.05	0.08	7
19	0.04	0.04	Migrating behavior wall detection range	19	0.02	0.02	15

3.5 Stimulus versions

We contrasted five stimulus versions for upstream swim angle selection in the model and ranked them (Table 3, Figure 5). The Kruskal-Wallis test obtained p = 16.2E-54 for setup 1 and p = 1.1E-78 for setup 2, suggesting that the stimulus versions did influence accordance with the laboratory observations significantly.

Table 3: Stimulus versions ranked by their RMSE 10th percentile, per setup (appendix S6). The p-value was calculated *post hoc* between wall distance and the particular stimulus version. Significant differences between these versions at $p < \alpha$ = 1.0E-02 are denoted by an asterisk (*). p-values are rounded to two significant digits, percentile values are rounded to integer.

Setup	Stimulus version	RMSE 10th percentile	*p*-Value	Rating
1 - Jet	Wall distance	20	-	good
	Acceleration	21	1.2E-01	good
	Baseline	21	4.0E-01	good
	TKE	25	*4.1E-30	poor
	Velocity	26	*2.7E-27	poor
2 - No jet	Wall distance	14	-	good
	Velocity	16	8.9E-02	good
	Baseline	18	*1.2E-12	moderate
	Acceleration	20	*9.5E-29	moderate
	TKE	26	*2.0E-60	poor

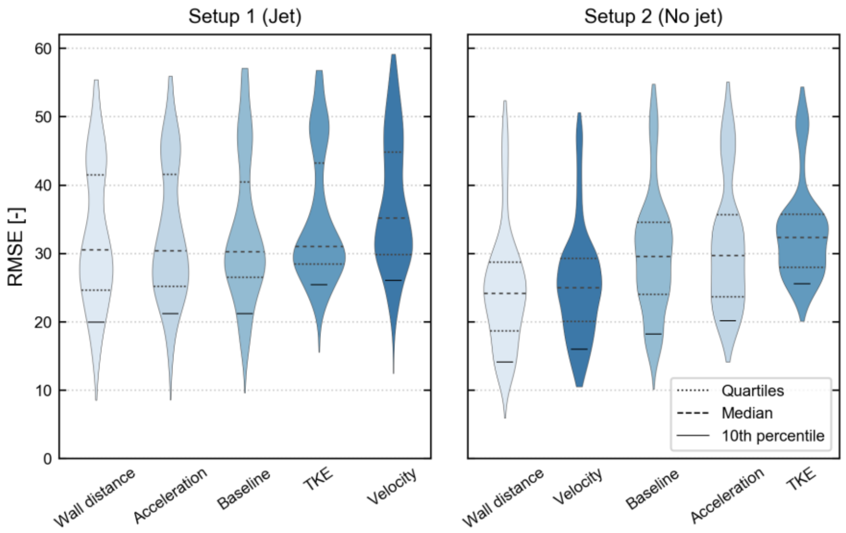

Figure 5: Violin plots indicating~~e~~ the frequency of root-mean-square error values (RMSE) in both setups (appendix S6). Ordering by increasing 10th percentile reveals similarities and significant differences between the stimulus versions. Results are better ~~the~~ if more values

are close to zero. The lower violin tip indicates the result of the best parameter set(s) for each stimulus version.

Wall distance was the best stimulus version in both setups. Acceleration and velocity differed widely in their ranks between setups, while baseline and TKE did not. In setup 1, the order of acceleration and baseline was not distinct, as well as the order of TKE and velocity (Figure 5). This is reflected in our qualitative "rating"/classification used for discussion (Table 3). The estimated distributions of the RMSE results are mainly multi-modal, which was the reason to rank them by their 10th percentile. The overall better results in setup 2 indicate that it was less demanding on the model than setup 1.

The wall distance version with its best parameter set reproduced all five patterns with high accuracy (RMSE = 9 for setup 1, RMSE = 6 for setup 2) (Figure 5, Figure 6). For comparison, e.g. Goodwin et al. (2014) aimed for RMSE ≤ 10. This finding shows that our model is able to produce all essential characteristics captured by our patterns (Grimm and Railsback 2005, p. 47).

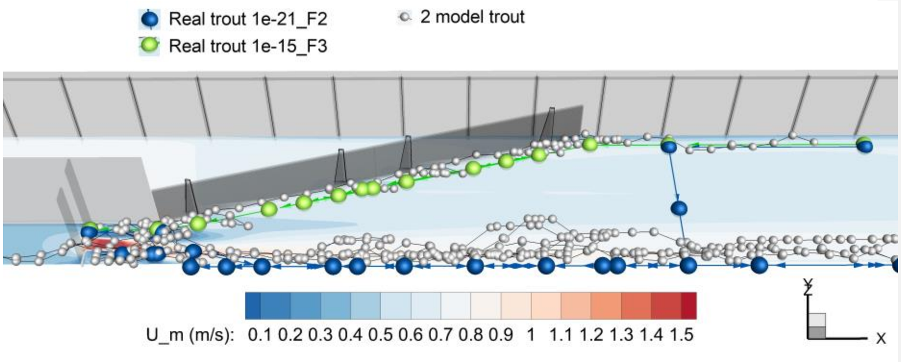

Figure 6: Two selected real and two selected model trout tracks in setup 1 (with jet). Each sphere shows one noted position or model time step, respectively. Model results are from the best parameter set of the wall distance stimulus. Plane shows mean velocity, U_m, at z = 0.07 m ~~from~~ above the bottom. Flow direction is from left to right, fish start from right on both flume sides. See appendices S1 and S2 for observed trout movement data.

Commented [SCUB21]: . Figure 6 – This figure was very helpful to understand the model results and laboratory data. Additional versions of this figure to compare the modelled movement against observed movement would be beneficial. Illustrations from both set-ups should be included.

4 Discussion

4.1 Stimuli for orientation

Commented [SCUB22]: Considering that the observed movement patterns were not largely different between the two set-ups, the conclusion that velocity does not play a role in orientation and navigation is stated too strongly. Based on the available data, it would appear that fish largely exhibited exploratory behavior and followed the walls because the hydraulics did not require any modified behavior. The authors should at least comment that their rule of avoiding high velocities is rather simple. It would not appear to fit the behavioral data either, since passage required movement through an area of elevated velocity in set-up 1. Perhaps a more refined guidance rule surrounding velocity fields is necessary.

In this work, we combined the very different fields of ethology, hydraulic engineering, and behavior modeling to approach a classical problem of behavioral ecology: How can orientation behavior be explained through external stimuli? Our resulting movement patterns, flow fields, and significant differences between the model stimulus versions allow to develop explanations with respect to the ambient conditions in our two setups.

Results for the baseline version ~~were~~ ranged from moderate in setup 1 to good in setup 2, indicating that rheotactic orientation and a random component may already explain not only orientation (pattern

P1), but also large parts of the remaining observed behavior captured by P2-P5. Positive rheotaxis was expected to be important as both ~~flow~~ velocity direction and observed movement directions were largely uniform and aligned, fostering this basic behavior (Coombs et al. 2020)~~(Arnold 1974)~~. Also, this result confirms that the random component was chosen in the right order to cover effects not modeled explicitly. Still, it was ~~surprising~~ unexpected that the other hydraulic variables commonly associated with behavior, i.e. velocity magnitude, TKE, and acceleration (e.g., Dockery et al. 2017; Goettel et al. 2015; Goodwin et al. 2014), were not required for this result. The apparent explanation that the flow field was not challenging enough to influence trout behavior will be discussed along the alternative stimulus versions tested.

The velocity model version yielded poor results in setup 1 and good results in setup 2. One reason for the failure in setup 1 was that model fish mostly avoided the jet of higher velocity on the right-hand side, while observed fish ~~did not~~frequented this zone in both setups (P1). The laboratory observations indicate that the tested trout did not aim to minimize their energy consumption by following low-velocity paths upstream as expected (Wang and Chanson 2018; Zielinski et al. 2018). Hence, the good model results in setup 2 are not the result of a real mechanism. They are rather a model effect of the low-velocity zone on the right-hand side, which fostered wall attraction and thus agreement to the observations. There is no apparent reason why trout would not seek lower velocity in setup 1 (with ~~even~~ larger bulk velocity) but would do so in setup 2. In conclusion, our model and laboratory results suggest that velocity was not a relevant stimulus for~~to~~ our trout.~~ This was surprising at first, but might be explained by the hydraulic conditions.~~.

To find an explanation, we used the body length as a proxy for swimming ability and~~To determine if a velocity/body length relation could explain why real trout ignored the higher velocity of the jet in setup 1, we made the following theoretical consideration~~considered the velocity/body length relation. A~~For~~ trout (BL = 0.27 m) holding station experienced a~~, the~~ relative flow velocity ~~was~~ of about U_{fish} = 3.0 BL/s in large parts of the jet in setup 1. At our water temperature, trout can maintain a sustained swimming speed of U_{fish} = 4.5 BL/s for up to 200 min according to Ebel's model equation for the rheophilic guild (Ebel 2014). Thus, in theory, trout could hold or~~have~~ move~~d~~ upstream while experiencing almost no fatigue (Webb 1975) for longer than the test duration, indicating that the flow velocity was indeed too low to trigger a reaction. Some support for this explanation comes from two studies, where low-velocity seeking was observed only at much larger relative velocities. Broadly estimated relative velocities were (0.37 m/s)/(0.07 m/BL) ≈ 5.3 BL/s for Duboulay's rainbowfish (Wang and Chanson 2018) and (0.39 m/s)/(0.06 m/BL) ≈ 6.5 BL/s for longnose dace (Dockery et al. 2017). In contrast, Kerr et al. (2016) reported low-velocity seeking already at (0.40 m/s)/(0.14 m/BL) ≈ 2.9 BL/s for small hatchery brown trout, which is similar to our relative velocity. ~~The latter study~~This discrepancy indicates ~~shows~~ that a general ~~explanation~~ movement hypothesis has ~~likely~~ to include more factors than just relative velocity.~~, even if it could be an explanation for our results.~~

Our next test for constant TKE attraction scored poor results in both setups. As both walls and bottom were smooth, they did not provide a distinct TKE stimulus for wall attraction, resulting in large deviations in P1 and the RMSE. These results challenge the statement that TKE is "by far the best stimulus" for fishway models (Gao et al. 2016) and the hypothesis that a constant turbulence level can be employed for

orientation (Goettel et al. 2015). We did not test attraction towards increasing or decreasing TKE, but would expect similarly poor results for P1 as the TKE field offers a ~~no~~ decent stimul~~u~~us only ~~s~~ towards ~~both~~ one side ~~at onces~~ (lateral asymmetry), ~~which makes it difficult to match the~~while ~~laboratory-observed~~the fish observed visited ~~distribution along~~ both sides.

Like for velocity, we compared TKE levels with literature values to determine if a relation to body length could explain our results. Our setups exhibited levels of TKE = 0.001-0.1 J/kg. Studies which explained behavior using TKE reported higher values for smaller fish: TKE = 0.1-0.3 J/kg for trout, BL = 0.20 m (Gao et al. 2016) and TKE = 0.015 J/kg for dace, BL = 0.07 m (Goettel et al. 2015). This qualitative comparison is in agreement with the explanation that the flow field did not cause behavioral reactions as it was not challenging for ~~our~~ trout. However, TKE is less studied for orientation than velocity and further research is required.

Results of the acceleration model version (good and moderate, respectively) were similar in absolute RMSE terms between setups, but differed widely in their in-setup rank because of better performances of the velocity and baseline versions in setup 2. Finding a theoretical explanation is difficult, as the few existing quantitative studies dealing with acceleration as an orientation stimulus focus on downstream movement (Kemp et al. 2006; Goodwin et al. 2014), which is associated with different responses than upstream movement. As for velocity (but with the better setup swapped), we suspect that a model effect, and not a real mechanism, is responsible for the different ranking: Acceleration amplifies the velocity gradient at the lateral walls and produces a distinct stimulus for model wall attraction in setup 1, but not in setup 2, thus failing to match P1 observations (which are similar in both setups). Again, the most probable explanation for our inconsistent results with acceleration, velocity, and TKE is that the live trout did not orient by means of the flow field (except for direction), but by other factors. The most relevant is non-hydraulic perception of wall distance.

Using the wall distance version, our model performed well in terms of absolute RMSE and significantly better than any alternative version in at least one setup. This result is in line with the assumed limited hydraulic influence on orientation in our conditions. ~~Without hydraulic influence, it is particularly evident that any gradient which~~As this model version is based on following a ~~stimulus~~gradient pointing towards the wall, it carries the risk of imposing (Grimm and Railsback 2005) large residence ~~times~~ durations near the walls~~in the near-wall zones of pattern P1~~. This risk is mitigated by two mechanisms for moving ~~to the middle zone~~away from the wall, namely the random overlay angle and inclined drift. The good RMSE results for this version suggest that these mechanisms are in the right balance~~. We conclude~~ and that wall attraction by distance was a real behavior. ~~Multiple reasons are possible~~Potential reasons includ~~eing~~ guidance, cover, or hydraulic advantages from the walls, but further experiments are required to evaluate ~~them~~their probability.

For the physiological mechanism of wall attraction, multiple explanations are conceivable. To us, visual distance estimation is the best explanation, considering that vision is a predominant stimulus in fish (Douglas and Hawryshyn 1990; Liao 2007; Elder and Coombs 2015). Given the clear visibility of the walls, it seems unlikely that another potential far-field stimulus (e.g. acoustic) dominated distance estimation. Distance estimation by means of a hydraulic signature is also unlikely according to our CFD model results;~~.~~

a~~A~~s discussed before, they show that wall influence was limited to some centimeters distance and/or was inconsistent between setups in ~~flow~~ hydraulic variables (velocity, TKE, acceleration) which are commonly associated with behavioral responses (e.g., Dockery et al. 2017; Goettel et al. 2015; Goodwin et al. 2014). As a study on blind cave fish shows, hydrodynamic imaging by the lateral line close to smooth walls is limited to a small fraction of the body length (Windsor et al. 2010).

Taken together, our results indicate that the rarely considered wall distance can be a relevant stimulus for orientation in fishways and similarly confined migration paths. It requires, of course, ambient conditions which allow its perception, ~~by~~ e.g. clear and light water ~~for visibility~~. Further, it~~by the visual or acoustic sense~~ ~~and~~ may be overruled by hydraulic stimuli ~~like~~ . ~~V~~velocity and TKE if they are relatively larger ~~stimuli being small~~ (in relation to fish ~~body length~~swimming ability) than- in our ~~model~~conditions~~could pose another necessary condition~~.

4.2 Stimulus combinations

In our model, we limited orientation stimuli to the baseline ~~flow~~ velocity direction and one additional stimulus at a time. This is a simplification of reality, where most complex behavior is unlikely to depend on a single stimulus/sense alone (Liao 2006; Goettel et al. 2015), and is more likely to have a polysensory background (Kasumyan 2011). While making a model more realistic is not the same as making it more useful, combining stimuli to act on the same behavior is a worthwhile research direction for improving the generality of fish orientation IBMs.

A recent example is the use of calibrated weight factors for three hydraulic stimuli~~, which produced qualitative agreement to three carp tracks~~ in an upstream orientation IBM for carp (Tan et al. 2018). A ~~step~~ further step would be to model shifting influence of stimuli depending on the ambient conditions, which was shown to exist in different species (Elder and Coombs 2015; Fuentes-Pérez et al. 2018). However, ~~in our own~~~~tweaking~~adapting our model~~, a combination o~~ to use e.g.~~f~~ ~~the second-ranked~~ acceleration ~~version for orientation~~ in setup 1 and ~~the second-ranked~~ velocity ~~version for orientation~~ in setup 2 would still yield results not ~~be~~ as good as the ~~first-ranked~~ wall distance version (two times rank 2 vs. two times rank 1). In addition, both stimuli were likely not real stimuli (as discussed above) and hence such a model version would reduce robustness and explanatory power for our experimental conditions. Therefore, we decided not to ~~re-calculate~~run our model with stimuli combinations~~, but would consider this to be important for~~ ~~We conclude that our current model is not likely improved through stimuli combinations, but they could be important for transfer to more complex environments such as in-situ fishways.~~.

Commented [SCUB23]: 18. Line 530-532 – The rationale for not running the model with combinations of stimuli is lacking. I can understand the reason for not including this step in this study, but to dismiss their validity is premature. The authors do not explicitly indicate how multiple stimuli could be integrated into a single model, so I do not understand how they can claim the acceleration and velocity versions could not improve upon the wall distance version without explicitly testing this.

4.3 Movement patterns in the laboratory

Transfer of our model also depends on how universal the underl~~a~~ying movement patterns are. The observed tendency to swim close to the walls (pattern P1) matched descriptions of rainbow trout behavior (Przybilla et al. 2010) and barbel behavior in a confined model fishway (Fuentes-Pérez et al. 2018). It was not observed in faster flow and a more narrow flume for brook and brown trout (Castro-

Santos et al. 2013). This discrepancy indicates a potential dependence on the ambient conditions. For our slow, shallow flow, we conclude that P1 is not an untypical behavior.

Swimming at the bottom is reported frequently, e.g. in a natural stream for brown trout (Cotel et al. 2006), and in flumes for rainbow trout (Przybilla et al. 2010), barbels (Silva et al. 2012), brown and brook trout (Castro-Santos et al. 2013), and longnose dace (Dockery et al. 2017). The water column in our experiment was shallow (d = 0.60 m), hence it was unclear whether fish preferred the bottom for orientation, for shelter, or for another reason. Despite possible behavioral or physiological reasons, P2 can be considered a typical behavior in our flume conditions.

For patterns P3 and P4, we did not find matching descriptions in the literature, because they represent a new way of systematically dealing with the striking feature "turns". P5 was also too specific to our setup~~ for a meaningful comparison~~.

From the literature comparisons, it seems plausible that patterns P1 and P2 would be universal enough to support model transfer to similar flows, e.g. to an altered flume geometry. Transfer to the real world could be challenging, e.g. due to limited wall distance estimation and/or larger flow velocities.

4.4 General limitations

Finally, we want to point out some general limitations of this work to facilitate critical reception. First, our results are limited in scale to the estimated fish observation accuracy, which was coarser than the scale of changes in the hydraulic variables in a distance of 1-2 m downstream of the slot. Thus, potential effects of sudden changes in this area on the behavior could not be incorporated into the model.

Second, we found that different parameter combinations produced similarly good results, which indicates that parameter values are not robust and transfer of the behavioral model to other species and/or hydraulic environments requires recalibration. This does not affect our reproducible testing procedure of complex hypotheses, which is one of the major assets of IBMs (Willis 2011).

Third, the behavioral model was sensitive to changes in time step width. Main influences changing with the number of time steps include accuracy of flow field perception (Smith et al. 2014) and the number of orientation and random decisions.

Fourth, matching P1 depended, among other effects, on the initial distribution of fish positions to either side. The share of fish starting on the left flume side in the best wall distance version was off the real distributions observed in the flume (33 % vs. 68 % with jet and 33 % vs. 46 % without jet). Producing matching results from differing initial conditions could point to a difference between model and laboratory behavior or it could be a sign of minor relevance of the initial conditions to the model result.

Fifth, our model is currently limited to steady flow fields. Modeling e.g. utilization of transient vortices would require considerable model modification and computational resources (Padgett et al. 2020).

Sixth and last, although swimming in groups (shoaling) was not pronounced in our data set and not modeled, it can influence orientation behavior. Determining its influence would require laboratory experiments with single fish releases. ~~Points one to four are specific to this very data set and model instance, but point five and especially point six could improve future development of our IBM.~~

5 Conclusions

Our goal in this study was to better understand upstream fish orientation and navigation in confined space, e.g. in fishways, by means of concrete behavioral rules. Our thoroughly tested IBM was able to reproduce five ~~brown trout behavioral~~ movement patterns of brown trout in two experimental flow fields, which represented a broad range of ~~spatial~~ behavior in a wide flume. The first two patterns – ~~avoiding the middle and surface~~preferring wall and bottom proximity ~~zones of the flume~~ – were very distinct, and should be investigated further to determine their dependency on e.g. water width and depth.

The significant advantage of the wall distance stimulus version illustrates that a focus on hydraulic stimuli for predicting fish orientation can be too narrow, especially in hydraulic conditions not ~~demanding~~ challenging for the observed individuals. These can occur e.g. for strong swimmers in multi-species fishways. In such conditions, particularly ~~in confined space with visible walls~~when the walls are visible, w~~W~~all distance ~~estimation, mediated by whatever sense,~~ should be considered more frequently as a stimulus ~~in confined space. This is true for studies~~ in IBMs, laboratory ~~flumes~~experiments, ~~studies,~~ and ~~in the~~ field studies. ~~if the conditions enable wall perception by e.g.~~ ~~the visual or acoustic sense.~~

The evaluation of observed patterns and key parameter variety indicates~~d~~ that transfer of our present model is likely limited to similar species and conditions, i.e. brown trout of a given size in a relatively slow, shallow flume flow. This does not affect the IBM's ability to contrast different behavior hypotheses.

In summary, the interdisciplinary IBM technique facilitates ~~the investigation of hypotheses for stimuli-response mechanisms~~testing of behavioral rules ~~for~~ using alternative ~~external~~ stimuli with flexibility and rigor. It is an important option to approach the many urgent questions of fishway designers.

6 Acknowledgments

We thank all persons involved in the realization of the brown trout movement experiments.

We thank Dr. Andy Goodwin of USACE for providing the Fortran 90 model code used in Goodwin et al. (2014) as a starting point.

Commented [SCUB24]: . Line 589 – The authors repeatedly indicate that wall perception could be accomplished by vision or audition, but it is not entirely clear how or what role audition may play in wall distance perception. I would still expect near field hydrodynamics would override any true acoustical input for most fish. This just strikes me as an odd statement and should be further explained if it remains in the manuscript.

7 References

Arenas, Antonio; Politano, Marcela; Weber, Larry; Timko, Mark (2015): Analysis of movements and behavior of smolts swimming in hydropower reservoirs. In *Ecological Modelling* 312, pp. 292–307. DOI: 10.1016/j.ecolmodel.2015.05.015.

Arnold, G. P. (1974): Rheotropism in fishes. In *Biological Reviews* 49 (4), pp. 515–576. DOI: 10.1111/j.1469-185X.1974.tb01173.x.

Bleckmann, Horst; Zelick, Randy (2009): Lateral line system of fish. In *Integrative zoology* 4 (1), pp. 13–25. DOI: 10.1111/j.1749-4877.2008.00131.x.

Bunt, C. M.; Castro-Santos, T.; Haro, A. (2016): Reinforcement and Validation of the Analyses and Conclusions Related to Fishway Evaluation Data from Bunt et al.: 'Performance of Fish Passage Structures at Upstream Barriers to Migration'. In *River Res. Applic.* 32 (10), pp. 2125–2137. DOI: 10.1002/rra.3095.

Bunt, Christopher M.; Castro-Santos, Theodore; Haro, Alex (2012): Performance of fish passage structures at upstream barriers to migration. In *River Res. Applic.* 28 (4), pp. 457–478. DOI: 10.1002/rra.1565.

Campolongo, Francesca; Cariboni, Jessica; Saltelli, Andrea (2007): An effective screening design for sensitivity analysis of large models. In *Environmental Modelling & Software* 22 (10), pp. 1509–1518. DOI: 10.1016/j.envsoft.2006.10.004.

Castro-Santos, Theodore (2004): Quantifying the combined effects of attempt rate and swimming capacity on passage through velocity barriers. In *Can. J. Fish. Aquat. Sci.* 61 (9), pp. 1602–1615. DOI: 10.1139/f04-094.

Castro-Santos, Theodore; Sanz-Ronda, Francisco Javier; Ruiz-Legazpi, Jorge; Jonsson, Bror (2013): Breaking the speed limit — comparative sprinting performance of brook trout (Salvelinus fontinalis) and brown trout (Salmo trutta). In *Can. J. Fish. Aquat. Sci.* 70 (2), pp. 280–293. DOI: 10.1139/cjfas-2012-0186.

Chapman, Ben B.; Hulthén, Kaj; Blomqvist, David R.; Hansson, Lars-Anders; Nilsson, Jan-Åke; Brodersen, Jakob et al. (2011): To boldly go. Individual differences in boldness influence migratory tendency. In *Ecology letters* 14 (9), pp. 871–876. DOI: 10.1111/j.1461-0248.2011.01648.x.

Coombs, Sheryl; Bak-Coleman, Joe; Montgomery, John (2020): Rheotaxis revisited. A multi-behavioral and multisensory perspective on how fish orient to flow. In *The Journal of experimental biology* 223 (Pt 23). DOI: 10.1242/jeb.223008.

Cotel, Aline J.; Webb, Paul W.; Tritico, Hans M. (2006): Do Brown Trout Choose Locations with Reduced Turbulence? In *Transactions of the American Fisheries Society* 135 (3), pp. 610–619. DOI: 10.1577/T04-196.1.

Dockery, D. R.; McMahon, T. E.; Kappenman, K. M.; Blank, M. (2017): Evaluation of swimming performance for fish passage of longnose dace Rhinichthys cataractae using an experimental flume. In *Journal of fish biology* 90 (3), pp. 980–1000. DOI: 10.1111/jfb.13217.

Douglas, Ron; Hawryshyn, Craig (1990): Behavioral studies of fish vision: An analysis of visual capabilities. In Ron Douglas, Mustafa Djamgoz (Eds.): The Visual System of Fish. Dordrecht: Springer Netherlands, pp. 373–418.

Ebel, Guntram (2014): Modellierung der Schwimmfähigkeit europäischer Fischarten — Zielgrößen für die hydraulische Bemessung von Fischschutzsystemen. In *Wasserwirtschaft* 104 (7-8), pp. 40–47. DOI: 10.1365/s35147-014-1095-1.

Elder, John; Coombs, Sheryl (2015): The influence of turbulence on the sensory basis of rheotaxis. In *Journal of comparative physiology. A, Neuroethology, sensory, neural, and behavioral physiology* 201 (7), pp. 667–680. DOI: 10.1007/s00359-015-1014-7.

Fuentes-Pérez, Juan Francisco; Eckert, Mario; Tuhtan, Jeffrey A.; Ferreira, Maria Teresa; Kruusmaa, Maarja; Branco, Paulo (2018): Spatial preferences of Iberian barbel in a vertical slot fishway under variable hydrodynamic scenarios. In *Ecological Engineering* 125, pp. 131–142. DOI: 10.1016/j.ecoleng.2018.10.014.

Gao, Zhu; Andersson, Helge I.; Dai, Huichao; Jiang, Fengjian; Zhao, Lihao (2016): A new Eulerian–Lagrangian agent method to model fish paths in a vertical slot fishway. In *Ecological Engineering* 88, pp. 217–225. DOI: 10.1016/j.ecoleng.2015.12.038.

Gisen, David C. (2018): Modeling upstream fish migration in small-scale using the Eulerian-Lagrangian-agent method (ELAM). Dissertation. Universität der Bundeswehr München, Munich, Germany. Available online at https://hdl.handle.net/20.500.11970/105158.

Goettel, Michael T.; Atkinson, Joseph F.; Bennett, Sean J. (2015): Behavior of western blacknose dace in a turbulence modified flow field. In *Ecological Engineering* 74, pp. 230–240. DOI: 10.1016/j.ecoleng.2014.10.012.

Goodwin, Richard Andrew; Politano, M.; Garvin, J. W.; Nestler, J. M.; Hay, D.; Anderson, James J. et al. (2014): Fish navigation of large dams emerges from their modulation of flow field experience. In *Proceedings of the National Academy of Sciences* 111 (14), pp. 5277–5282. DOI: 10.1073/pnas.1311874111.

Grimm, Volker; Berger, Uta; Bastiansen, Finn; Eliassen, Sigrunn; Ginot, Vincent; Giske, Jarl et al. (2006): A standard protocol for describing individual-based and agent-based models. In *Ecological Modelling* 198 (1-2), pp. 115–126. DOI: 10.1016/j.ecolmodel.2006.04.023.

Grimm, Volker; Railsback, Steven F. (2005): Individual-based modeling and ecology. Princeton: Princeton University Press (Princeton series in theoretical and computational biology).

Grimm, Volker; Railsback, Steven F. (2012): Pattern-oriented modelling: a 'multi-scope' for predictive systems ecology. In *Philosophical transactions of the Royal Society of London. Series B, Biological sciences* 367 (1586), pp. 298–310. DOI: 10.1098/rstb.2011.0180.

Grimm, Volker; Railsback, Steven F.; Vincenot, Christian E.; Berger, Uta; Gallagher, Cara; DeAngelis, Donald L. et al. (2020): The ODD Protocol for Describing Agent-Based and Other Simulation Models. A Second Update to Improve Clarity, Replication, and Structural Realism. In *JASSS* 23 (2). DOI: 10.18564/jasss.4259.

Jones, M. J.; Baumgartner, L. J.; Zampatti, B. P.; Beyer, K. (2017): Low light inhibits native fish movement through a vertical-slot fishway. Implications for engineering design. In *Fish Manag Ecol* 24 (3), pp. 177–185. DOI: 10.1111/fme.12205.

Kasumyan, A. O. (2011): Tactile reception and behavior of fish. In *J. Ichthyol.* 51 (11), pp. 1035–1103. DOI: 10.1134/S003294521111004X.

Kemp, P. S. (2016): Meta-analyses, Metrics and Motivation: Mixed Messages in the Fish Passage Debate. In *River Res. Applic.* 32 (10), pp. 2116–2124. DOI: 10.1002/rra.3082.

Kemp, Paul S.; Gessel, Michael H.; Sandford, Benjamin P.; Williams, John G. (2006): The behaviour of Pacific salmonid smolts during passage over two experimental weirs under light and dark conditions. In *River Res Applic* 22 (4), pp. 429–440. DOI: 10.1002/rra.913.

Kerr, James R.; Manes, Costantino; Kemp, Paul S. (2016): Assessing hydrodynamic space use of brown trout, Salmo trutta, in a complex flow environment: a return to first principles. In *Journal of Experimental Biology* 219 (Pt 21), pp. 3480–3491. DOI: 10.1242/jeb.134775.

Lacey, J. R. W.; Neary, V. S.; Liao, J. C.; Enders, E. C.; Tritico, H. M. (2012): The IPOS framework: linking fish swimming performance in altered flows from laboratory experiments to rivers. Special Issue Paper. In *River Res. Applic.* 28 (4), pp. 429–443. DOI: 10.1002/rra.1584.

Liao, James C. (2006): The role of the lateral line and vision on body kinematics and hydrodynamic preference of rainbow trout in turbulent flow. In *Journal of Experimental Biology* 209 (20), pp. 4077–4090. DOI: 10.1242/jeb.02487.

Liao, James C. (2007): A review of fish swimming mechanics and behaviour in altered flows. In *Philosophical Transactions of the Royal Society B: Biological Sciences* 362 (1487), pp. 1973–1993. DOI: 10.1098/rstb.2007.2082.

Montgomery, J.; Carton, G.; Voigt, R.; Baker, C.; Diebel, C. (2000): Sensory processing of water currents by fishes. In *Philosophical Transactions of the Royal Society B: Biological Sciences* 355 (1401), pp. 1325–1327. DOI: 10.1098/rstb.2000.0693.

Padgett, Thomas E.; Thomas, Robert E.; Borman, Duncan J.; Mould, David C. (2020): Individual-based model of juvenile eel movement parametrized with computational fluid dynamics-derived flow fields informs improved fish pass design. In *Royal Society open science* 7 (1), p. 191505. DOI: 10.1098/rsos.191505.

Przybilla, Anja; Kunze, Sebastian; Rudert, Alexander; Bleckmann, Horst; Brücker, Christoph (2010): Entraining in trout: a behavioural and hydrodynamic analysis. In *Journal of Experimental Biology* 213 (Pt 17), pp. 2976–2986. DOI: 10.1242/jeb.041632.

Rodríguez, Álvaro; Bermúdez, María; Rabuñal, Juan R.; Puertas, Jerónimo (2015): Fish tracking in vertical slot fishways using computer vision techniques. In *Journal of Hydroinformatics* 17 (2), pp. 275–292. DOI: 10.2166/hydro.2014.034.

Roth, Márcio S.; Jähnel, Christian; Stamm, Jürgen; Schneider, Lisa K. (2021): Turbulent eddy identification of a meander and vertical-slot fishways in numerical models applying the IPOS-framework. In *Journal of Ecohydraulics* 21 (6), pp. 1–20. DOI: 10.1080/24705357.2020.1869916.

Schütz, Cornelia; Henning, Martin; Czerny, Rebekka; Herbst, Marcus; Pitsch, Matthias (2021): Addition of auxiliary discharge into a fishway - A contribution to fish pass design at barrages of large rivers. In *Ecological Engineering* 167. DOI: 10.1016/j.ecoleng.2021.106257.

Silva, Ana T.; Katopodis, Christos; Santos, José M.; Ferreira, Maria Teresa; Pinheiro, António N. (2012): Cyprinid swimming behaviour in response to turbulent flow. In *Ecological Engineering* 44, pp. 314–328. DOI: 10.1016/j.ecoleng.2012.04.015.

Silva, Ana T.; Lucas, Martyn C.; Castro-Santos, Theodore; Katopodis, Christos; Baumgartner, Lee J.; Thiem, Jason D. et al. (2018): The future of fish passage science, engineering, and practice. In *Fish and Fisheries* 19 (2), pp. 340–362. DOI: 10.1111/faf.12258.

Smith, David L.; Goodwin, Richard Andrew; Lai, Young; Nestler, John Michael (2014): Fish path selection and fatigue in complex, continuous velocity fields. In Atle Harby, Håkon Sundt (Eds.): 10th International Symposium on Ecohydraulics. 10th International Symposium on Ecohydraulics. Trondheim.

Tan, Junjun; Tao, Lin; Gao, Zhu; Dai, Huichao; Shi, Xiaotao (2018): Modeling Fish Movement Trajectories in Relation to Hydraulic Response Relationships in an Experimental Fishway. In *Water* 10 (11). DOI: 10.3390/w10111511.

The OpenFOAM Foundation (2014): OpenFOAM. Version 2.3.1. Available online at https://github.com/OpenFOAM/OpenFOAM-2.3.x, checked on 4/28/2020.

Thiele, Jan C.; Kurth, Winfried; Grimm, Volker (2014): Facilitating Parameter Estimation and Sensitivity Analysis of Agent-Based Models. A Cookbook Using NetLogo and 'R'. In *JASSS* 17 (3). DOI: 10.18564/jasss.2503.

Wang, H.; Chanson, H. (2018): Modelling upstream fish passage in standard box culverts. Interplay between turbulence, fish kinematics, and energetics. In *River Res. Applic.* 128 (5), p. 245. DOI: 10.1002/rra.3245.

Webb, Paul W. (1975): Hydrodynamics and Energetics of Fish Propulsion. Edited by J. C. Stevenson. Fisheries Research Board of Canada. Ottawa, Canada (Bulletin of the Fisheries Research Board of Canada, 190). Available online at https://scholarworks.umass.edu/fishpassage_journal_articles/569.

Willis, Jay (2011): Modelling swimming aquatic animals in hydrodynamic models. In *Ecological Modelling* 222 (23-24), pp. 3869–3887.

Windsor, Shane P.; Norris, Stuart E.; Cameron, Stuart M.; Mallinson, Gordon D.; Montgomery, John C. (2010): The flow fields involved in hydrodynamic imaging by blind Mexican cave fish (Astyanax fasciatus). Part II: Gliding parallel to a wall. In *The Journal of experimental biology* 213 (22), pp. 3832–3842. DOI: 10.1242/jeb.040790.

Zielinski, D. P.; Voller, VR.; Sorensen, P. W. (2018): A physiologically inspired agent-based approach to model upstream passage of invasive fish at a lock-and-dam. In *Ecological Modelling* 382, pp. 18–32. DOI: 10.1016/j.ecolmodel.2018.05.004.

8 Supporting information appendix

8.1 S1 Brown trout movement observations, setup 1

Unpublished movement data from Schütz et al. (2021). See S1_brown_trout_setup1.xlsx.

Column name	Definition	Units
fish_id	"1e" is setup 1; "2c" is setup 2. Next part of the ID is trial number, and last is fish number (3 per trial).	-
time_stamp	Time of observation, interpolated from minutes to seconds by event count per minute	UTC DD.MM.YYYY HH:MM:ss
sec	Seconds since start (lifting of staging area mesh screen)	s
swarmsize	Size of group observed, 1-3 fish, "group" was defined subjectively by swimming together	-
x_raw	Longitudinal position observed, axis zero at flume start	m
y_raw	Lateral position, observed to be close to left wall/screen (L), right wall/screen (R), or close to neither wall/screen (M). Used for pattern P1.	Categorical
z_raw	Vertical position observed in the water column, either close to water surface (o), close to bottom (u), or in between (m). Used for pattern P2.	Categorical
x_value	Longitudinal position, computed in postprocessing, axis zero at slot. Used for patterns P3, P4, and P5.	m
y_value	Lateral position, computed in postprocessing, defined from y_raw as being either 0.1 m away from a wall/screen or in the middle. Coordinates account for inclined screen. Used only for computation of cells in Figure 2, not for determining pattern P1.	m

8.2 S2 Brown trout movement observations, setup 2

See S2_brown_trout_setup2.xlsx. Description above.

8.3 S3 Hydraulic field data

See S3_hydraulic_fields.zip containing S3a_hydraulic_fields_setup1.csv and S3b_hydraulic_fields_setup2.csv. For coordinate system origin, see Figure 1.

Column name	Description	Units
ccx, ccy, ccz	Cell center coordinates	m
u, v, w	Velocity vector components	m/s
acclMag	Acceleration magnitude	m/s^2
alpha.water	Water phase, share of cell volume	-
k	Turbulence kinetic energy (TKE)	m^2/s^2

8.4 S4 Behavioral model description

See S4_Gisen_behavioral_model_description.pdf

8.5 S5 Behavioral model code

See https://github.com/baw-de/ELAM-flume. The code is written in C++ and Fortran 90. The Fortran code uses the FORTRAN 77 fixed format for historic reasons.

8.6 S6 Root mean square errors

See S6_RMSE_5_stimuli_2_setups.csv. The order is Wall distance (setup 1, setup 2) – Acceleration 1, 2 – Velocity 1, 2 – TKE 1, 2 – Baseline 1, 2.

Response to reviewers

First, we would like to thank the reviewers for their insightful comments, which helped us to improve the manuscript. Our particular responses can be found below. Generally, we tried to further streamline terms, correct errors, and to improve readability throughout the whole manuscript.

We note that the manuscript does not yet meet the style requirements of PLOS ONE, but decided to await the provisional Editorial Accept decision (per https://journals.plos.org/plosone/s/getting-started) to avoid confusion during review e.g. by changing the citation style.

Reviewer #1

Comment	Addressed?	How addressed or why not?	Line (original)
1. The introduction is poorly organized and confusing. The discussion of hydraulic parameters is insufficient and unclear.	Yes	We've rewritten large parts of the introduction, focusing on readability and the motivation for our study. We've also deleted information on stimuli that went too much into detail or - where helpful - moved them to the model description to provide a rationale on why each stimulus was chosen.	
2. Why would learning effects matter in this scenario?	No	Fish already familiar with the flume geometry and hydraulic field could deviate in their behavior unpredictably, e.g. navigate straight towards the upstream end or don't move at all. Excluding such effects by testing every individual just once makes results more reproducible.	110
3. I don't understand why the fish swimming study was designed the way it was. Was it specifically designed to calibrate/validate the model?	Yes	(See also comments #9 and #15 of reviewer #3.)<br>The previous, unrelated study was primarily designed to investigate the influence of auxiliary water velocity coming from the screen on passage success and delay of five species.<br>Conditions were chosen to create hydraulic conditions typical for a large multi-species fishway in Germany. It is now published in Ecological Engineering, where more information is available:<br>https://doi.org/10.1016/j.ecoleng.2021.106257<br>We used the both setups to establish an application range for our model, see also comments #9 and #15 of reviewer #3.<br>We've added information on design choice and the word "unrelated" in section 2.1 for clarification.	100
4. I cannot provide a good review of the movement patterns section (2.2) because it is not something I've studied.	-	-	-

5. Please provide more information on CFD model setup, calibration and validation.	No	We feel the information provided on the model setup is sufficient for reproduction. Calibration in the classical sense was not performed, as boundary conditions are fully determined by the laboratory flume. Mesh, turbulence model, and screen negligence were validated using ADV probe data (line 362f). Comprehensive detail information can be found in (Gisen 2018, p. 33ff.).	153ff
6. I would like more explanation for why decisions were made ....e.g. why use averaged/steady results from the CFD model?	Yes	Using transient CFD results is a promising direction to capture behavior currently modelled stochastically. However, interpolating thousands of 3D fish positions on 7200 flow field snapshots (dt = 0.5 s for 1 h) is currently far beyond our simulation capacity, even using a high-performance cluster. We've added a brief mention to the limitations section 4.4.	
7. I love this stuff and I think its interesting work, I just want more information in the methods that is clearly organized and suggest a full rewrite where you focus on streamlining terminology, writing good paragraphs, and correcting grammar errors.	Yes	We've added information to the methods section and tried to streamline terminology in the whole manuscript. We considered to change the order of subsections in section 2.5, but refrained from doing so, as there are too many dependencies in both ways, e.g. between RMSE, RMSE(model-run), and parameter set evaluation. We rely on figure 3 to explain these dependencies.	
8. I'm a bit concerned that it is unclear how much of this work is Goodwin's and how much is new work.	Yes	We've tried to clarify in the introduction that Goodwin (2014) worked on downstream migration on dam-scale, while we work on upstream migration on fishway-scale, which required e.g. a completely new behavioral model. The CFD model was also replaced, of course, and the software framework was heavily modified (section 2.4.1).	

Reviewer #2

Reviewer #2: "*Nicely thought out work. Please see manuscript PDF where I mention a few places where better word choice will help the reader. More broadly, I think the manuscript could be improved in terms of readability by improving the flow of information. Some detail and terms within the manuscript make it hard to follow at times, so perhaps consider moving some technical pieces/info together (or use more general terms where possible) to allow more portions of the manuscript to flow (read) easier.*"

**Comment**	**Addressed?**	**How addressed or why not?**	**Line (original)**
1. Unclear, please restate another way.	Yes	Rephrased	50
2. Unclear.	Yes	Replaced "like for P1" with "using Eq. 1"	136
3. Unclear, please restate another way.	Yes	Rephrased	321

4. Unclear, please restate another way.	Yes	Rephrased	322
5. underlying ?	Yes	Deleted “a”	537
6. misplaced "e.g." ?	No	Other influences than width and depth are conceivable, such as smooth boundaries and illumination	584
7. demanding enough for	No	From our perception “enough” would suggest a need to create more demanding hydraulic conditions, which was not our intention in this study (see reviewer #1, comment #3).	586f.

Reviewer #3

Reviewer #3: “*In this manuscript, a multi-faceted study is used to develop a framework to model fish movement to test different behavioral rules to explain fish orientation and navigation. The study uses fish tracking data from an unpublished laboratory study of brown trout passage through a partitioned flume. Pattern orienting modelling was used to capture 5 distinct movement patterns. The patterns were used as a metric for which to evaluate the performance of the behavioral rules. The authors use the Eulerian-Lagrangian agent method (ELAM) developed by Goodwin et al. (2014) as a foundation for their individual based model (IBM). The authors IBM was used to evaluate three behavioral rules using five different guidance stimuli. The authors found that wall distance guidance stimulus performed best at replicating the movement patterns.*
Overall, the manuscript was well written and combined with the extensive detail in the appendices (e.g., ODD report) provides a reasonable account of a complex study. Several aspects of the study are not entirely novel. The laboratory study is addressed in a separate manuscript, the CFD analysis is standard practice, and the ELAM provides the general computational infrastructure for the IBM. Therefore, the novelty of this study lies entirely within the behavioral rule and guidance stimulus selection and analysis.
While the sensitivity analysis and modelling output are rigorous, the findings are somewhat muted. As detailed later, I found the laboratory study to be incongruous with the movement hypotheses being tested, likely leading to the less explanatory stimulus of wall distance being the best fit. Despite this issue, I feel the manuscript is still an important finding in the field of predictive modelling of fish movement. Due to the number of comments and level of effort required to address, I recommend the manuscript undergo major revisions.”

**Comment**	**Addressed?**	**How addressed or why not?**	**Line (original)**
Introduction			
1. Line 42 – A reference to Goodwin et al. (2014) would seem appropriate here.	Yes	The reference Silva et al. 2018 serves to underline the “high research priority” of behavioral rules. We did not intend to reference general studies using behavioral rules here, elsewise we would have to list much more than just Goodwin’s. We’ve tried to make this clearer by using a direct quote of Silva et al.	
2. Line 48 – While the introduction touches briefly upon aspects of the fuller study, it	Yes	For choice of behaviors and stimuli see response to comment #1 of reviewer #1.	

lacks sufficient detail to understand why certain selections of behaviors and guiding stimuli were chosen. In this instance, citing the IPOS framework to describe what aspects of turbulence are important to fish behaviors would be relevant.		We've added a reference to IPOS (DOI:10.1002/rra.1584) for general understanding. However, for our IBM study (and others we are aware of), periodicity, orientation, and scale are still too detailed (see comment #5 below and new reference Roth et al. 2021).	
3. What about motivation to move or context specific behaviors? The authors need to address the complexities caused by differences in the internal state of a fish to make different decisions to the same stimuli.	Yes	This is an important aspect and we've added a corresponding paragraph in the introduction. As we focused on orientation and fishway design, we modeled internal stimuli only rather basically (using proxies for motivation and fatigue). We now discuss this in the model description (2.4).	
4. The introduction would further benefit from more details on how the current effort extends or differs from previous IBMs. Specifically, it is not clear how the model is differentiated from the ELAM used by Goodwin et al. (2014). The only reference to the ELAM in on Lines 73 and 84 stating the proposed model is "ELAM-type". I feel the general reader is not going to understand what this means.	Yes	See response to comment #8 of reviewer #1. We also point more directly to lacks of previous upstream IBMs (testing, resting behavior). We've removed the term "ELAM", as it is not required to understand our work.	
5. The authors need to more explicitly state what hypotheses they are testing and why. The preceding paragraphs lists evidence that points to a lot of hydraulic variables that could be influential to fish movement. The authors do not explain why they examine baseline rheotaxis, velocity magnitude, TKE, flow acceleration, and wall distance relative to other choices including turbulent intensity, velocity gradients, eddy sizes, etc.	Yes	See response to comment 1 of reviewer #1. We've added a justification for each stimulus tested in the behavioral model description (2.4) and a summary in the introduction. Generally, we preferred the more comprehensive variable over specialized variables. E.g. all three turbulence intensity components are contained in the TKE (see e.g. IPOS paper, p. 432).<br>The velocity gradient tensor is contained in the acceleration metric.<br>Strain rate and rotation are contained in the velocity gradient tensor.<br>Reynolds shear stresses could be relevant to future work.<br>Eddy size, as well as eddy orientation, is an interesting, but advanced measure left for future work (see response #2 above). It is partly covered in the rheotaxis stimulus.	
Methods			
6. Line 110 – Where does the "x=9.74" come from? Figure 1 indicates an observation point at x=7.49.	Yes	At x=9.74 m, there was an external pole on the flume used as mark for quick orientation in the original study. We've removed this information, as it does not affect this manuscript.	

7. Figure 1. The figure caption is the first mention of patterns P1 and P5 without defining them. I suggest moving this statement into the main text after P1 and P5 are defined.	Yes	We've inserted into the caption a reference to section 2.2 where the patterns are defined. If the caption was changed per your suggestion, readers could be confused about the meaning of the gray areas and line *D*.	
8. Line 109-119 – The methods and scales at which fish movement was tracked is unclear. Where observers tracking movements in real-time on paper as well as noting location relative to the wall or screen or position in a group? How often was the position recorded? The authors state that observations were verified qualitatively with video records, why not use the video to obtain more detailed tracks. Overall, the methods on how tracking was accomplished needs significant more detail.	Yes	We've added this lacking information to the methods (real-time recording on paper; positions were recorded on every notable change; definitions of changes in the three dimensions).<br>We've also added to methods and appendix S1 the information that x_value and y_value were computed in postprocessing.<br>A manual video analysis of fish tracks would have been too time consuming and (because cameras were only positioned laterally) would not have been reliable for lateral movements; we also worked on an automated 3D-fishtracking of the videos (along 11 overlapping cameras) but with three fish swimming simultaneously this is demanding and proved to be less efficient than the manual records for this project.	
9. How were the set-ups chosen for the laboratory tests and how does this relate to the central hypotheses being tested?	Yes	(See also response to comment #15 of reviewer #3 and comment #3 of reviewer #1.)<br><br>The previous study (now available Schütz et al. 2021, https://doi.org/10.1016/j.ecoleng.2021.106257) was not related to our study. It was designed to investigate the influence of auxiliary water velocity coming from the screen on behavior of five species.<br>We've added the word "unrelated" in section 2.1 for clarification.<br>We chose two hydraulically different setups (information added) from this study to test the application range of our model/hypotheses. We did not expect the behavior to be this similar (e.g., Kerr 2016 reported low-energy seeking for trout at smaller U=0.40 m/s).<br>We still found the result interesting, as it expands the common focus of fishways designers on hydraulic stimuli towards vision under certain conditions (possibly low relative flow velocity and visible boundaries).	

10. Line 121-147 – How were these patterns chosen and what hypotheses drove these decisions? Their selection seems somewhat random as written. For example, what details informed splitting the channel at a distance of 0.25 m? Does this distance coincide with an observed hydraulic feature or behavior?	Yes	Added explanation: To capture the most striking spatial behaviors observed (wall and bottom proximity and turns). 0.25 m was chosen arbitrarily to define wall proximity.	
11. Line 155 – Omission of the screen bars is not sufficiently detailed in the methods. I understand the reason for not modelling the bars explicitly, but why not model them as a permeable surface to replicate some of the fine-scale turbulence. See Ho et al. (2011). Ho, J., Coonrod, J., Hanna, L.J., and Mefford, B.W. 2011. Hydrodynamic modelling study of a fish exclusion system for a river diversion. River Res. Applic. 27: 184–192. doi:10.1002/rra.1349.	No	While it is possible to model the screen with bars as a permeable baffle, we avoided the effort of implementation and calibration, as we judged the flow field to be sufficiently close to ADV measurements with respect to our behavioral data accuracy. See also reference Gisen 2018, p. 33ff., for detailed comparisons.<br><br>Regarding fine-scale turbulence, we would expect some kind of damping effect from a permeable baffle, which would require extra attention.	155
12. Line 172 – Again, what underlying hypothesis drove the selection of just these 3 hydraulic variables?	Yes	See response to comment #1 of reviewer #1.	
13. Line 200 – Was distance to the floor included in the wall distance evaluations?	No	No, it wasn't, as the vertical behavior was treated as a separate behavioral rule, which relied on the vertical coordinate (~ pressure) as a stimulus.	
14. Since the behavioral rules and selection of stimuli are the main contributions of this work, I found the model description provided in the main text to be underwhelming. I generally understand the adherence to the ODD protocol, but this should not sacrifice the completeness of the main text to act as a standalone document.	Yes	We've added information on motivation and fatigue, the basic internal states of the model and added a reference (Castro-Santos 2004) to substantiate the importance of repeated passage attempts in the model description.	
Results			
15. Table 1 – The general magnitude and relation of patterns appears to be nearly identical between set-ups. I would even doubt there is any statistical difference between values. This would indicate one of two possible failures in the experimental design: 1). The modifications to the laboratory set-up did not achieve a discernable change in behavior; or 2). The patterns do not adequately capture the behavioral changes caused by the modification to the set-up. Either way, since the authors do not provide any rationale as to what the set-	No	(see also response #9)<br>We agree that our trout behavior as measured by the patterns is very similar between setups. This would be your case (1). However, the previous, unrelated study (Schütz et al. 2021) did not aim to produce different behavior, but was intentionally limited to a typical maximum slot velocity in a multi-species fishway (1.5 m/s). We've added this information in section 2.1.<br>This limitation coincides with the goal of the present study to provide behavioral rules for fishway design. Hence, the only goal of using	

up change was indented to do makes interpretation difficult.		two hydraulically different setups was to quantify the (minimum) application range of our model. Finding the maximum application range would be interesting, but less relevant to design of multi-species fishways, which also need to address species with lesser swimming abilities than trout.	
16. Figure 6 – This figure was very helpful to understand the model results and laboratory data. Additional versions of this figure to compare the modelled movement against observed movement would be beneficial. Illustrations from both set-ups should be included.	No	This figure was intended primarily to foster qualitative understanding. It has little explanatory power, as it depicts only two selected observed and two selected model tracks. Showing more tracks like this would not add quantitative information. Also, a figure showing results for setup 2 would add little additional information (as behavior was not much different). Hence, we would prefer to leave the figure unchanged.	
Discussion			
17. Considering that the observed movement patterns were not largely different between the two set-ups, the conclusion that velocity does not play a role in orientation and navigation is stated too strongly. Based on the available data, it would appear that fish largely exhibited exploratory behavior and followed the walls because the hydraulics did not require any modified behavior. The authors should at least comment that their rule of avoiding high velocities is rather simple. It would not appear to fit the behavioral data either, since passage required movement through an area of elevated velocity in set-up 1. Perhaps a more refined guidance rule surrounding velocity fields is necessary.	No	By no means we intend to state that velocity does not play are role in orientation, generally. However, we found that in our conditions, which are typical for large multi-species fishways built in Germany, brown trout did not use velocity for orientation (probably because it was not demanding enough). We've added explanations to the discussion to clarify this distinction.<br><br>Reviewer #3 is right in the point that orientation towards reduced velocity hinders passage through the jet region and slot in setup 1. It is not impossible, however, because the motivation/fatigue driver for movement is independent of the orientation stimulus and there is also a random component to orientation. Further, it cannot be the single cause for the poor performance of the velocity stimulus version in setup 1, as pattern P5 (which measures slot passage) only contributes 20% to the RMSE metric (section 2.5.1).<br>Still, more refined behavioral rules allowing adaptation to changing flow fields are definitely worthwhile for future work, as discussed in section 4.2.	
18. Line 530-532 – The rationale for not running the model with combinations of stimuli is lacking. I can understand the reason for not	Yes	Our statement was based on comparing the 10th percentile/rank results of the stimulus versions under the assumption of using only one stimulus per setup. It is true that we did	

including this step in this study, but to dismiss their validity is premature. The authors do not explicitly indicate how multiple stimuli could be integrated into a single model, so I do not understand how they can claim the acceleration and velocity versions could not improve upon the wall distance version without explicitly testing this.		not test using multiple stimuli within one setup. However, we argue that velocity and acceleration are not relevant for brown trout orientation in our flume conditions (see comment and response #17), therefore we would expect similar results from a model using both stimuli in the same setup. We rephrased the relevant lines to clarify this point.	
19. Line 589 – The authors repeatedly indicate that wall perception could be accomplished by vision or audition, but it is not entirely clear how or what role audition may play in wall distance perception. I would still expect near field hydrodynamics would override any true acoustical input for most fish. This just strikes me as an odd statement and should be further explained if it remains in the manuscript.	Yes	We agree that this is a hypothetic stimulus on the scales investigated here and removed the word "acoustic" in the abstract and conclusions to make that clearer.	
Appendix S4			
20. Page 4, Para. 1 – The authors state that the relationship between fatigue and swimming speed is not well understood. I find this statement to be highly inaccurate. Numerous studies are available that examine brown trout (and similar salmonids) swimming performance, that is their swim speed vs. duration curves. The authors also site work by Castro-Santos et al. (2005 and 2013) that shows how swimming fatigue could be modelled and swimming performance data for brown trout.	Yes	Agreed, we deleted this statement.	
21. Page 8, Table 3 – How was Ug estimated? Castro-Santos et al. (2013) clearly shows laboratory data on brown trout indicated an optimal ground speed of 5.65 BL/s.	Yes	We estimated the value based on Castro-Santos' 2005 result for striped bass (2.56 BL/s) as stated. We agree that their 2013 paper is a better reference as it deals directly with brown trout. However, note that their value of 5.65 BL/s is a theoretical value for prolonged swim speed and most individuals chose to swim "well below" it (p. 287). Hence, our estimation seems to be in a realistic order. We changed the reference in Table 3, footnote 2, as well as the rationale in section 7.5 accordingly.	
22. Page 10, Section 7.4 – Energy usage is cumulative at the time scale of this study. Once fish expend energy to swim above their sustained swim speed, they are unlikely to re-	Yes	We did not intend to model fatigue and recovery processes in detail, but just enough to drive model movement as measured by the patterns to enable orientation analyses.	

cover within 60 mins. However, since the authors assume no fish became fully exhausted from swimming in the flume, their handling of fatigue as a behavioral metric is understandable. This caveat could be clearer in the main text.		We've mentioned the caveat in section 2.4, and included references to Castro-Santos et al. 2013 and Ebel 2014.	
23. Page 6, Section 4.7 – The authors should comment on whether the random effects encompass stochasticity in animal behavior and the hydraulic stochasticity (i.e., the real flow field is not static).	Yes	We've added the explanation that our behavioral model's stochasticity also covers transient flow field effects on the behavior.	S4 section 4.7

Supporting Information 5

Click here to download Data Review URL https://github.com/baw-de/ELAM-flume
