# Supplementary material for: Development of behavioral rules for upstream orientation of fish in confined space
Source: PLoS One. 2022 Feb 18;17(2):e0263964. doi: 10.1371/journal.pone.0263964 (PMC8856537; doi:10.1371/journal.pone.0263964)
Supplement: Attachment — Submitted filename: Response to reviewers.docx [file pone.0263964.s010.docx]

Response to reviewers

Again, thank you for your helpful comments which improved the manuscript.

We note that the manuscript does not yet meet the style requirements of PLOS ONE, but decided to await the provisional Editorial Accept decision (per <https://journals.plos.org/plosone/s/getting-started>) to avoid confusion during review e.g. by changing the citation style.

We’ve added a new reference (Goodwin et al. 2006) per suggestion of reviewer #2. Line numbers refer to the manuscript without recent changes, submitted in July.

# Reviewer #2:

The manuscript is much improved.

| **Comment** | **Ad dres sed?** | **How addressed or why not?** |
| --- | --- | --- |
| I have, again, made comments and suggestions (annotations) directly into the manuscript PDF itself, attached as part of this review. | Y | See Manuscript With Track Changes for small (e.g. grammatical) corrections. Changes not adopted in full are listed and commented below. |
| There are still a few areas where the authors should add clarity in the narration of their results, especially related to parameter testing. In short, there is a lot of nuance in the parameter analyses and I found it difficult at times to keep track of all the different nuances moving from one paragraph to another. Perhaps consider reminding the reader of some of the basics (setup) which is then elaborated in great detail/nuance within the paragraph. | Y | See detailed responses below and related sections in the methods (2.5.3-2.6.3). |
| Line 11 – Why? | N | Orientation is essential to find, enter, and pass a fishway. |
| Line 20 – Why? | Y | Rheotaxis is a fundamental behavior for directed navigation and could therefore be expected to be important for modeling upstream movement, ll. 53-55. As this information seems to be more confusing than helpful at this point, we’ve deleted it. |
| Line 21 - ? | N | The baseline version performed quite well using the most simple (baseline) orientation rules (rheotaxis + random component). |
| Line 23 – features evaluated | Y | these flow features |
| Line 42 – very closely | Y | The point of this paragraph is to substantiate this claim and to use it as an argument for the suitability of IBMs for these kind of problems. Replaced by “rather accurately”. |
| Line 64 – spatial velocity gradient | Y | Added reference to Goodwin et al. 2006 and the spatial velocity gradient. We did not include a reference to Nestler et al. 2008 as the paragraph centers on practical use in IBMs. |
| **Methods** |  |  |
| Line 200 - ? | N | “entities” is a keyword of the ODD protocol (Grimm et al. 2020) |
| Line 215 – skin? | Y | Added “imaginary” and “real fish” for clarification. |
| Line 218 - ? | Y | Removed the word “balance” to describe the underlying process more precisely. Omitted “driving and retaining”. Changed “traits” to “characteristics”, as it can have different meanings in biology and modeling. Same changes in appendix S4, model description. |
| Lines 322-325 – Why? / I don’t understand | Y | Removed unnecessary detail, added explanation and separated sensitivity and version tests more clearly. |
| Lines 338-339 – confusing | Y | Rephrased, removed selection of the stimulus version to avoid confusion. The rationale is given in the end of section 3.4 when the test result is known. |
| Lines 348-349 – confusing | Y | Deleted unnecessary detail and rephrased |
| Lines 358-369 – Suggest reminding readers of some of the general stuff at the beginning of each paragraph, because it’s not trivial to track all the nuances from paragraph to paragraph. | Y | Improved explanation, added link to previous section, and streamlined key word “stimulus version” |
| Line 372 - ? | Y | Improved explanation and link to previous section |
| Line 376 - ? | Y | Improved explanation |
| Line 422 – Again, remind readers of some of the basics…as after several pages of sensitivity analysis the larger (more important) story you’re trying to tell may get lost. | Y | Added (repeated) the main goal of sensitivity analysis, more precise descriptions, smoother transition between paragraphs. |
| Line 431 – Is it really plunging? | Y | Replaced “plunging jet” with “head drop at the slot” |
| Line 432 – Confusing | Y | Rephrased |
| Line 445 – Accordance | Y | Agreement |
| Line 467 - But that was arbitrary and based on underlying passage proportions between migratory routes. Goodwin is probably not relevant here. | Y | Our intention was to give naïve readers a ballpark estimate of achievable precision in fish movement IBMs. Deleted to avoid confusion. |
| Line 467 – Explain, this is a bold statement | Y | *All* referred to “captured by our patterns”, but is dubious when RMSE it not zero. Deleted. |
| Line 490 - or space was too confined for an animal that’s evolved to use behavior in much, much larger domains of river widths. | N | The brown trout were caught in a small tributary of the Rhine river (Gisen 2018), the Alb, which is 10-20 m wide and has a mean discharge of ~2-5 m³/s. We are convinced that similar rivers also played a role in shaping their behavior.  If the trout really did behave in the flume like they would in nature is a fundamental question in any kind of laboratory observation. We’ve addressed the topic of transferability e.g. by limiting our conclusions to situations with visible walls. |
| Line 630 – delete “e.g.” | N | Other examples of low velocity include certain areas of nature-like fishways as well as lab and field studies. |

# Reviewer #3:

In general, the authors addressed most of the reviewer comments well. Of greatest benefit is the additional discussion on the model limitations. The introduction follows a more logical path and the justification of selected behaviors is much improved. While I still find the experimental setups and movement tracking methods to be less than ideal, I find the modelling results to be worthwhile additions to the body of literature. Specific comments are noted below with the corresponding line number.

| **Comment** | **Ad dres sed?** | **How addressed or why not?** |
| --- | --- | --- |
| Line 87 – I feel that a major contribution of this study is the use of movement parameters to evaluate model performance in a more objective manner than qualitative trajectory comparisons. The authors seem to be hinting at that with this statement, but further details should be added here. Regardless, a paragraph needs to be more than a single sentence. | Y | Added details about qualitative and quantitative testing. |
| Line 90 – “For the first time” is an unnecessary detail that exaggerates the novelty of the study. | Y | Rephrased to convey the major innovations more moderately. |
| Line 141 – From the supplementary data, it is clear that observations of trout swimming positions were relatively coarse (> 5 sec) especially in comparison to the output of the modelled positions (0.5 sec). Based on the methods provided, it is unclear how the authors dealt with the difference in temporal resolution for deriving the movement parameters. I suspect this could influence the RMSE values especially with the relatively small spatial region covered by the P1 left and right zones. | N | As described in l. 125, observations were recorded upon notable change, i.e. all relevant movement was captured independent of its timing.  A direct influence of different track resolution was prevented through temporal normalization by track duration for both laboratory and model data (ll. 148-150, 295-296).  We acknowledge limitations resulting from the temporal (and spatial) resolution of the observations (ll. 599-602). However, note that the relatively small zones for P1 were chosen precisely because of the distinct wall attachment and little lateral movement. |
| Line 196 – Delete “e.g.” | Y | Inserted “that could” |
| Line 296 – Insert “an” before “evaluation metric”. | Y | Inserted “an” |
| Line 420-421 – It is interesting that time step selection had such an influence on model performance, and that is varied between setup, especially for static model environment. The authors should expand on this finding in the discussion. | Y | In our view, even the larger time step tested (*Δt* = 1.0 s) should be sufficiently fine to meet our requirements regarding decision and perception frequency. As it was arbitrarily chosen in the beginning, it likely became part of the parameter calibration. This explains the time step sensitivity, but is not relevant to our comparison of rules for swim angle selection. Added this interpretation to section 4.4. |
| Line 445 – Insert “in” before “accordance”. | Y | Replaced with “agreement” |
| Line 449 – 496 – The method used by Zielinski et al. (2018) only enforced path selection based on energy conservation when fish swam at prolonged and burst swimming speeds, which does not appear to be the case in either setup in this study. | Y | Thanks for pointing that detail out which renders it a less supporting reference here. Changed reference to Kerr et al. 2016, who seem to have a similar flow velocity/body length ratio compared to our study with the difference that they observed energy conservation behavior. |
| Line 565 – 578 – I think a critical omission in this discussion in the findings of Goodwin et al. (2014) which found the best fitting behavioral model integrated 4 separate behaviors. Without performing runs with multiple behaviors possible, the authors seem to over reach with their dismissal of hydraulic cues influencing brown trout movement. While I agree that hydraulically mediated behaviors are unlikely to be observed in setup 2, certainly as trout near the vertical slot in setup 1 movement should be more complex than simple wall following. I also agree that simplified models, in general, provide the greatest explanatory power and exportability, but this alone should not prevent exploration into more complex models. | N | We did not discuss details of the behavioral model of Goodwin et al. 2014 here, as it was designed for downstream migration of juvenile smolts and is not applicable to our conditions including migration direction, species, and confinement (Gisen 2018).  We discuss the topic of stimulus combinations for our stimuli in section 4.2 with the result that no improvement can be expected for our conditions, while acknowledging that this might be different for the general case.  We agree that a more complex behavior model would be needed to fully capture behavior near (1-2 m) the slot in setup 1. As far as it is known, entering a fishway (slot) requires different behavior than finding it (e.g. Bunt et al. 2012). However, both recording and modeling such behavior would require a new level of detail compared to the remainder of the flume. We are aware of this limitation and point to it in section 4.4. |
